# Toxicity of Nanoparticulate Nickel to Aquatic Organisms: Review and Recommendations for Improvement of Toxicity Tests

**DOI:** 10.1002/etc.4812

**Published:** 2020-08-25

**Authors:** Joseph S. Meyer, Tara Lyons‐Darden, Emily R. Garman, Elizabeth T. Middleton, Christian E. Schlekat

**Affiliations:** ^1^ Applied Limnology Professionals, Golden Colorado USA; ^2^ NiPERA Durham North Carolina USA

**Keywords:** Acute, Chronic, Dissolution, Nickel, Complex engineered nanomaterials, Size

## Abstract

We reviewed the literature on toxicity of nanoparticulate nickel (nano‐Ni) to aquatic organisms, from the perspective of relevance and reliability in a regulatory framework. Our main findings were 1) much of the published nano‐Ni toxicity data is of low or medium quality in terms of reporting key physical–chemical properties, methodologies, and results, compared with published dissolved nickel studies; and 2) based on the available information, some common findings about nanoparticle (NP) toxicity are not supported for nano‐Ni. First, we concluded that nanoparticulate elemental nickel and nickel oxide, which differ in chemical composition, generally did not differ in their toxicity. Second, there is no evidence that the toxicity of nano‐Ni increases as the size of the NPs decreases. Third, for most organisms tested, nano‐Ni was not more toxic on a mass‐concentration basis than dissolved Ni. Fourth, there is conflicting evidence about whether the toxicity is directly caused by the NPs or by the dissolved fraction released from the NPs. However, no evidence suggests that any of the molecular, physiological, and structural mechanisms of nano‐Ni toxicity differ from the general pattern for many metal‐based nanomaterials, wherein oxidative stress underlies the observed effects. Physical–chemical factors in the design and conduct of nano‐Ni toxicity tests are important, but often they are not adequately reported (e.g., characteristics of dry nano‐Ni particles and of wetted particles in exposure waters; exposure‐water chemistry). *Environ Toxicol Chem* 2020;39:1861–1883 © 2020 The Authors. *Environmental Toxicology and Chemistry* published by Wiley Periodicals LLC on behalf of SETAC.

## INTRODUCTION

Nanoparticles (NPs; particles having at least 1 dimension in the size range 1–100 nm) have become popular in industry and research because of unique mechanical, optical, chemical, and electronic processes that can occur at a small scale (Howard 2010), and they are being used at exponentially increasing rates (e.g., a compound annual market growth rate of >22%; Inshakova and Inshakov 2017). As a result of their increasing use and high reactivity, there is concern about the potential ecological effects of NPs (Handy et al. 2008b; Klaine et al. 2008; Lead et al. 2018). Although many reviews of the toxicity of NPs to aquatic organisms have been published, they mostly focus on the more extensively used nanomaterials (e.g., carbon, copper, iron, silver, silicon, titanium, zinc; Mohanty et al. 2014; Ma et al. 2015; Kahlon et al. 2018; Lead et al. 2018). Thus, reviews are limited for less extensively used and less researched nanomaterials such as those containing nickel (Ni).

Materials containing engineered nanoparticulate Ni (which we have termed nano‐Ni as a general category) are manufactured in several forms, including powders consisting of nearly spherical or irregularly shaped individual particles composed mainly of elemental (zero‐valent) Ni (nano‐Ni^0^) or nickel oxide (nano‐NiO), and powders or sheets of complex engineered nanomaterials (CENs) containing Ni and other metallic elements or organic carbon–containing chemicals. These materials have a variety of commercial and research uses, such as biosensing, catalytic converters in automobiles, other chemical catalysis, conductive coatings, data storage, lithium ion batteries, magnetic fluids, fuel cells, microelectronics, optical technology, packaging, propellant additives, sintering additives, solar cells, and water purification (e.g., AZoNano 2013; Schrittwieser et al. 2018). Because of the variety of manufacturing processes and waste streams, types and locations of use, types and amounts of wear during use, and methods of disposal, there is concern that engineered nanomaterials in general (and thus nano‐Ni) can enter ecosystems and pose ecological risks that need to be balanced against their benefits to society (e.g., Handy et al. 2008a).

**Figure 1:**
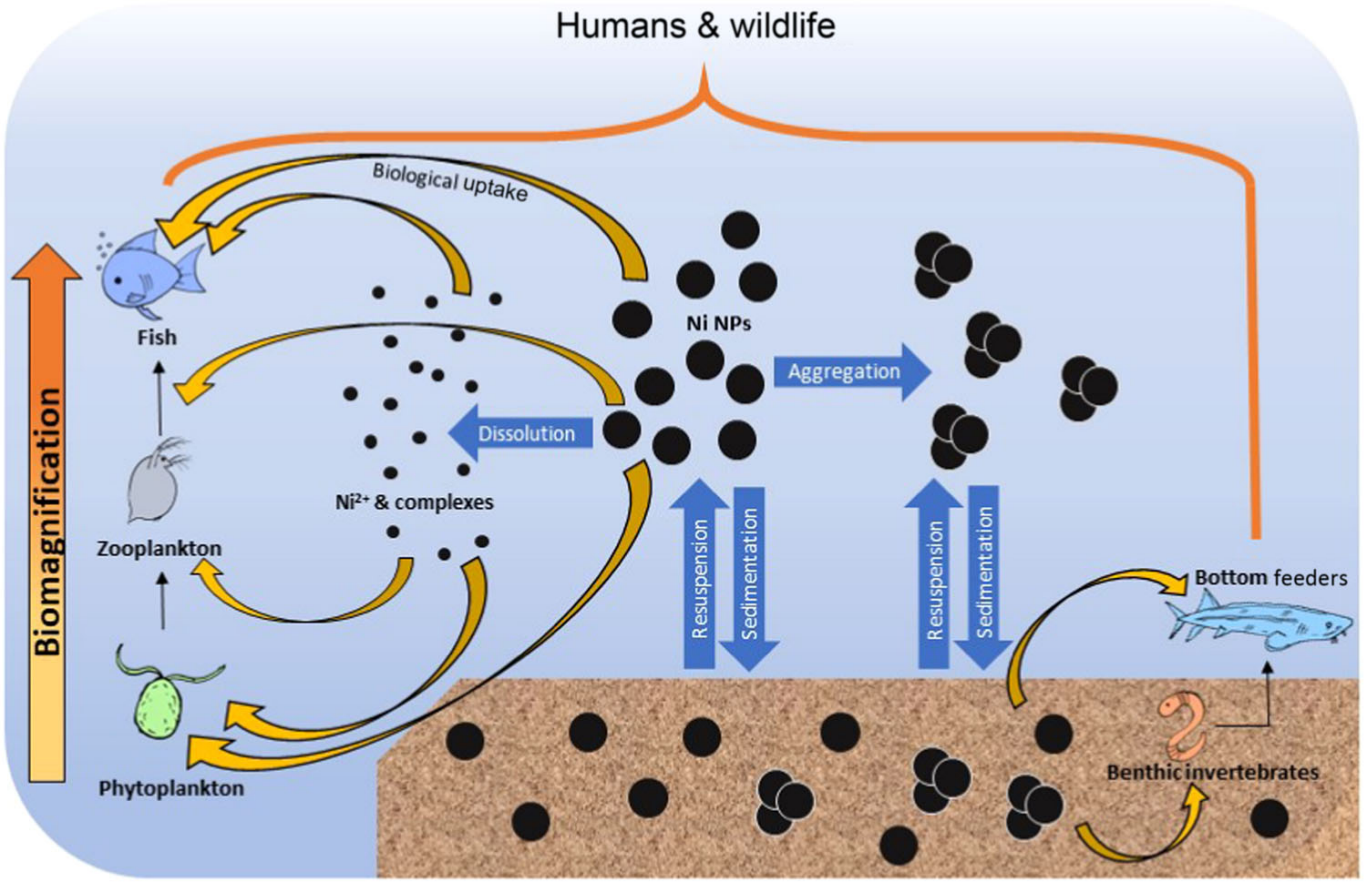
Fate of biologically relevant nickel‐containing nanoparticles (NPs) in aquatic systems (adapted from Biswas et al. 2018).

In particular, aquatic organisms can be exposed to nano‐Ni via direct discharges to surface waters by manufacturers and users, leaching from landfills into which nano‐Ni materials have been discarded, erosion of nano‐Ni deposited onto the surface of soils, and deposition of aerially suspended nano‐Ni onto surface waters. The fate of NPs in a water body can be multifaceted (Figure 1). After entering a water body, nano‐Ni particles suspended in the water column can be ingested by organisms, attach to their external membranes, or be internalized across membranes, thus causing “direct” toxicity (see *Mechanisms of nano‐Ni toxicity*). In addition, nano‐Ni particles can partially or totally dissolve while in the water column or after having settled to the sediment, thus leading to “indirect” toxicity by exposing organisms to dissolved Ni released from the particles, which itself can be toxic (Buxton et al. 2019). And finally, NPs can 1) directly settle out of the water column, and/or 2) agglomerate and then settle out of the water column onto the sediment, where benthic organisms can ingest or internalize the sedimented particles and thereby directly interact with nano‐Ni.

Because of these concerns about potential ecological effects of nano‐Ni, we have reviewed the available literature on the toxicity of nano‐Ni to aquatic organisms; evaluated the existing data for relevance and reliability; and recommended improvements to the design, analysis, and reporting of toxicity tests and physiological experiments in which aquatic organisms are exposed to nano‐Ni and other NPs. Unlike many other review papers, we have emphasized relevance and reliability, and we analyzed nano‐Ni toxicity data from the perspective of their potential use in regulatory and risk assessment settings. In addition, we discuss physiological effects (e.g., production of reactive oxygen species [ROS], alteration of enzyme activities) underlying the toxic effects of nano‐Ni that constitute traditional regulatory concerns (e.g., survival, growth, reproduction). However, this meta‐analysis is limited by the same general challenges in evaluating the toxicity of other nanomaterials, commonly including 1) lack of testing with numerous chemical forms of nano‐Ni, 2) insufficient toxicity data across taxonomic groups, 3) lack of use of standardized study designs and testing methods, and 4) insufficient reporting of study designs, testing methods, physical–chemical exposure conditions, and toxicity results (e.g., see reviews by Baun et al. 2008; Handy et al. 2008c; Klaine et al. 2008; Handy et al. 2012a, 2012b; Kühnel and Nickel 2014; Ma et al. 2015; Holden et al. 2016; Hund‐Rinke et al. 2016; Reddy et al. 2016; Peng et al. 2017; Lead et al. 2018).

We focused mainly on the 2 most studied forms of nano‐Ni (nano‐Ni^0^ and nano‐NiO), but results of toxicity tests with other forms of nano‐Ni (e.g., LiCoNiMn CENs) are included where appropriate. Because several common findings tend to guide thinking about NP toxicity, we explored whether the available information supports that 1) different chemical forms of nano‐Ni (i.e., nano‐Ni^0^ and nano‐NiO) differ in their toxicity, 2) the toxicity of nano‐Ni depends on the dry size of the NP, and 3) nano‐Ni is more toxic than dissolved Ni. In addition, we compared reported molecular, physiological, and structural mechanisms of nano‐Ni toxicity with general concepts of NP toxicity, and explored whether 1) nano‐Ni particles or the dissolved Ni released from the particles during toxicity tests cause the observed toxicity, 2) nano‐Ni toxicity differs among taxa and among life stages, and 3) physical factors in the design of the tests (illumination of the test chambers, and presonication of the exposure waters) influence nano‐Ni toxicity.

## MATERIALS AND METHODS

### Overview

For this meta‐analysis, we first conducted a literature search to identify publications related to the aquatic toxicity of nano‐Ni. Second, we screened those publications for relevance and reliability, to narrow the compilation to a subset that might be more appropriate for use in regulatory and risk assessment applications. Third, we processed the toxicity results in that subset of publications to identify standard acute and chronic toxicity metrics or, when those metrics were not reported, to calculate those metrics if sufficient data were provided. Fourth, we tabulated the toxicity metrics by test organism and by chemical form of the nano‐Ni tested, and then interpreted the results. In the following 3 sections, we summarize the methods used in the literature search, data‐quality screening, and processing of toxicity data. The Supplemental Data contains 1) the abbreviations and definitions we used (*List of Abbreviations* and *Nanomaterial Definitions* sections), and 2) additional details about the data‐quality screening and the processing of toxicity data (*Quality screening of literature* and *Processing of toxicity data* sections in the Supplemental Data text and Table S2).

### Literature search

Relevant aquatic toxicity publications were located in 2 ways. First, on‐line databases were searched to identify publications related to nanomaterials (keyword “nano*”), Ni (keyword “nickel”), and toxicity (keyword “toxic*”). Those databases included ProQuest, Web of Science, and Google Scholar. Second, reference lists in the accessed primary research publications and review articles were inspected for additional relevant publications. The on‐line searches were conducted from January 2018 through October 2019. In addition, the tables of contents of 47 scientific journals related to nanomaterials and environmental toxicology were electronically searched (Supplemental Data, Table S1). We also located publications that compared the toxicity of nano‐Ni with the toxicity of dissolved Ni salts.

### Quality screening

The compiled publications were sorted into 2 groups: those that included primary reports of toxicity tests and physiological experiments, and those that did not (i.e., secondary reports such as review articles). Only primary articles were included in the subsequent review, and we screened them for relevance (i.e., appropriate test organisms, exposure pathways, and toxicity endpoints for use in regulatory applications) and reliability (i.e., data quality).

Major taxonomic groups of test organisms that might occur in aquatic systems were defined for this meta‐analysis as fish, amphibians, aquatic invertebrates, aquatic plants, aquatic macroalgae and microalgae, yeasts, fungi, bacteria, and viruses. The only exposure pathway included in the present meta‐analysis was waterborne exposure to nano‐Ni. Relevant toxicity endpoints included measures of survival, somatic growth, reproduction, and population growth (e.g., increased optical density in exposure water or growth medium containing bacteria, fungi, yeasts, or algae).

The 3 criteria (test organism, exposure pathway, and toxicity endpoint) were incorporated into an evaluation of the study design for each toxicity test, in addition to consideration of whether a series of NP concentrations and a negative control were used. That study design evaluation was the first of 10 categories in the quality‐rating system we used to evaluate each toxicity test (Supplemental Data, Table S2). The other 9 categories in the quality‐rating scheme were related to reporting of physical–chemical properties of the NPs and conduct and reporting of toxicity test methods and results. The ratings of all 10 categories were averaged to produce an overall rating of low (“L”), medium (“M”), or high (“H”) for each toxicity test. Toxicity tests that received an overall rating of H or M were used in our meta‐analysis; tests that were rated as L were excluded. In the *Results and Discussion*, *Recommendations*, and *Conclusions* sections, we suggest ways to improve aquatic toxicity studies with NPs that would help to increase their quality ratings.

### Processing of toxicity data

Consistent with the approach used in most regulatory and risk assessment settings, we divided the toxicity tests into acute and chronic exposure durations. Because chronic toxicity data are favored in many regulatory jurisdictions (e.g., Canada; Canadian Council of Ministers of the Environment 2007), Europe; European Chemicals Agency 2008), there is a general preference for those studies; however, we have also included acute toxicity data because of their predominant use in some other jurisdictions (e.g., within the United States). In the present analysis, acute toxicity results are only represented by the median lethal (or effect) concentrations (L[E]C50), whereas chronic toxicity results are reported as a threshold effect concentration (TEC) represented either by a 10% lethal (or effect) concentration (L[E]C10) or by a chronic value or maximum allowable toxicant concentration (ChV or MATC; the geometric mean of the no‐observed‐effect concentration [NOEC] and the lowest‐observed‐effect concentrations [LOEC]). We also tabulated minimum inhibitory concentrations (MICs) that were reported in some toxicity tests with microbes.

In the present analysis, reported effects monitored in the entire set of toxicity tests included somatic growth, population growth (only for bacteria, fungi, yeasts, and algae), reproduction, hatching, morphological development of larvae (only invertebrates), photosynthetic rate (1 plant), bioluminescence (only bacteria), metabolism as an index of cell viability (only bacteria), and nitrogen removal rate (microbial assemblages). However, for calculation of acute and chronic toxicity metrics, effects that are more traditionally considered as biomarkers in adverse outcome pathways not directly associated with survival, growth, or reproduction were not included as endpoints (e.g., genomic and physiological responses, photosynthetic rate, bioluminescence, metabolism as an index of cell viability, nitrogen removal rate).

Although most authors reported exposure concentrations as mg of nanomaterial/L (or sometimes as the equivalent units of μg of nanomaterial/mL), we converted all concentrations of Ni‐containing nanomaterials to units of mg Ni/L. For example, at the extremes, Ni constitutes 79% of the total mass of NiO but only 26% of the total mass of NiCl_2_·6H_2_O, both of which were used in the toxicity tests we reviewed. The conversion of concentration units to a common basis of mg Ni/L allows a more direct comparison of Ni content among different chemical forms of nano‐Ni (e.g., between nano‐Ni^0^ and nano‐NiO) and between nano‐Ni and dissolved Ni salts, thus allowing more appropriate toxicity comparisons.

## RESULTS AND DISCUSSION

### Composition of the database

Chemical forms of nano‐Ni and the taxa that have been used in nano‐Ni toxicity tests are listed in the Supplemental Data, Tables S3 and S4. Quantitative results (L[E]C50s, TECs, MICs) of toxicity tests that were conducted in an aqueous‐based medium (e.g., not on or in solidified agar) are listed in the Supplemental Data, Tables S5 (nano‐Ni^0^), S6 (nano‐NiO), and S7 (Ni‐containing CENs), where the final overall rating of each toxicity test (L, M, or H) is also indicated. Results of toxicity tests with nano‐Ni^0^ and nano‐NiO that survived the quality screening (i.e., with a final overall rating of M or H) are summarized by test organism in Tables 1 and 2. Studies not included in the present meta‐analysis because they received a final overall rating of L are listed in the Supplemental Data, Table S8, along with reasons for that rating. Details of the scores assigned in each of the individual rating categories for each study are contained in the Supplemental Data, Table S10 (the Supplemental Data Excel file).

**Table 1:** Geometric means (geomeans) and ranges of individual values of median lethal (or effect) concentrations (L[E]C50s) in acute toxicity tests and threshold effect concentrations (TECs) in chronic toxicity tests with freshwater and saltwater organisms exposed to nanoparticulate elemental nickel (nano‐Ni^0^); only tests for which the quality‐screening rating exceeded “low” are included^a^

Taxon and endpoint	Water^b^	Geomean acute L(E)C50 of nano‐Ni^0^ (mg Ni/L) [range of values; *n*]^c^	Acute rank^d^	Geomean chronic TEC of nano‐Ni^0^ (mg Ni/L) [range of values; *n*]^e^	Chronic rank^f^
*Acartia tonsa* (calanoid copepod) Acute: 48‐h naupliar mortality Chronic: 7‐d survival of early life stage	SW	21.1 [20.2–22.1; *n* = 2]	11	4.11 [3.77–4.48; *n* = 2]	4
*Bacillus subtilis* (bacteria) Acute: 6‐h population growth Chronic: 6‐h population growth	FW	<20 [*n* = 1]	10	<20 [*n* = 1]	5
*Ceriodaphnia dubia* (water flea) Acute: 48‐h survival of neonates Chronic: none	FW	0.674 [*n* = 1]	2	—	—
*Ciona intestinalis* (sea squirt; ascidian) Acute: 24‐h morphological development of larvae^g^ Chronic: none	SW	16.8 [*n* = 1]	9	—	—
*Danio rerio* (zebrafish) Acute: 72‐h hatching of embryos^h^ Chronic: none	FW	30.8 [*n* = 1]	12	—	—
*Daphnia magna* (water flea) Acute: 48‐h survival of neonates Chronic: none	FW	5.68 [*n* = 1]	5	—	—
*Daphnia pulex* (water flea) Acute: 48‐h survival of adults Chronic: none	FW	3.89 [*n* = 1]	4	—	—
*Escherichia coli* (bacteria) Acute: 24‐h population growth^i^ Chronic: 24‐h population growth^i^	FW	<10.6 [6.03–<20; *n* = 3]	7	<3.54 [0.222–<20; *n* = 3]	3
*Paracentrotus lividus* (purple sea urchin) Acute: 48‐h growth of nauplii^j^ Chronic: none	SW	>3 [*n* = 1]	3	—	—
*Pseudokirchneriella subcapitata* (green alga) Acute: 96‐h population growth Chronic: none	FW	0.35 [*n* = 1]	1	—	—
*Staphylococcus aureus* (bacteria) Acute: 24‐h population growth Chronic: 24‐h population growth	FW	11.6 [*n* = 1]	8	0.430 [*n* = 1]	1
*Streptococcus mutans* (bacteria) Acute: 24‐h population growth Chronic: 24‐h population growth	FW	10.4 [*n* = 1]	6	0.476 [*n* = 1]	2

^a^ The L(E)C50s and TECs in individual studies used in calculation of the geomeans are listed in the Supplemental Data, Tables S5 and S6.

^b^ FW = freshwater‐based; SW = saltwater‐based.

^c^ *n* = number of L(E)C50s or TECs used to calculate the geometric mean L(E)C50 or TEC.

^d^ Ranked from lowest (rank 1) to highest values (rank 12).

^e^ TECs are: 10% lethal (or effect) concentrations (L[E]C10s), geometric means of the no‐observed‐effect concentration (NOEC) and lowest observed effect concentration (LOEC), or geometric means of the minimum inhibitory concentration (MIC) and the next lower exposure concentration (only for bacteria and fungi). For each toxicity test or experiment, the lowest value of the 3 endpoints (L[E]C10, geometric mean of NOEC and LOEC, or geometric mean of MIC and the next lower exposure concentration) is used in the TEC calculation. *n* = number of TECs used to calculate the geometric mean TEC.

^f^ Ranked from lowest (rank 1) to highest values (rank 5).

^g^ In the absence of survival data for *C. intestinalis*, morphological development of an early life stage (which is expected to be a more sensitive endpoint than survival of the same organisms) is used as the toxicity endpoint.

^h^ Survival is not used as the exposure endpoint for *D. rerio* because the EC50s for embryo hatching generally were approximately 10‐fold lower than the LC50s for survival.

^i^ Bioluminescence endpoint is not included because population growth endpoint was generally more sensitive.

^j^ In the absence of survival data for *P. lividus*, growth of an early life stage (which is expected to be a more sensitive endpoint than survival of the same organisms) is used as the toxicity endpoint.

**Table 2:** Geometric means (geomeans) and ranges of individual values of median lethal (or effect) concentrations (L[E]C50s) in acute toxicity tests and threshold effect concentrations (TECs) in chronic toxicity tests with freshwater and saltwater organisms exposed to nanoparticulate nickel oxide (nano‐NiO); only tests for which the quality‐screening rating exceeded “low” are included^a^

Taxon and endpoint	Water^b^	Geomean acute L(E)C50 of nano‐NiO (mg Ni/L) [range of values; *n*]^c^	Acute rank^d^	Geomean chronic TEC of nano‐NiO (mg Ni/L) [range of values; *n*]^e^	Chronic rank^f^
*Artemia salina* (brine shrimp) Acute: 24‐h survival of larvae Chronic: none	SW	>23.0 [>15.8–33.6; *n* = 2]	7	—	—
*Bacillus anthracis* (bacteria) Acute: 20‐ to 24‐h population growth Chronic: 20‐ to 24‐h population growth	FW	2.90 [*n* = 1]	2	2.28 [*n* = 1]	4
*Brachionus plicatilis* (saltwater rotifer) Acute: 48‐h survival of neonates Chronic: none	SW	>15.8 [*n* = 1]	6	—	—
*Chlorella vulgaris* (green alga) Acute: 72‐h population growth Chronic: 72‐h population growth	FW	30.4 [*n* = 1]	8	13.2 [*n* = 1]	8
*Danio rerio* (zebrafish) Acute: 67‐ to 120‐h hatching of embryos^g^ Chronic: 30‐d survival of adults	FW	<39.4 [<39.4; *n* = 3]	9	11.9 [*n* = 1]	7
*Daphnia magna* (water flea) Acute: 48‐h survival of neonates Chronic: 21‐d reproduction	FW	12.0 [7.67–29.0; *n* = 3]	5	0.0794 [0.0268–0.176; *n* = 3]	1
*Escherichia coli* (bacteria) Acute: 8‐ or 24‐h population growth^h^ Chronic: 8‐ or 24‐h population growth^h^	FW	2.15 [1.34–3.46; *n* = 2]	1	0.361 [0.254–0.512; *n* = 2]	2
*Lemna minor* (common duckweed) Acute: none Chronic: 7‐d frond growth	FW	—	—	<4.10 [<4.10; *n* = 2]	6
*Pseudokirchneriella subcapitata* (green alga) Acute: 72‐h population growth Chronic: 72‐h population growth	FW	4.72 [1.26–12.5; *n* = 3]	4	<3.76 [0.867–9.46; *n* = 3]	5
*Saccharomyces cerevisiae* (yeast) Acute: 6‐ or 24‐h cell viability or population growth Chronic: 6‐ or 24‐h cell viability or population growth	FW	>84.2 [78.8–92.7; *n* = 4]	10	∼54.7 [37.2–>78.8; *n* = 4]	9
*Staphylococcus aureus* (bacteria) Acute: 8‐ or 24‐h population growth Chronic: 8‐ or 24‐h population growth	FW	3.73 [2.68–5.18; *n* = 2]	3	1.03 [0.874–1.21; *n* = 9]	3

^a^ L(E)C50s and TECs in individual studies used in calculation of the geomeans are listed in the Supplemental Data, Tables S5 and S6.

^b^ FW = freshwater‐based; SW = saltwater‐based.

^c^ *n* = number of L(E)C50s or TECs used to calculate the geometric mean L(E)C50 or TEC.

^d^ Ranked from lowest (rank 1) to highest values (rank 10).

^e^ TECs are: 10% lethal (or effect) concentrations (L[E]C10s), geometrics means of the no‐observed‐effect concentration (NOEC) and lowest‐observed‐effect concentration (LOEC), or geometric means of the minimum inhibitory concentration (MIC) and the next lower exposure concentration (only for bacteria and fungi). For each toxicity test or experiment, the lowest value of the 3 endpoints (L[E]C10, geometric mean of NOEC and LOEC, or geometric mean of MIC and the next lower exposure concentration) is used in the TEC calculation. *n* = number of TECs used to calculate the geometric mean TEC.

^f^ Ranked from lowest (rank 1) to highest values (rank 9).

^g^ Survival is not used as the exposure endpoint for *D. rerio* because the EC50s for embryo hatching generally were approximately 10‐fold lower than the LC50s for survival.

^h^ Bioluminescence endpoint is not included because population growth endpoint was generally more sensitive.

One hundred primary research publications reported a total of 304 combinations of nano‐Ni form × biological species × publication (Supplemental Data, Table S4). A total of 307 tests contained toxicity and/or physiology information for 73 taxa exposed to a total of 38 chemical forms of nano‐Ni. However, only 31 publications passed the quality screening, narrowing the number of taxa included in the present meta‐analysis to 22. Nano‐Ni^0^ and nano‐NiO were used most frequently in the toxicity tests (32% nano‐Ni^0^, 38% nano‐NiO, 28% Ni‐containing CENs, 2% other nano‐Ni‐oxides/hydroxides). The main deficiencies that eliminated studies included conducting exposures to nano‐Ni in or on solidified agar (thus making it challenging to translate those results into meaningful aqueous exposure concentrations), not adequately describing the toxicity test method or not referring to a standardized test method, not adequately reporting exposure‐water chemistry, and not reporting control acceptability. Forty‐one (13%) of the 307 toxicity tests (in 9 of the 100 publications) had unspecified test durations.

Additional details are in the *Composition of database* section in the Supplemental Data.

### Study quality

Most nano‐Ni toxicity studies reviewed for the present meta‐analysis had several deficiencies in the design of the study and/or reporting of results. The most important are mentioned in the following paragraph. Additional details are in the *Study quality* section in the Supplemental Data.

Of the 304 combinations of nano‐Ni form × biological species × publication listed in the Supplemental Data, Table S4, 122 were tested only in or on solidified agar, and the exposure medium was not specified in another 2. Those results are not included in the summary results in Tables 1 and 2 and in the individual test results in the Supplemental Data, Tables S5 and S6. Of the 307 toxicity tests, measured nano‐Ni concentrations were reported in only 2. In other words, in >99% of the tests, the reported nano‐Ni concentrations were nominal (i.e., they were based only on the amount of nano‐Ni intended to be added to the exposure waters). In addition, many authors did not adequately report test methods, characteristics of the NPs and/or the exposure water/medium, the extent of particle settling, the amount of dissolution of the NPs, and at least minimal quantitative toxicity results or summary metrics. The importance of reporting measured concentrations and characteristics of the NPs and/or the exposure water/medium is discussed in the *Uncertainties in nano‐Ni exposure during toxicity tests* and the *Recommendations* sections.

### Toxicity of nano‐Ni

The L(E)C50s and TECs from all the individual toxicity tests with nano‐Ni are listed in the Supplemental Data, Tables S5 and S6, respectively; and the associated concentration–response data and resulting regression curves are shown in the Supplemental Data, Figures S1 through S77. Geometric mean acute L(E)C50s and chronic TECs from all toxicity tests conducted with aquatic organisms that were exposed to nano‐Ni^0^ and nano‐NiO and that passed the quality screening are listed in Tables 1 and 2. The acute L(E)C50s and chronic TECs from all toxicity tests conducted with aquatic organisms exposed to Ni‐containing CENs (including those that did not pass the quality screening) are listed in the Supplemental Data, Table S7.

#### *Nano‐Ni* ^*0*^ *L(E)C50s and TECs for nano‐Ni* ^*0*^ *, nano‐NiO, and nano‐Ni‐oxides/hydroxides*.

Most of the reported acute L(E)C50s for nano‐Ni^0^ in Table 1 exceeded 1 mg Ni/L, and all exceeded 0.1 mg Ni/L. The taxon‐specific geometric mean L(E)C50s ranged from 0.35 to 98.7 mg Ni/L, with geometric mean L(E)C50s for the 2 most sensitive taxa being <1 mg Ni/L. Most of the reported chronic TECs for nano‐Ni^0^ in Table 1 exceeded 1 mg Ni/L, and all exceeded 0.1 mg Ni/L. The taxon‐specific geometric mean TECs ranged from 0.430 to <20 mg Ni/L, with geometric mean TECs for the 2 most sensitive taxa being <1 mg Ni/L.

All the reported acute L(E)C50s for nano‐NiO in Table 2 exceeded 1 mg Ni/L. The taxon‐specific geometric mean L(E)C50s ranged from 2.15 to >84.2 mg Ni/L, with geometric mean L(E)C50s for the 4 most sensitive taxa being <5 mg Ni/L. Most of the reported chronic TECs for nano‐NiO in Table 2 exceeded 1 mg Ni/L, and all exceeded 0.02 mg Ni/L. The taxon‐specific geometric mean TECs ranged from 0.0794 to 54.7 mg Ni/L, with geometric mean TECs for the 3 most sensitive taxa being ≤∼1 mg Ni/L.

Toxicity data were available for 2 other nano‐Ni‐oxides/hydroxides: nano‐Ni_2_O_3_ and nano‐Ni(OH)_2_. The L(E)C50 and L(E)C10 for *Danio rerio* embryos exposed to nano‐Ni_2_O_3_ was >35.5 mg Ni/L (Lin et al. 2013). No data for toxicity of nano‐Ni(OH)_2_ passed the quality screening.

#### Toxicity of Ni‐containing CENs.

Among the wide variety of the 34 Ni‐containing CENs that were used in aquatic toxicity tests, only 10 had toxicity data that passed the quality screening (Supplemental Data, Table S7).

Of the 18 acute L(E)C50s for Ni‐containing CENs that passed the quality screening, 22% were <1 mg Ni/L, but all exceeded 0.1 mg Ni/L (Supplemental Data, Table S7). The L(E)C50s ranged from <0.309 mg Ni/L (Ni‐CuO) to <20 000 mg Ni/L (cobalt [Co]‐doped and copper [Cu]‐doped α‐NiMoO_4_). That range of acute L(E)C50s for Ni‐containing CENs that passed the quality screening fell within and slightly exceeded the range published by the European Chemicals Agency (2019) for dissolved Ni (0.013–4970 mg Ni/L).

Of the 11 chronic TECs for Ni‐containing CENs that passed the quality screening, 73% were <1 mg Ni/L and 9% were <0.1 mg Ni/L (Supplemental Data, Table S7). The chronic TECs (as L[E]C10s or MICs) ranged from 0.0749 mg Ni/L (Ni_0.33_Co_0.33_Li_0.27_Mn_0.33_O_2_) to <5370 mg Ni/L (α‐NiMoO_4_). That range of chronic TECs for Ni‐containing CENs that passed the quality screening fell within and exceeded the range of chronic TECs published by the European Chemicals Agency (2019) for dissolved Ni (0.0114–52.3 mg Ni/L). Additional details are in the *Toxicity of Ni‐containing CENs* section in the Supplemental Data.

#### Nano‐Ni^0^ versus nano‐NiO toxicity.

**Figure 2:**
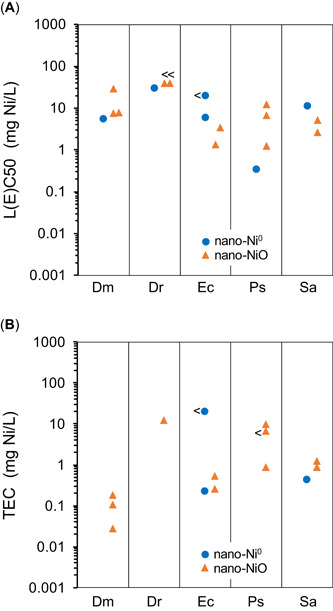
(**A** and **B**) Comparison of the toxicity of nanoparticulate elemental nickel (nano‐Ni^0^) and nanoparticulate nickel oxide (nano‐NiO) to aquatic organisms, with taxa for which both nano‐Ni^0^ and nano‐NiO data are available; only tests for which the quality‐screening rating exceeded “low” are included. Dm = *Daphnia magna* (water flea); Dr = *Danio rerio* (zebrafish); Ec = *Escherichia coli* (bacteria); L(E)C50 = median lethal (or effect) concentration; Ps = *Pseudokirchneriella subcapitata* (green microalga); Sa = *Staphylococcus aureus* (bacteria); TEC = threshold effect concentration (including 10% lethal [or effect] concentration, geometric mean of no‐observed‐effect concentration and lowest‐observed‐effect concentration, or geometric mean of minimum inhibitory concentration and next lower concentration [only for bacteria]); < = plotted concentration is a less‐than value. Individual toxicity test results are listed in the Supplemental Data, Tables S5 and S6.

Five taxa had acute L(E)C50s for both nano‐Ni^0^ and nano‐NiO that passed the quality screening (Table 3). In general, the ranges of L(E)C50s for nano‐Ni^0^ and nano‐NiO overlapped for each taxon (except possibly *Pseudokirchneriella subcapitata*), thus suggesting no major differences in toxicity between nano‐Ni^0^ and nano‐NiO (Figure 2A and Table 3). Two taxa had chronic TECs for both nano‐Ni^0^ and nano‐NiO that passed the quality screening (both bacteria), and the ranges of their chronic TECs for nano‐Ni^0^ and nano‐NiO were similar or overlapped (Figure 2B). However, strong conclusions about the relative toxicity of these 2 chemical forms of nano‐Ni cannot be reached because of the small number of species available for comparisons.

**Table 3:** Comparison of the toxicity to aquatic organisms, of nanoparticulate elemental nickel (nano‐Ni^0^) to the toxicity of nanoparticulate nickel oxide (nano‐NiO); only tests for which the quality‐screening rating exceeded “low” are included^a^

	Range of L(E)C50s (mg Ni/L)		Range of TECs (mg Ni/L)^b^	
Taxon and endpoints	Nano‐Ni^0^	Nano‐NiO	Nano‐Ni^0^	Nano‐NiO
*Danio rerio* (zebrafish) L(E)C50s: 48‐h survival or 67‐ to 120‐h hatching of embryos	>10–30.8 (*n* = 2)	<39.4 (*n* = 2)		
*Daphnia magna* (water flea) L(E)C50s: 48‐h survival of neonates	5.68 (*n* = 1)	7.67–29.0 (*n* = 3)		
*Pseudokirchneriella subcapitata* (green alga) L(E)C50s: 72‐ or 96‐h population growth	0.35 (*n* = 1)	1.26–12.5 (*n* = 3)		
*Escherichia coli* (bacteria) L(E)C50s: 6‐, 8‐, or 24‐h population growth TECs: 6‐, 8‐, or 24‐h population growth	6.03–<20 (*n* = 2)	1.34–3.46 (*n* = 2)	0.222–<20 (*n* = 2)	0.254–0.512 (*n* = 2)
*Staphylococcus aureus* (bacteria) L(E)C50s: 8‐ or 24‐h population growth TECs: 8‐ or 24‐h population growth	11.6 (*n* = 1)	2.68–5.18 (*n* = 2)	0.430 (*n* = 1)	0.874–1.21 (*n* = 2)

^a^ Individual toxicity‐test results are listed in the Supplemental Data, Tables S5 and S6.

^b^ TECs include: 10% lethal (or effect) concentration (L[E]C10); geometric mean of no‐observed‐effect concentration (NOEC) and lowest‐observed‐effect concentration (LOEC); and/or geometric mean of minimum inhibitory concentration (MIC) and next‐lower concentration.

L(E)C50 = median lethal (or effect) concentration; TEC = threshold effect concentration; *n* = number of L(E)C50s or TECs included in the range of values.

Therefore, based on the available data, nano‐Ni^0^ and nano‐NiO generally did not differ in their toxicity to aquatic organisms. This conclusion might be explained from the perspective of the chemical fate of the wetted surfaces of those NP (see the *Nano‐Ni* ^*0*^ *versus nano‐NiO toxicity* section in the Supplemental Data). However, nano‐Ni^0^ and nano‐NiO may differ in human health effects in nonaquatic exposure pathways (Buxton et al. 2019).

#### Size dependence of nano‐Ni toxicity.

Because nanomaterials are assumed to possess unique physical–chemical properties related to their small size, high surface area:volume ratio, and high surface energy (especially particles having diameters <20–30 nm; Auffan et al. 2009), thus leading to assumed high biological reactivity, it is logical to ask whether the toxicity of nano‐Ni increases as particle size decreases (and thus as surface energy increases) within the 1‐ to 100‐nm size range of nanomaterials.

Only 2 studies have directly compared the aquatic toxicity of different dry sizes of nano‐Ni in the same organism(s) (Ispas et al. 2009; Nogueira et al. 2015; Table 4). In general, the chronic NOECs, LOECs, and L(E)C10s and the acute L(E)C50s did not differ among the tested particle sizes by more than approximately 4‐fold in the 7 taxa × toxicity endpoint combinations. Furthermore, the toxicity of the smaller particles in each comparison was always less than or equal to the toxicity of the larger particles (i.e., the concentration of the smaller particles that caused a given effect percentage was always the same or higher than the concentration of the larger particles that caused the same effect percentage). Reason(s) have not been identified for these counterintuitive results, in which larger NPs tended to be more toxic than smaller NPs. Additional comparative toxicity tests would be needed to determine whether these trends are repeatable.

**Table 4:** Toxicity of various sizes of nanoparticulate nickel (nano‐Ni) to aquatic organisms, in paired tests conducted within the same study; only tests for which the quality‐screening rating exceeded “low” are included

Form of nano‐Ni	Taxon	Toxicity endpoint	Life stage at start of test	Particle size (nm)		Effect concentration (mg Ni/L)^a^				
				Nominal or measured dry size	Hydrodynamic size	NOEC	LOEC	L(E)C10	L(E)C50	Reference
Ni^0^	*Danio rerio* (freshwater; zebrafish)	96‐h survival	Embryo	28 63 112 540^b^	NR NR NR NR				328 361 221 115	Ispas et al. (2009)
NiO	*Artemia salina* (saltwater; brine shrimp)	24‐h survival	Larva	10–20 100	1243 1959				>15.8 >15.8	Nogueira et al. (2015)
NiO	*Brachionus plicatilis* (saltwater; rotifer)	48‐h survival	Neonate	10–20 100	1048 1144				>15.8 >15.8	Nogueira et al. (2015)
NiO	*Daphnia magna* (freshwater; water flea)	48‐h survival	Neonate	10–20 100	1400 1126				7.69 7.67	Nogueira et al. (2015)
		21‐d reproduction	Neonate	10–20 100	1400 1126	0.0867 0.0284	0.110 0.0355	0.0944 0.0268		Nogueira et al. (2015)
NiO	*Lemna minor* (freshwater; common duckweed)	7‐d growth	NR	10–20 100	990 1910				>15.8 3.90	Nogueira et al. (2015)
NiO	*Pseudokirchneriella subcapitata* (freshwater; green alga)	72‐h growth	NR	10–20 100	1266 2061	12.6 6.46	15.8 8.04	9.46 <6.46	12.5 6.68	Nogueira et al. (2015)

^a^ No entry indicates the value was not reported and insufficient information was provided to calculate the value.

^b^ Aggregates of nominal 60‐nm particles, with an average measured size of 540 nm.

L(E)C10 = 10% lethal (or effect) concentration; L(E)C50 = median lethal (or effect) concentration; LOEC = lowest‐observed‐effect concentration; Ni^0^ = elemental nickel; NiO = nickel oxide; NOEC = no‐observed‐effect concentration; NR = not reported.

#### Toxicity of nano‐Ni versus dissolved Ni.

Geometric mean acute L(E)C50s for nano‐Ni^0^, nano‐NiO, and other nano‐Ni‐oxides/hydroxides combined ranged from 0.35 to >84.2 mg Ni/L (Tables 1 and 2). For comparison, the European Chemicals Agency (2019) reported acute L(E)C50s for dissolved Ni ranging from 0.013 to 4970 mg Ni/L for freshwater and saltwater taxa. Geometric mean chronic TECs for nano‐Ni^0^, nano‐NiO, and other nano‐Ni‐oxides/hydroxides combined ranged from 0.430 to <20 mg Ni/L (Tables 1 and 2). For comparison, the European Chemicals Agency (2019) reported chronic NOECs or L(E)C10s for dissolved Ni ranging from 0.0014 to 52.3 mg Ni/L for freshwater and saltwater taxa.

Thus, except for 1 geometric mean chronic TEC, all reported geometric mean acute and chronic toxicities of nano‐Ni^0^, nano‐NiO, and other nano‐Ni‐oxides/hydroxides included in Tables 1 and 2 are within the range of reported toxicity of dissolved Ni. Similarly, no current evidence indicates that Ni‐containing CENs are more toxic than dissolved Ni (see *Toxicity of Ni‐containing CENs*). However, comparisons of nano‐Ni toxicity to the dissolved Ni toxicity results and comparisons of nano‐Ni toxicity among the taxa in Tables 1 and 2 should be interpreted with caution, because a wide variety of test durations, toxicity endpoints, and exposure‐water conditions are included in the nano‐Ni results. In addition, some of the geometric mean L(E)C50s and TECs are indefinite greater‐than (>) or less‐than (<) concentrations, making their actual values uncertain.

**Figure 3:**
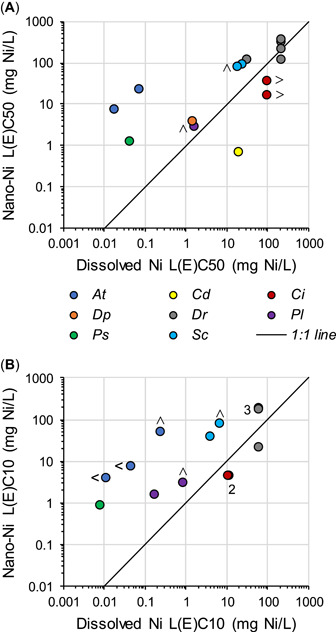
(**A** and **B**) Comparison of the toxicity of nanoparticulate nickel (nano‐Ni, as elemental nickel [Ni^0^] or nickel oxide [NiO]) to the toxicity of dissolved Ni in studies in which the 2 forms of Ni were tested under the same conditions; only tests for which the quality‐screening rating exceeded “low” are included. *At* = *Acartia tonsa*; Cd = *Ceriodaphnia dubia*; *Ci* = *Ciona intestinalis*; *Dp* = *Daphnia pulex*; *Dr* = *Danio rerio*; L(E)C50 = median lethal (or effect) concentration; L(E)C10 = 10% lethal (or effect) concentration; *Pl* = *Paracentrotus lividus*; *Ps* = *Pseudokirchneriella subcapitata*; *Sc* = *Saccharomyces cerevisiae*; < = plotted concentration is a less‐than value (in the direction the symbol points). Individual L(E)C50s and L(E)C10s are listed in Table 5.

Direct comparisons of the aquatic toxicity of nano‐Ni and dissolved Ni have been conducted with 8 taxa for which toxicity tests passed the quality screening (Table 5). For 5 of the 8 tested taxa, the L(E)C50s, L(E)C10s, LOECs, and NOECs for dissolved Ni were less than or equal to the corresponding lethal (or effect) concentrations for nano‐Ni^0^, when valid comparisons could be made (i.e., dissolved Ni was equally as toxic or more toxic than nano‐Ni on a mass‐concentration basis; Figure 3). In 1 of the 3 exceptions to nano‐Ni particles being less toxic than dissolved Ni, the particles were 540‐nm aggregates of 63‐nm nano‐Ni^0^ particles and thus might more appropriately be classified as colloidal Ni, despite being composed of NP‐sized subunits.

**Table 5:** Comparison of the toxicity of nanoparticulate nickel (nano‐Ni) with the toxicity of dissolved Ni salt to aquatic organisms, in paired tests conducted within the same study; only tests for which the quality‐screening rating exceeded “low” are included

Form of nano‐Ni	Taxon	Toxicity endpoint	Life stage at start of test	Form of Ni added to exposure water	Effect concentration (mg Ni/L)^a^				Reference
					NOEC	LOEC	L(E)C10	L(E)C50	
Ni^0^	*Danio rerio* (freshwater; zebrafish)	96‐h survival	Embryo	Nano‐Ni^0^: 28 nm Nano‐Ni^0^: 63 nm Nano‐Ni^0^: 112 nm Nano‐Ni^0^: 540 nm^b^ Dissolved Ni: salt NR				328 361 221 115 221	Ispas et al. (2009)
		48‐h survival	Larva	Nano‐Ni^0^: 5–20 nm Dissolved Ni: NiCl_2_				>10 >10	Griffitt et al. (2008)
		48‐h survival	Adult	Nano‐Ni^0^: 5‐20 nm Dissolved Ni: NiCl_2_				>10 >10	
Ni^0^	*Acartia tonsa* (saltwater; calanoid copepod)	48‐h hatching	Embryo	Nano‐Ni^0^: <100 nm Dissolved Ni: NiCl_2_				>50 >0.285	Zhou et al. (2016)
		48‐h hatching	Embryo (F1 gen.)^c^	Nano‐Ni^0^: <100 nm Dissolved Ni: NiCl_2_				14.7 >0.0453	
		48‐h survival	Embryo	Nano‐Ni^0^: <100 nm Dissolved Ni: NiCl_2_				22.1 0.0743	
		168‐h survival	Embryo	Nano‐Ni^0^: <100 nm Dissolved Ni: NiCl_2_	5 <0.0113	10 0.0113	3.77 <0.0113		
		96‐h reproduction	Adult	Nano‐Ni^0^: <100 nm Dissolved Ni: NiCl_2_	17 0.0453	>17 >0.0453	15.4 >0.0453		
Ni^0^	*Ceriodaphnia dubia* (freshwater; water flea)	48‐h survival	Neonate	Nano‐Ni^0^: 5–20 nm Dissolved Ni: NiCl_2_				0.674 19.6	Griffitt et al. (2008)
Ni^0^	*Daphnia pulex* (freshwater; water flea)	48‐h survival	Adult	Nano‐Ni^0^: 5‐20 nm Dissolved Ni: NiCl_2_				3.89 1.48	Griffitt et al. (2008)
Ni^0^	*Ciona intestinalis* (saltwater; sea squirt)	50‐min fertilization	Sperm	Nano‐Ni^0^: <100 nm Dissolved Ni: NiCl_2_				36.1 >100	Gallo et al. (2016)
		24‐h morphology	Larva	Nano‐Ni^0^: <100 nm Dissolved Ni: NiCl_2_				16.8 >100	
Ni^0^	*Paracentrotus lividus* (saltwater; purple sea urchin)	48‐h morphology	Embryo	Nano‐Ni^0^: 48 nm Dissolved Ni: NiCl_2_				>3 1.53	Kanold et al. (2016)
		48‐h growth	Embryo	Nano‐Ni^0^: 48 nm Dissolved Ni: NiCl_2_				>3 >0.781	
NiO	*Pseudokirchneriella subcapitata* (freshwater; green alga)	72‐h population growth	Exponential growth	Nano‐NiO: <50 nm Dissolved Ni: NiCl_2_			0.867 0.0080	1.26 0.042	Sousa et al. (2018b)
NiO	*Saccharomyces cerevisiae* (freshwater; yeast)	6‐h cell viability in MES buffer^d^	Exponential growth	Nano‐NiO: <50 nm Dissolved Ni: NiCl_2_			38.8 3.9	92.7 24	Sousa et al. (2018a)
		24‐h population growth in YEP broth^e^	Exponential growth	Nano‐NiO: <50 nm Dissolved Ni: NiCl_2_			>78.8 6.6	>78.8 18.3	

^a^ No entry indicates the value was not reported and insufficient information was provided to calculate the value.

^b^ Aggregates of nominal 60‐nm particles, with an average measured size of 540 nm.

^c^ Embryos from Ni‐exposed adults instead of from naïve adults.

^d^ MES buffer = 10 mmol/L of 2‐(N‐morpholino)ethanesulfonic acid with 20 g glucose/L, adjusted to pH 6.0.

^e^ YEP broth = yeast extract‐peptone‐dextrose broth adjusted to pH 6.0.

L(E)C10 = 10% lethal (or effect) concentration; L(E)C50 = median lethal (or effect) concentration; LOEC = lowest‐observed‐effect concentration; Ni^0^ = elemental nickel; NiO = nickel oxide; NOEC = no‐observed‐effect concentration; NR = not reported.

Therefore, there is conflicting evidence to decide whether nano‐Ni is more toxic than dissolved Ni. Although the weight of evidence suggests that dissolved Ni is equally as toxic or more toxic than nano‐Ni^0^ and nano‐NiO on a mass‐concentration basis, the 2 opposite results suggest caution in overgeneralizing that conclusion. This conclusion is consistent with Shaw and Handy (2011), who concluded that dissolved metal salts might generally be more acutely toxic to fish than are their corresponding nanometals. However, opposite results sometimes occur among different taxa or among different life stages within a taxon. We suggest that different mechanisms of the toxicity of nano‐Ni among various organisms might lead to a gradient of comparative toxicities, with nano‐Ni being less toxic, equally as toxic, or more toxic than dissolved Ni depending on the taxon and life stage under consideration (perhaps determined by differences or similarities in the routes of exposure). However, at least some of the conflicting evidence might also be due to variations in test procedures (e.g., particle preparation [sonication/dispersion], test methods, exposure duration) and/or to type I or type II statistical errors (i.e., false positives or false negatives resulting from random variation in responses by the test organisms). Additional comparative toxicity studies would be needed to allow more definitive conclusions.

### Mechanisms of nano‐Ni toxicity

#### Molecular, physiological, and structural responses.

An important consideration for toxicity of NPs is their interaction with membranes, cell walls, and surfaces of tissues/organs (Handy and Al‐Bairuty 2019; Jimeno‐Romero et al. 2019; Sendra et al. 2019). In general, nano‐Ni particles can adsorb/adhere to the external surface of most organisms (e.g., by simple attraction to a negatively charged surface) and chemically react with the membranes, cell walls, and tissue/organ surfaces, thereby potentially leading to impairment of cell function and possibly death (Sendra et al. 2019).

In addition, nano‐Ni can cause chemical damage. Nano‐Ni induces production or release of ROS inside of or external to organisms (Han et al. 2012; Zhang et al. 2013; Fu et al. 2014; Oukarroum et al. 2015, 2017; Jeyaraj Pandian et al. 2016; Peng et al. 2018; Sousa et al. 2018a, 2018b, 2018c). This is a common mechanism of many metal‐containing NPs, leading to oxidative stress (Canesi et al. 2019). The oxidative stress manifests as a cascade of well‐demonstrated effects (Supplemental Data, Table S9), although some individual processes listed in that table might also be caused by 1 or more other stressors. In fish, gills are especially susceptible to oxidative stress (Jayaseelan et al. 2014). However, caution should be exercised when reading the literature, because many authors invoke oxidative stress mechanisms as potential explanations for observed nano‐Ni toxicity, but without specific evidence from their studies.

Physically, nano‐Ni can damage skin, tissue/organ surfaces, cell walls, and internal membranes, presumably through production of ROS that disrupts structural integrity (Gallo et al. 2016; Oukarroum et al. 2017; Peng et al. 2018) and leads to leakage of intracellular proteins across membranes into the extracellular medium (Jeyaraj Pandian et al. 2016). Exposure to nano‐Ni can also lead to cell enlargement and aberrant cell morphology (Oukarroum et al. 2017; Sousa et al. 2018b), and it has even been reported to cause skeletal damage such as scoliosis in fish (Morgaleva et al. 2017). In addition, nano‐Ni particles can partially to totally dissolve in exposure water or inside an organism, releasing Ni^2+^ ions that can cause ionoregulatory, oxidative, or respiratory stress associated with dissolved Ni toxicity (see review by Brix et al. 2017).

**Figure 4:**
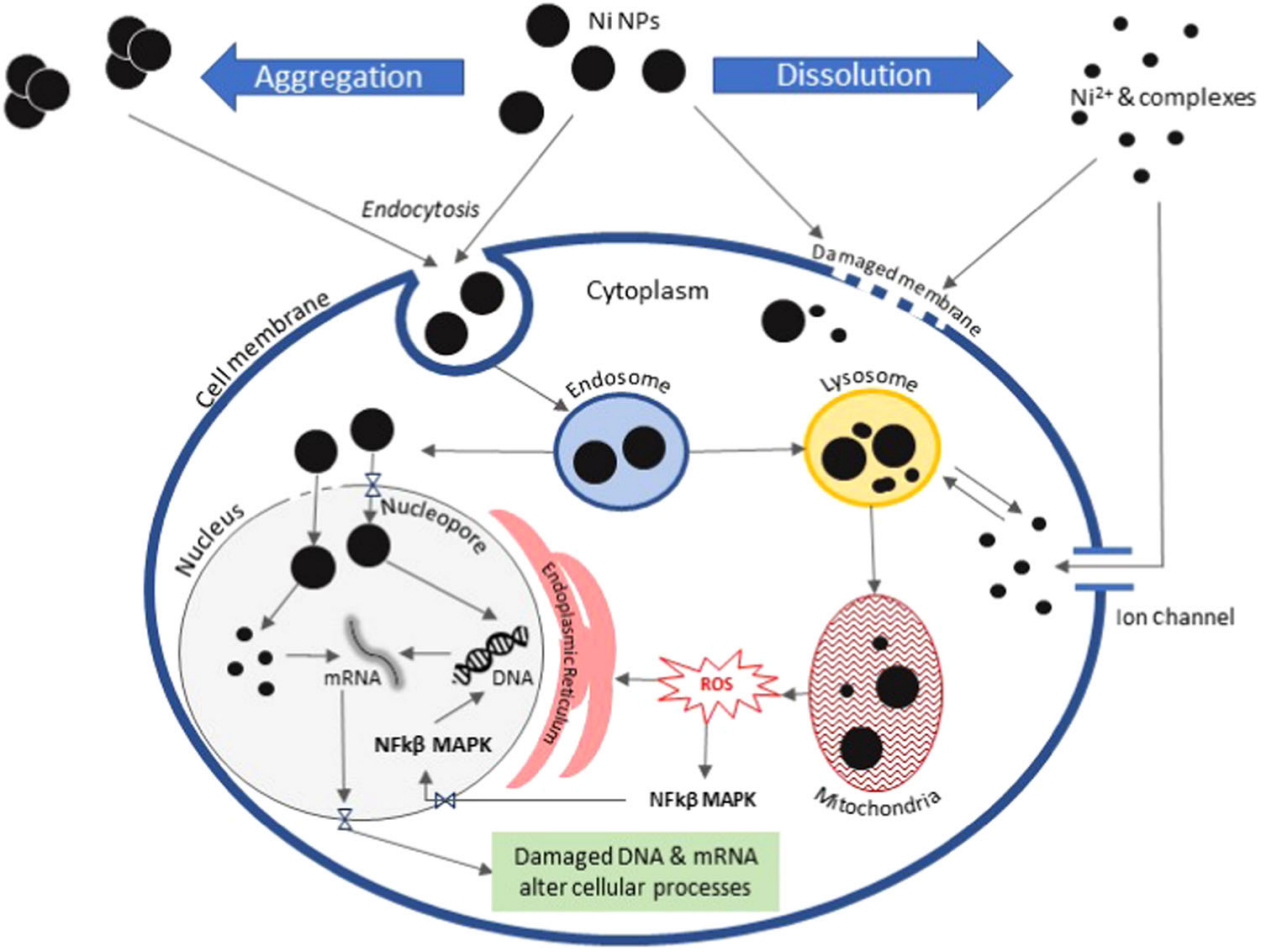
Possible sequences in the cascade of external interaction, entry into, and internal interaction of nickel nanoparticles (Ni NPs) and released nickel ions (Ni^2+^) with cells (adapted from Biswas et al. 2018). mRNA = messenger ribonucleic acid; MAPK = mitogen‐activated protein kinase (part of the molecular response to a variety of stresses, including oxidative stress, in eukaryotes); NFkβ = nuclear factor kappa B (1 of several genetic transcription factors); ROS = reactive oxygen species.

Some of the possible pathways for external interactions, entry into, and internal interactions of NPs with cells are illustrated in Figure 4. In addition to nano‐Ni particles and aggregates adsorbing/adhering to external surfaces of organisms (Gong et al. 2016; Sousa et al. 2018a, 2018b), nano‐Ni particles sometimes cross membranes and accumulate in intact cells (Shaw and Handy 2011; Oukarroum et al. 2017), although not always (e.g., Sousa et al. 2018a). Even the simple physical burden of nano‐Ni particles attached to an organism might be detrimental at high exposure concentrations that result in particles covering the entire surface of the organism (e.g., *Daphnia magna*; Figure 3F in Gong et al. 2016). Ingestion of particles can lead to accumulation of nano‐Ni in the gastrointestinal tract (Ispas et al. 2009; Özel et al. 2014; Ates et al. 2016; Figures 3B–D in Gong et al. 2016), leading to potential intestinal histopathologies (Ispas et al. 2009), disruption of gut physiology (e.g., Özel et al. 2014), and alteration of the microbial community in the intestine (Bagirov et al. 2019). Internalized nano‐Ni particles might damage organelles or organs (Ispas et al. 2009; Jayaseelan et al. 2014; Nazdar et al. 2018), either by direct contact or by dissolution of the NPs (e.g., inside acidic lysosomes) and subsequent damage by the dissolved Ni (whereby the NPs act as a “delivery vehicle” for Ni^2+^ ions, as suggested by Shaw and Handy 2011).

However, the results of the physiology, histopathology, intestinal microbiome, and accumulation studies discussed in this section should be placed in perspective, because all were conducted at nano‐Ni exposure concentrations ≥0.01 mg/L (Table 6). Those nano‐Ni exposure concentrations likely greatly exceed current nano‐Ni concentrations in most surface waters, because the total Ni concentrations in surface waters (which include “true” dissolved, nano, colloidal, and particulate forms of Ni) usually are <∼0.01 mg Ni/L (e.g., the range of “reasonable worst case predicted environmental concentrations” across 8 European countries is 0.0015–0.012 mg Ni/L; Table 3.1.10‐22 in European Commission 2008). We are unaware of any direct measurements of nano‐Ni concentrations in surface waters that could narrow the range of realistic exposure concentrations.

**Table 6:** Waterborne exposure concentrations of nanoparticulate nickel (nano‐Ni) that were used in physiology, histopathology, intestinal microbiome, and particle‐accumulation studies

Form of nano‐Ni^a^	Concentration range (mg Ni/L)^b^	Type of study	Reference
Ni^0^	0.015–20	Histopathology	Morgaleva et al. (2017)
	0.01–1	Intestinal microbiome	Bagirov et al. (2019)
	0.1–10	Histopathology, physiology	Jayaseelan et al. (2014)
	1–100	Histopathology, physiology	Gallo et al. (2016)
	1–100	Accumulation, physiology	Özel et al. (2014)
	10	Physiology	Zhang et al. (2013)
	10–1000	Accumulation, histopathology	Ispas et al. (2009)
	25–100	Physiology	Jeyaraj Pandian et al. (2016)
NiO	0.01–1	Intestinal microbiome	Bagirov et al. (2019)
	0.08–40	Accumulation	Ates et al. (2016)
	0.08–80	Accumulation, histopathology, physiology	Oukarroum et al. (2017)
	0.8–4	Physiology	Han et al. (2012)
	0.8–800	Physiology	Oukarroum et al. (2015)
	0.9–3	Accumulation, histopathology, physiology	Sousa et al. (2018b)
	4–60	Accumulation	Gong et al. (2016)
	40–80	Physiology	Sousa et al. (2018c)
	80	Accumulation, physiology	Sousa et al. (2018a)
	160	Histopathology, physiology	Peng et al. (2018)

^a^ Ni^0^ = elemental nickel; NiO = nickel oxide.

^b^ All reported concentrations were nominal, not measured.

Much of the mechanistic knowledge about nano‐Ni toxicity emanates from studies with unicellular organisms. Instead of being bactericidal or algicidal (i.e., killing bacteria or microalgae), relatively low to intermediate concentrations of nano‐Ni appear to more commonly be bacteriostatic or algistatic (i.e., nano‐Ni slows or halts the cell cycle, thus slowing or halting cell division and population growth; Argueta‐Figueroa et al. 2014; Sousa et al. 2018b). However, high concentrations of nano‐Ni can cause cell death (e.g., see the sequence of events in the yeast *Saccharomyces cerevisiae* in Figure 6 in Sousa et al. 2019).

We conclude that nano‐Ni triggers the same general suite of molecular, physiological, and structural toxicity responses in aquatic organisms as are triggered by other metal‐based nanomaterials (e.g., reviews by Handy et al. 2008b; Klaine et al. 2008; Shaw and Handy 2011; Fu et al. 2014; Biswas et al. 2018). However, specific toxic effects in response to dissolved Ni released from nano‐Ni particles might differ from specific toxic effects in response to dissolved metals released from other metal‐containing NPs (e.g., see review by Brix et al. 2017).

#### Differences among species and life stages.

Sensitivity to nano‐Ni exposure spans several orders of magnitude among taxa (see ranges of acute L[E]C50s and chronic TECs for nano‐Ni^0^ and nano‐NiO in Tables 1 and 2), similar to the magnitudes of variation for dissolved Ni (European Chemicals Agency 2019). Filter‐feeding invertebrates and microalgae appear to generally be among the most sensitive taxonomic groups tested; however, some bacteria also have low L(E)C50s or TECs. Thus, the relatively early conclusion by Griffitt et al. (2008) that daphnids and algae appeared to be the most sensitive taxa to nano‐Ni is partially supported by the present meta‐analysis. Continual ingestion of nano‐Ni particles and agglomerates while essentially feeding constantly (and the subsequent accumulation of those particles in the gut where they can damage the intestine, be internalized via endocytosis, or dissolve) might explain the relatively high sensitivity of filter feeders to nano‐Ni. Adsorption/adhesion of nano‐Ni particles and agglomerates onto the cell surface and/or internalization of the particles might explain the relatively high sensitivity of algae and bacteria. However, it is interesting that another unicellular taxon (the yeast *S. cerevisiae*) appears to be relatively insensitive to nano‐Ni (Table 2).

Within a given taxon, differences might occur among life stages in their responses and sensitivity to nano‐Ni. Only 2 studies have addressed such developmental changes, both using zebrafish. Özel et al. (2014) reported that changes in the concentration of serotonin secreted in the intestine of *D. rerio* embryos varied with the exposure period, the concentration of nano‐Ni^0^, and the developmental stage of the embryo. Among other functions, serotonin stimulates intestinal motility, which is important for digestion of food. Before hatching, the chorion (a membrane surrounding the embryo) appeared to protect the zebrafish embryos from NPs that accumulated on but could not pass through it, leaving dissolved Ni mainly responsible for the observed impairment of hatching. However, after the chorion was shed at hatching, NPs could enter the developing intestine as it opened to the external environment, and the nano‐Ni could then alter serotonin production. Similarly, Peng et al. (2018) reported that hatching interference in *D. rerio* was most likely caused by dissolution of Ni from nano‐NiO, followed by production of ROS during the embryonic life stage, but additional biotic ROS generation and larval skin damage were attributable to the NPs during the subsequent larval (posthatch) life stage. Therefore, identification of the most sensitive life stages in a variety of taxa might improve the development of reliable nano‐Ni toxicity models.

#### Source of nano‐Ni toxicity: Nano‐Ni particles or dissolved Ni?

Because 1) nano‐Ni particles can at least partially dissolve in water, and 2) both nano‐Ni particles and dissolved Ni can cause toxicity in aquatic organisms (see *Molecular, physiological, and structural responses*), researchers sometimes attempt to determine whether nano‐Ni toxicity is caused directly by the nano‐Ni particles, or indirectly by the dissolved Ni released from the particles, or by both. Although the particles are the ultimate source of the toxicity regardless of the proximate cause, it would be helpful to distinguish among the proximate causes of toxicity if reliable models of nano‐Ni toxicity are to be developed.

Publications that quantified the dissolution of nano‐Ni particles in toxicity studies and passed the quality screening are listed in Table 7. Six of the 14 entries presented evidence that dissolved Ni released from nano‐Ni particles contributed a majority of the observed toxicity (i.e., the nano‐Ni particles contributed relatively little directly to the toxicity, for both nano‐Ni^0^ and nano‐NiO). The remainder of the entries either validly concluded relatively minor contributions of the dissolved Ni to toxicity, did not have sufficient evidence to reach a valid conclusion, did not reach a conclusion, or did not test for toxicity in that water (in the latter situation, always in deionized water exposures run for a particle‐size/agglomeration comparison with the exposure used in a separate test).

**Table 7:** Percentages of nanoparticulate nickel (nano‐Ni) that dissolved in various exposure waters, and conclusions about the contributions of the dissolved Ni to the observed toxicity; only tests for which the quality‐screening rating exceeded “low” are included^a^

Type of water	Form of nano‐Ni	Dissolution of nano‐Ni	Duration (d)	Particle size (nm)		Taxon tested for toxicity or physiological response	Inferred contribution of dissolved Ni to observed response^b^	Reference	Comments
				Dry	Hydrodynamic				
SW	NiO	0.02–0.2%	NR	20	NR	*Chlorella vulgaris* (green alga; brackish water strain)	Possible small contribution to inhibition of population growth	Gong et al. (2011)	Exposure in f/2 medium (enriched sea water). Chemistry: 23 °C. Percentage of dissolution generally higher at higher nano‐NiO concentrations. Nano‐NiO partially reduced to nano‐Ni^0^ (12% at 24 h to 19% at 120 h).
	Ni^0^	∼1–2%^c^	0–2	<100	∼100–800	*Acartia tonsa* (copepod)	Main cause of mortality	Zhou et al. (2016)	Exposure in 0.22‐μm filtered seawater. Chemistry: 20 °C, pH 8.2, 30‰ salinity. Sonication did not alter dissolution or hydrodynamic size of nano‐Ni^0^ particles. Hydrodynamic size increased as nano‐Ni^0^ concentration increased. In deionized water, hydrodynamic size was ∼100 nm. Intensity of darkness of particles decreased after 48 h in sea water, suggesting oxidation of the Ni^0^ core. At 50% mortality, dissolved Ni concentration in this nano‐Ni^0^ test was extrapolated as 0.275 mg Ni/L, 3.7‐fold higher than dissolved Ni EC50 of 0.0743 mg Ni/L in a companion NiCl_2_ toxicity test conducted in the same exposure‐water chemistry in the same laboratory.
	Ni^0^	∼3% ∼8%	2 46	48	NR	*Paracentrotus lividus* (sea urchin)	Interfered with developmental process, but unquantified^d^	Kanold et al. (2016)	Exposure in artificial sea water, with no organics added. Chemistry: 18–19 °C, pH 8.0, salinity = 35‰.
FW	Ni^0^	∼0.05–0.8%	0–2	20	∼600– ∼1400 or ∼3000^e^	*Escherichia coli* (bacteria)	NR	Zhang et al. (2013)	Exposure in deionized water. Chemistry: 22 °C. ∼0.05% of nano‐Ni^0^ dissolved in the dark; ∼0.8% of nano‐Ni^0^ dissolved under 0.78 mW/cm^2^ UV illumination.
	Ni^0^	∼2%	4	28, 63, 112, 540^f^	NR	*Danio rerio* (zebrafish)	2.4–3.8% contribution to embryo mortality^g^	Ispas et al. (2009)	Exposure in E3 embryo medium (moderately saline freshwater). Chemistry: 28.5 °C, 5 mM NaCl, 0.17 mM KCl, 0.33 mM CaCl_2_, 0.33 mM MgSO_4_. Approximately the same percentage of dissolution for all 4 sizes of particles.
	NiO	∼2–3%	24	<50	344 (0 h); 1105 (24 h)	*Saccharomyces cerevisiae* (yeast)	Contributes to but does not explain all (or most) of decreased cell viability	Sousa et al. (2018a)	Exposure in YEP (yeast extract–peptone–dextrose) broth. Chemistry: pH 4.7–6.0. Peptides in YEP broth might have coated nano‐NiO particles, decreasing their toxicity.
FW	NiO	∼4–6%	24	<50	∼1200 (0 h); ∼5000 (24 h)	*Saccharomyces cerevisiae* (yeast)	Contribution to but not all (or not most) of decreased cell viability	Sousa et al. (2018a)	Exposure in MES [2‐(N‐morpholino) ethane‐sulfonic acid] buffer, with 20 g glucose/L. Chemistry: pH 5.8–6.0.
	NiO	∼6%	6 and 24	<50	324	NT in this water	NA	Sousa et al. (2018a)	Exposure in deionized water. Chemistry not reported.
	NiO	∼8‐9%	1–2	∼4	∼640	*Danio rerio* (zebrafish)	Major contribution to decreased embryo hatching^h^	Peng et al. (2018)	Exposure in Holtfreter's medium (relatively saline fresh water containing 59 mM NaCl). Chemistry: 28.5 °C. Used DTPA (diethylene triamine pentaacetic acid) to chelate dissolved Ni and eliminate hatching impairment, thus implicating dissolved Ni as the cause of most or all of the observed toxicity.
FW	Ni^0^	∼9%	46	48	NR	NT in this water	NA	Kanold et al. (2016)	Exposure in deionized water. Chemistry not reported.
	NiO	∼12–15%	2	∼13	∼280	*Danio rerio* (zebrafish)	Major contribution to decreased embryo hatching^h^	Lin et al. (2013)	Exposure in Holtfreter's medium (relatively saline freshwater containing 59 mM NaCl) with alginate (100 mg/L) added. Chemistry: 28.5 °C, pH 7.0. Carboxylate moiety on alginate might have complexed Ni^2+^, thus increasing amount of Ni dissolved from nano‐NiO. Used DTPA (diethylene triamine pentaacetic acid) to chelate dissolved Ni and eliminate hatching impairment, thus implicating dissolved Ni as the cause of most or all of the observed toxicity.
	NiO	∼6–16%	0.04–2	40	247	*Danio rerio* (zebrafish)	Major contribution to decreased embryo hatching^h^	Lin et al. (2011)	Exposure in Holtfreter's medium (relatively saline freshwater) with alginate (100 mg/L) added. Chemistry: 28.5 °C, pH 7.0, 60 mM NaCl, 400 mM NaHCO_3_, 0.6 mM KCl, 100 mM MgSO_4_, 10 mM CaCl_2_. Carboxylate moiety on alginate might have complexed Ni^2+^, thus increasing amount of Ni dissolved from nano‐NiO. Used DTPA (diethylene triamine pentaacetic acid) to chelate dissolved Ni and eliminate hatching impairment, thus implicating dissolved Ni as the cause of most or all of the observed toxicity.
FW	NiO	∼6–20%	3	<50	∼800 (0 h); ∼3400 (72 h)	*Pseudokirchneriella subcapitata* (green alga)	Explains most of decreased population growth	Sousa et al. (2018b)	Exposure in OECD algal growth medium. Chemistry: 25 °C, pH 7.4–7.7. Presence of algal cells enhanced nano‐NiO agglomeration. Percentage of dissolution decreased as nano‐NiO concentration was increased. At 50% mortality, dissolved Ni concentration in this nano‐NiO test was 0.2 mg Ni/L, ∼5‐fold higher than dissolved Ni EC50 of 0.042 mg Ni/L in a companion NiCl_2_ toxicity test conducted in the same exposure‐water chemistry in the same laboratory.
	Ni^0^	∼28%	2	6	∼300 (∼30–1000)	*Daphnia pulex* (cladoceran)	Possibly a majority contribution to lethality (dissolved Ni = 70% of EC50)	Griffitt et al. (2008)	Exposure in moderately hard dechlorinated tap water. Chemistry: pH 8.2, hardness = 142 mg/L as CaCO_3_, and conductivity = 395 μS/cm. Organic matter concentration not reported. Percent dissolution of nano‐Ni^0^ calculated from results plotted in the authors' Figures 2 and 3c, not from their Table 1. Dissolved Ni concentration in this nano‐Ni^0^ test was 1 mg Ni/L, ∼70% of the dissolved Ni EC50 of 1.48 mg Ni/L in a companion NiCl_2_ toxicity test conducted in the same exposure‐water chemistry in the same laboratory.

^a^ Results from Nogueira et al. (2015) are not included because their reported dissolution of nano‐NiO in a wide variety of water types (∼60–70%) is suspect, because only particle settling (not filtration or centrifugation) was used to separate dissolved and particulate Ni in the water columns.

^b^ Based on conclusions by authors, unless otherwise noted.

^c^ Although Zhou et al. (2016) reported 24–69% dissolved Ni (as a percentage of total Ni remaining in the water column after 55‐μ water column after 55‐percentage of total Ni rem∼1–2% dissolution based on nominal nano‐Ni^0^ concentrations.

^d^ Conclusive evidence not provided to support the author's assertion, because paired tests with dissolved Ni were not conducted.

^e^ Hydrodynamic sizes: ∼600 nm at 0 h, ∼1,400 nm at 48 h in the dark, and ∼3,000 nm at 48 h under UV illumination.

^f^ Size of aggregates of 63‐nm particles.

^g^ Range of percentages reported by authors, but method of calculating the percentages not explained.

^h^ Conclusion based on reversal of hatching impairment when DTPA (diethylenetriamine pentaacetic acid, a metal‐chelator) was added to the exposure water, thus decreasing bioavailability of dissolved Ni; however, that does not preclude possible modification of surface of nano‐NiO particles by DTPA, too.

EC50 = median lethal effect concentration; FW = freshwater‐based; NA = not applicable; Ni^0^ = elemental nickel; NiO = nickel oxide; NR = not reported; NT = not tested; SW = saltwater‐based; OECD = Organisation for Economic Co‐operation and Development.

One of the 6 entries in Table 7 for which dissolved Ni appeared to contribute considerably to the observed toxicity had a relatively low percentage of nano‐Ni dissolution (mortality of *Acartia tonsa* nauplii in ∼1–2% dissolution of nano‐Ni^0^ in sea water; Zhou et al. 2016), but the reported dissolved Ni concentration was 3.7‐fold higher than the dissolved Ni EC50 in a companion 48‐h NiCl_2_ lethality test (Table 5). The other 5 entries in Table 7 for which dissolved Ni appeared to contribute considerably to the observed toxicity had relatively high percentages of nano‐Ni dissolution (∼8–26% dissolution of nano‐Ni^0^ or nano‐NiO in fresh water; Griffitt et al. 2008; Lin et al. 2011, 2013; Peng et al. 2018; Sousa et al. 2018b). Peng et al. (2018) and Lin et al. (2011, 2013) used diethylene triamine pentaacetic acid (DTPA) to chelate dissolved Ni and eliminate hatching impairment in *D. rerio*, thus implicating dissolved Ni as the cause of most or all of the observed toxicity. Although Griffitt et al. (2008) reported only 3.7% dissolution of nano‐Ni^0^ in their Table 1, that dissolution was measured in the absence of *Daphnia pulex*; the more appropriate value of 28% dissolution is listed in Table 7, which we calculated from the measured dissolved Ni and particulate nano‐Ni^0^ concentrations in the presence of *D. pulex* exposed to nano‐Ni^0^ at the experimentally determined EC50. That is, we calculated that 28% dissolution from the EC50 for dissolved Ni in that nano‐Ni toxicity test (1.48 mg Ni/L) divided by the sum of the EC50 values for dissolved Ni and nano‐Ni^0^ in the same test (1.48 + 3.89 mg Ni/L; Table 3 in Griffitt et al. 2008).

Therefore, given the available results, percentage dissolution of nano‐Ni particles alone is not a good universal predictor of the relative contributions of nano‐Ni particles and dissolved Ni to toxicity in nano‐Ni exposures. However, the available results are consistent with little or no contribution of dissolved Ni to the toxicity when percentage dissolution of the nano‐Ni particles is very low (e.g., <1%); however, the toxic contribution of dissolved Ni that releases from nano‐Ni particles can be relatively high when the percentage dissolution is high.

Water chemistry appears to at least partially control the dissolution of nano‐Ni particles, thus at least partially controlling the contribution of dissolved Ni to nano‐Ni toxicity. Based on the available information in Table 7, nano‐Ni particles are generally less soluble in seawater than in freshwater. The chemical form of the nano‐Ni does not appear to affect dissolution, because results for nano‐Ni^0^ and nano‐NiO were distributed throughout the ranges of dissolution percentages for freshwater and saltwater exposures.

The presence of organic matter in the exposure water might be a major factor affecting dissolution of nano‐Ni particles. However, the strength of this conclusion is limited because most of the publications listed in Table 7 did not report sufficient water chemistry information to allow a more robust analysis of the potential complexation of Ni^2+^ by organic matter and thus the potential contribution of bioavailable Ni to the toxicity. Knowledge of dissolved Ni concentration alone is not sufficient to predict the toxicity of dissolved Ni, because several water chemistry parameters (e.g., temperature, pH, alkalinity, and concentrations of major ions and dissolved organic carbon [DOC]) predictably modify the toxicity of dissolved Ni (Kozlova et al. 2009; Nys et al. 2016). It bears emphasizing that water chemistry matters in metal toxicity tests (Meyer et al. 2012), whether for dissolved or nanoparticulate metals. In addition, the susceptibility to toxicity caused by dissolved Ni released from nano‐Ni particles might be taxon dependent. This highlights the need for researchers to analyze and report a full suite of the water chemistry parameters in nano‐Ni and other nano‐metal exposures that will allow more robust conclusions about the potential contribution of dissolved Ni and other metals to observed toxicity. Such information and knowledge of taxon‐specific sensitivity to dissolved Ni and nano‐Ni particles could help to produce reliable models of nano‐Ni toxicity.

Additional information about nano‐Ni dissolution, toxicity in exposure waters containing nano‐Ni and dissolved Ni, and physical and chemical behaviors contributing to major similarities and differences between nano‐Ni and dissolved Ni are in the *Source of nano‐Ni toxicity: Nano‐Ni particles or dissolved Ni?* Section in the Supplemental Data.

#### Ultraviolet irradiation.

Light probably plays an important role in the toxicity of nano‐Ni. Zhang et al. (2013), Rajan et al. (2017), and Ezhilarasi et al. (2018) proposed physical mechanisms by which ultraviolet (UV) radiation can excite electrons on a NP surface, and those electrons can then react with O_2_ and hydroxyl ions (OH^–^) to generate several ROS (i.e., superoxide anion [O_2_^–^], hydrogen peroxide [H_2_O_2_], hydroxyl radicals [^•^OH], and singlet oxygen [^1^O_2_]), a principal cause of nano‐Ni toxicity (see *Molecular, physiological, and structural responses*). In addition, Zhang et al. (2013) reported that >10‐fold more Ni dissolved from nano‐Ni^0^ during up to 48 h of UV irradiation (365‐nm wavelength at 0.78 mW/cm^2^) than during the same amount of time in the dark, thus providing a 2‐pronged pathway for nano‐Ni toxicity: oxidative stress from production of ROS, and a more general toxicity from production of dissolved Ni. However, we are unaware of any published comparison of the acute or chronic toxicity of nano‐Ni in the presence and absence of light.

#### Sonication.

In 30% of the nano‐Ni toxicity tests, the researchers stated that they sonicated either their stock solutions of nano‐Ni or the actual exposure waters before toxicity testing, to ensure the exposures were not started with agglomerated NPs. Thus, in 70% of the toxicity tests, the nano‐Ni stock solutions or exposure waters were not sonicated or the authors did not indicate whether they used sonication. In the only study that investigated whether sonication affects the toxicity of nano‐Ni to aquatic organisms, Zhou et al. (2016) concluded that under their experimental conditions, the toxicity of presonicated treatments to *A. tonsa* did not differ from the toxicity of nonsonicated treatments. In part, this might have occurred because the dimensions and amount of dissolution of the wet nano‐Ni^0^ particles did not differ between the treatments after agglomeration in the exposure water, despite the pretreatment. Thus, because NPs naturally agglomerate, presonication might not be representative of exposure conditions in natural waters. Instead, sonication could lead to a worst‐case scenario for testing, which might be desirable depending on the testing purpose. Additional toxicity studies would be needed to more conclusively test the almost universal perception that presonication of exposure waters containing nano‐Ni is necessary.

### Uncertainties

For a given type of nano‐Ni, lack of standardization of toxicity test methods might be a major contributor to the ranges of acute and chronic toxicity results within a specified taxon. Moreover, variation in the amount of Ni released from the nano‐Ni particles during the toxicity test might also contribute within‐taxon variation in toxicity results (see *Source of nano‐Ni toxicity: Nano‐Ni particles or dissolved Ni?*). In addition, factors that modify the toxicity of nano‐Ni particles and dissolved Ni need to be considered. Incorporation of toxicity‐modifying factors into the analysis of nano‐Ni toxicity is hampered because most authors have not reported full water chemistry data sufficient for use in bioavailability models of dissolved Ni toxicity to aquatic organisms. Similarly, the general lack of reporting of the type, duration, and intensity of lighting during nano‐Ni tests and the general lack of measurements of concentrations of particulate nano‐Ni and dissolved Ni in exposure waters hampers the advancement of models of ROS generation and nano‐Ni dissolution (see *Nano‐Ni exposure during toxicity tests*).

Across all the various chemical forms of nano‐Ni reviewed in the present meta‐analysis, 83% received an overall quality rating of “low” (Supplemental Data Excel File). Thus, using only the other 17% of the toxicity tests in our detailed analysis engenders some uncertainty, because otherwise reliable L(E)C50s and TECs might not be included in the detailed analyses due to inadequate reporting of test methods, test results, and physical–chemical characteristics of the nano‐Ni particles and exposure waters.

#### Uncertainties in nano‐Ni toxicity datasets.

The nano‐Ni toxicity database is dominated by tests with nano‐Ni^0^ and nano‐NiO, with relatively little information about other Ni‐oxides, Ni‐hydroxides, and each of the variety of Ni‐containing CENs listed in the Supplemental Data, Table S7. In addition, we are unaware of any toxicity tests with a variety of other chemical forms of nano‐Ni (e.g., acetates, carbides, carbonates, nitrates, phosphides, sulfates, sulfides; alloys with Co, chromium, Cu, lead, platinum, or titanium; carbon‐coated Ni). Testing with previously untested chemical forms of nano‐Ni and additional testing with the seldom tested forms could help to decrease uncertainties around the general toxicity of nano‐Ni.

Although a moderately diverse set of aquatic organisms populates the nano‐Ni toxicity database (i.e., fish, invertebrates, plants, a macroalga, microalgae, a yeast, fungi, bacteria, and a virus), amphibians have only been tested with 2, relatively atypical, forms of nano‐Ni (Ni^0^‐coated and NiO‐coated nanoparticulate γ‐Al_2_O_3_; Svartz et al. 2017, 2019). Some of the more commonly tested freshwater and saltwater fish (e.g., fathead minnow [*Pimephales promelas*], sheepshead minnow [*Cyprinodon variegatus*], rainbow trout [*Oncorhynchus mykiss*] and filter‐feeding saltwater benthic invertebrates [blue mussel *Mytilus edulis*]) are also absent from the dataset. Chronic toxicity tests would be especially helpful, because regulatory limits on chemicals are based mostly on chronic toxicity data in several jurisdictions (e.g., Canada, Europe) and are a more direct way to establish chronic exposure limits in other jurisdictions that otherwise tend to rely more on acute toxicity data (e.g., the United States). In addition, retesting of the taxa most sensitive to nano‐Ni could help to decrease uncertainties around the current toxicity estimates for freshwater and saltwater organisms.

Another way to help decrease uncertainty about effects of nano‐Ni in aquatic systems would be to conduct mesocosm or field studies. Although field studies usually are time and resource intensive and relatively expensive, mesocosm studies can be conducted under controlled conditions in relatively short time periods and can be relatively inexpensive, while providing useful information about population‐ and community‐level responses to chemical exposures (e.g., stream microcosms and mesocosms; Clements 2004; Clements et al. 2013). However, standard methods have not yet been established for mesocosm studies, and some types of mesocosms have low replicability (Giesy and Allred 1985).

#### Uncertainties in nano‐Ni exposure during toxicity tests.

Current uncertainty about the effects of nano‐Ni is increased by a general lack of measurements of particulate nano‐Ni and dissolved Ni concentrations during aquatic nano‐Ni toxicity tests. Reliance on nominal concentrations of nano‐Ni particles added to exposure waters is equally as tenuous as reliance on nominal concentrations of dissolved Ni in tests of the toxicity of Ni salts, because 1) NPs can be incorrectly dosed into the exposure water (via measurement error or accidental losses of particles), 2) NPs can settle out of the water column and accumulate at the bottom of an exposure chamber, and 3) NPs can partially dissolve in the exposure water. The first source of error could result in underestimation or overestimation of nano‐Ni toxicity. The second source of error could result in underestimation of nano‐Ni toxicity to water column organisms (which would experience toxic effects in a lower concentration of nano‐Ni than the nominal concentration indicates), but it could result in overestimation of nano‐Ni toxicity to bottom‐dwellers (which would experience toxic effects in a higher concentration of nano‐Ni than the nominal concentration indicates). Thus, in the same exposure chamber, bioavailability of nano‐Ni could differ among species and even among life stages of a given species because of a heterogeneous spatial distribution of the NPs (Schmidt et al. 2019). Addressing this second source of error would require concurrent measurements of 1) nano‐Ni and dissolved Ni concentrations in the water column and at the bottom of the exposure chamber, and 2) the exposure‐water chemistry during nano‐Ni toxicity tests. The third source of error could result in apparent toxicity of nano‐Ni particles actually being caused by dissolved Ni, which would still be an effect of the addition of nano‐Ni particles to the exposure water but otherwise would be difficult to predict without knowledge of the kinetics of dissolution of that physical–chemical form of nano‐Ni at that temperature in that type of water. In addition, other metals that might be impurities associated with the synthesis of NPs should be analyzed (Holden et al. 2016).

Because measurement of NP concentrations is challenging and not routine, nominal added concentrations of nano‐Ni have usually been reported. Sometimes the total Ni concentration is measured in exposure chambers as a surrogate for the nano‐Ni concentration, but that measurement does not reveal the concentrations of suspended and sedimented nano‐Ni particles or the concentration of dissolved Ni that released from the nano‐Ni particles. The concentrations of suspended nano‐Ni particles and dissolved Ni are of primary consideration for toxicity to water column organisms (e.g., swimming larvae and adults of fish, zooplankton, and insects; and suspended algae), whereas the concentrations of sedimented nano‐Ni particles at the bottom of the exposure chamber and the dissolved Ni in that microenvironment are of primary concern to bottom dwellers (e.g., benthic fish embryos and invertebrates). Some researchers measure the concentration of Ni that dissolves from the nano‐Ni particles, which sometimes has equaled 10 to 28% of the added nano‐Ni (Table 7). Those measurements are often taken in the absence of test organisms but would be more informative if taken when the organisms are (or have been) in the exposure water partway through or at the end of the toxicity test (or at the end of an exposure‐water renewal period). We strongly encourage measurement of NP concentrations in exposure chambers containing test organisms, because accurate characterization of exposure concentrations is crucial to understanding and explaining toxicity. Griffitt et al. (2008) provided an excellent example of a major difference in the amount of dissolution of nano‐Ni^0^ with and without *D. pulex* in the exposure water.

Most technical guidance documents for conducting toxicity tests with pelagic organisms that have been established by the Organisation for Economic Co‐operation and Development (OECD) set a ±20% limit for change in the nominal or initial measured concentration of the tested chemical during a test (Petersen et al. 2015). If that limit is exceeded, the test might need to be repeated unless “diligent efforts were made to attempt to maintain consistent exposure, the exposure was quantified on the basis of measured values, and measurements were made frequently during a test or medium renewal period” (Petersen et al. 2015: page 9538). However, “… for many [manufactured NPs], maintaining water column concentrations within ±20% of the initial concentration during ecotoxicity assays with or without medium renewal and without the use of dispersants or solvents is likely to be difficult if not logistically infeasible …” (Petersen et al. 2015: page 9538). Thus, practical guidance is needed on this aspect of conducting toxicity tests with NPs. Petersen et al. (2015) suggested ways to calculate toxicity endpoints in such challenging situations.

Murphy and Buriak (2015) and MacCuspie (2018) recommended best practices for the reporting of inorganic NPs, and Card and Magnuson (2010) proposed a “nanomaterial score” to complement ratings of toxicity test methods (see *Quality screening*). To those recommendations we add the use of several relatively recent analytical methods for quantifying concentrations of NPs that were reviewed by MacCuspie (2018) and Picó and Andreu (2019) including: liquid chromatography techniques; inductively coupled–plasma mass spectrometry (ICP–MS) coupled with various separation and selective‐extraction techniques, such as asymmetrical‐flow field‐flow fractionation (e.g., Mitrano et al. 2012); single‐particle ICP–MS (e.g., Montaño et al. 2016); and inductively coupled plasma–time‐of‐flight mass spectrometry (ICP–TOF–MS; e.g., Praetorius et al. 2017). Some of these relatively new techniques can be used to determine the number and mass concentrations and/or the size distributions of suspended and sedimented nano‐Ni particles in exposure waters.

Finally, it is also important to adequately characterize the exposure‐water chemistry in nano‐Ni toxicity tests, because dissolved Ni released from nano‐Ni can sometimes reach or exceed toxic concentrations in some water chemistries (Geitner et al. 2020; and see *Source of nano‐Ni toxicity: Nano‐Ni particles or dissolved Ni?*). However, a full set of water chemistry parameters that is useful for determining bioavailability of dissolved metals is seldom reported in NP toxicity studies (Handy et al. 2008b). At a minimum, temperature, pH, hardness, and DOC should be reported for freshwater exposures to allow for bioavailability normalization using approaches like the biotic ligand model (e.g., Nys et al. 2016). For saltwater exposures, temperature, pH, and salinity should be reported. Because DOC can be a toxicity‐modifying factor for marine exposures (Blewett et al. 2016), it should also be reported in saltwater toxicity tests. In addition, it would be helpful to report concentrations of major inorganic ions (Ca^2+^, Mg^2+^, Na^+^, K^+^, Cl^–^, SO_4_^2–^; Meyer et al. 2007) that could be used in bioavailability‐based models to predict the toxicity of the dissolved Ni (e.g., Nys et al. 2016). Uncertainty in predicting the toxicity of nano‐Ni will probably remain relatively high until bioavailability of the co‐occurring dissolved Ni can be accounted for. Geitner et al. (2020) recommended standard exposure media that could be used to harmonize toxicity test conditions, which would help to decrease uncertainty in among‐study comparisons.

## RECOMMENDATIONS

To help advance the ability to more appropriately compare nano‐Ni toxicity among various taxa and among various physical and chemical forms of nano‐Ni and dissolved Ni, and to ultimately improve prediction of the toxicity of nano‐Ni to aquatic organisms, we recommend the following actions related to toxicity test methods and the reporting of testing conditions and toxicity results.

The most pressing need related to the effects of nano‐Ni to aquatic organisms is conducting more, especially long‐term (chronic) toxicity tests with under‐represented freshwater and saltwater taxa (e.g., algae, filter‐feeding invertebrates, and fish other than *D. rerio*) at sensitive life stages. In addition, reporting of physical–chemical conditions of the toxicity‐testing systems needs to improve. First, most researchers do not adequately report enough characteristics of the nano‐Ni particles used in their toxicity tests, either in the dry form of the particles before they are added to the exposure water (e.g., particle size, extent of aggregation) or in the wet form of the particles in the actual exposure water (e.g., hydrodynamic size of the particles and surface charge; preferably with the test organisms also present). Second, almost no researchers report important physical conditions (e.g., type, duration, and intensity of lighting) and chemical conditions (e.g., pH, alkalinity, hardness, and concentrations of major inorganic ions and DOC) in their test systems. Third, almost no researchers monitor the concentrations of nano‐Ni particles and dissolved Ni in the water column and at the bottom of exposure chambers, especially in the vicinity of their test organisms.

Two methods to distinguish between dissolved Ni and nano‐Ni concentrations have been reported thus far in the literature, but are only applicable to laboratory exposure waters containing only nano‐Ni and dissolved Ni: 1) using filtration followed by high‐speed centrifugation to isolate the operationally defined dissolved Ni in the supernatant, with the operationally defined nano‐Ni in the pellet (Zhou et al. 2016); and 2) using ultrafiltration to isolate the operationally defined dissolved Ni in the filtrate, with the operationally defined nano‐Ni equaling the difference between the total Ni in the original sample and the dissolved Ni in the ultrafiltrate (Boran and Şaffak 2018). This micro‐environment monitoring is especially challenging; but without it, exposure concentrations that the organisms actually experience are highly uncertain. Attention to detail in these areas would help to advance development of reliable mechanistic models to predict nano‐Ni toxicity, and thus would contribute to more scientifically defensible regulation of nano‐Ni and incorporation into risk assessments.

Regarding conduct and overall design of toxicity tests, we recommend that researchers use standardized test conditions when testing the toxicity of nanomaterials, as also recommended by Zhou et al. (2016; e.g., US Environmental Protection Agency 2002a, 2002b; and OECD guidelines for the testing of chemicals, in the 200 series addressing effects on biotic systems). However, modified exposure methods will need to be developed to accommodate the presence of NPs (e.g., stirring or aeration to suspend the NPs during exposures of pelagic organisms; or careful deposition of NPs at the bottom of the chamber for exposures of benthic organisms, so as to not resuspend the NPs into the overlying water). Handy et al. (2012a, b), Kühnel and Nickel (2014), Holden et al. (2016), and Zhou et al. (2016) recommended modifications to standardized testing protocols, to accommodate special considerations needed when testing nanomaterials; and a standardized testing procedure for *Artemia* species (brine shrimp) exposed to nanomaterials in salt water was recently published (Johari et al. 2019).

We also recommend testing with previously untested chemical forms of nano‐Ni, and additional testing with the seldom tested forms, to decrease uncertainties around the general toxicity of nano‐Ni (see *Uncertainties*). Retesting of the taxa most sensitive to nano‐Ni could help to decrease uncertainties. We also recommend that mesocosm or field studies of nano‐Ni ecotoxicity be conducted, taking into account similar exposure concerns as apply to laboratory tests.

We recommend more studies testing the effects of toxicity‐modifying factors. Although the effects of dissolved constituents in the exposure water on the toxicity of dissolved Ni are relatively well known, the mechanisms and effects of the same factors on the toxicity of nano‐Ni particles are unknown (e.g., DOC, which might sorb onto and at least partially coat NPs). In addition, particle‐related factors such as dry particle size, dissolution, surface charge, agglomeration, and hydrodynamic size might affect the toxicity of nano‐Ni. However, some of the particle‐related factors (e.g., agglomeration and surface charge, which are somewhat related to each other) might be difficult to vary without also concurrently varying the exposure‐water chemistry (e.g., pH, DOC, ionic strength).

Regarding testing conditions and toxicity results, we recommend reporting concentrations of nanoparticulate and dissolved Ni measured in exposure waters during nano‐Ni tests, with the test organisms having been in the water to be analyzed (i.e., not just in a separate “analytical” chamber into which organisms were not added). We also recommend reporting the set of physical–chemical parameters in the exposure water that currently are required inputs into bioavailability‐based models of the toxicity of cationic metals (i.e., temperature, pH, alkalinity, hardness, and concentrations of DOC, Ca, Mg, Na, K, SO_4_, and Cl). As a minimum, survival, growth, and/or reproduction in each treatment should be reported, along with the water chemistry in each treatment if exposure conditions varied among the treatments. This should be done in addition to reporting summary statistics (e.g., acute L[E]C50s), and the results, including results in the controls and each treatment, could be included in a supporting information file if too large for the main text of a publication.

We also recommend development of standard protocols for NP characterization and reporting. Purity of the NPs, whether they were commercially supplied or synthesized by the researchers, is especially important to analyze. Researchers should not rely only on the manufacturer's product information when using commercially available NPs. This is especially important if concentrations are to be converted to mg Ni/L from known concentrations of mg NPs/L (see *Processing of toxicity data*), which we recommend so comparisons of toxicity that are more valid can be made among different chemical forms of particulate Ni and between nano‐Ni and dissolved Ni.

Regarding analysis of toxicity data, we recommend that researchers normalize their toxicity results based on bioavailability of the measured dissolved Ni concentrations in the exposure‐water chemistry (e.g., using a biotic ligand model; e.g., Nys et al. 2016) in addition to reporting the concentrations of dissolved Ni. To the extent possible, exposure concentrations should bracket 50% effects in acute tests and 10 to 20% effects in chronic tests, to provide definitive estimates of acute L(E)C50s and chronic L(E)C10s or L(E)C20s (instead of indefinite less‐than or greater‐than values).

Finally, we recommend development of quantitative models to predict the toxicity of nano‐Ni to aquatic organisms. As an example of simple predictive models, Kaweeteerawat et al. (2015: page 1105) demonstrated that “the probability of a [metal‐oxide NP] being toxic [to *E. coli*] increases as the hydration enthalpy becomes less negative and as the conduction band energy approaches those of biological molecules,” and Li et al. (2012) demonstrated an inverse relationship between population growth of *E. coli* and ROS generation by 6 metal‐oxide NPs. Kovalishyn et al. (2018) correlated toxicity endpoints (e.g., L[E]C50s) to a large number of physical, chemical, and biological input data in quantitative structure–property relationships using the Online Chemical Modeling Environment.

## CONCLUSIONS

The quality of the nano‐Ni toxicity database currently does not equal the quality of the dissolved‐Ni toxicity database, thus making it difficult to draw strong conclusions about the toxicity of nano‐Ni to aquatic organisms. Although many studies have demonstrated that nano‐Ni^0^, nano‐NiO, nano‐Ni oxides/hydroxides, and a variety of Ni‐containing CENs can cause lethal and/or sublethal effects, the lack of or incomplete reporting of crucial physical, chemical, and biological information would limit the use of much of the current nano‐Ni toxicity database for derivation of regulatory criteria/guidelines/environmental quality standards. Until the quality of the aquatic toxicity database for nano‐Ni is improved, it will be difficult to determine appropriate regulatory recommendations for nano‐Ni in surface waters.

Based on the available information, some common findings about NP toxicity are not supported for nano‐Ni. In our meta‐analysis, nano‐Ni^0^ and nano‐NiO generally did not differ in their toxicity (Table 3 and Figure 2), whereas too little systematic information exists to compare the toxicity of nano‐Ni^0^ and nano‐NiO with other chemical forms of nano‐Ni (e.g., other nano‐Ni oxides/hydroxides and individual Ni‐containing CENs). Currently, there is no evidence that the toxicity of nano‐Ni to aquatic organisms increases as the size of the NPs decreases (perhaps the opposite is more correct; Table 4). In addition, for the majority of organisms tested, nano‐Ni was not more toxic on a mass‐concentration basis (i.e., in concentration units of mg Ni/L) than dissolved Ni is in tests with Ni salts (Table 5). However, nano‐Ni particles sometimes appear to contribute more to observed toxicity than is contributed by the dissolved Ni released from the NPs in nano‐Ni exposure waters, in part because a large percentage of the total mass of Ni in the exposure water usually is in the NPs (i.e., in some exposure waters, insufficient Ni releases from the nano‐Ni particles to contribute much toxicity). However, the dissolved Ni released from nano‐Ni appears to be the dominant toxicant in some moderately low (e.g., a few percentage points) and in all of the relatively high percentages (e.g., 10–26%) of nano‐Ni dissolution (Table 7).

The most sensitive taxa tested were similar before and after applying the quality screening (Supplemental Data, Tables S5 and S6), thus providing evidence that the screening method did not skew the overall results. Supporting that conclusion are the facts that 1) the range of geometric mean acute L(E)C50s for nano‐Ni^0^ and nano‐NiO lies within the range of acute L(E)C50s for dissolved Ni, and 2) the range of geometric mean chronic TECs for nano‐Ni^0^ and nano‐NiO lies almost fully within the range of chronic NOECs or L(E)C10s for dissolved Ni, regardless of whether the studies passed or did not pass the quality screening.

No evidence suggests that any of the molecular, physiological, and structural mechanisms of nano‐Ni toxicity differ from the general pattern for many metal‐based nanomaterials (e.g., nano‐Cu, nano‐Zn), wherein the production of ROS and subsequent oxidative stress underlies the observed effects. However, the toxicity of nano‐Ni varies greatly among taxa, and the toxicity and mechanisms of toxicity can vary among life stages.

Physical factors in the design of toxicity tests can be important. For example, based on the few studies available, UV irradiation can increase dissolution of nano‐Ni particles and thus increase the toxicity of the nano‐Ni exposure. However, based on the only available study of its effectiveness, sonication of nano‐Ni particles before preparation of exposure waters did not alter the toxicity.

Overall, the most pressing immediate actions to address regulatory and risk assessment needs related to the effects of nano‐Ni to aquatic organisms are 1) conducting more, especially long‐term (chronic) toxicity tests with under‐represented freshwater and saltwater taxa at sensitive life stages, and 2) improving the reporting of physical–chemical conditions of the toxicity testing systems. First, most researchers do not adequately report enough characteristics of the nano‐Ni particles used in their toxicity tests, either in the dry form of the particles before they are added to the exposure water (e.g., size of the particles, and extent of aggregation) or in the wet form of the particles in the actual exposure water (e.g., hydrodynamic size and surface charge, preferably with the test organisms also present). Second, almost no researchers report important physical conditions (e.g., type, duration, and intensity of lighting) and chemical conditions (pH, alkalinity, hardness, and concentrations of major inorganic ions and DOC) in their test systems. Third, almost no researchers monitor the concentrations of nano‐Ni particles and dissolved Ni in the water column and at the bottom of exposure chambers, especially in the vicinity of their test organisms.

Although we have focused on nano‐Ni toxicity studies, most of the uncertainties we have identified are common across NP studies in general. Thus, our recommendations are also relevant in general to a wide variety of other compositions and forms of nanomaterials.

## Supplemental Data

The Supplemental Data are available on the Wiley Online Library at https://doi.org/10.1002/etc.4812.

## Author Contributions

T. Lyons‐Darden, E.R. Garman, C.E. Schlekat, and J.S. Meyer conceived and designed the present study. J.S. Meyer conducted the literature search and data analysis. J.S. Meyer, T. Lyons‐Darden, E.T. Middleton, E.R. Garman, and C.E. Schlekat contributed to the writing. E.T. Middleton and J.S. Meyer created the graphics.

## Supporting information

This article includes online‐only Supplemental Data.

Supporting information.

Supporting information.

## Data Availability

Data, associated metadata, and calculation tools are available from the corresponding author (jsmeyer@ALPsColorado.com).
