# Supplementary material for: Toxicity of Nanoparticulate Nickel to Aquatic Organisms: Review and Recommendations for Improvement of Toxicity Tests
Source: Environ Toxicol Chem. 2020 Aug 25;39(10):1861–83. doi: 10.1002/etc.4812 (PMC7590136; doi:10.1002/etc.4812)
Supplement: Supplementary file 1 — Supporting information. [file ETC-39-1861-s001.docx]

**Supporting Information #1**

**Toxicity of Nanoparticulate Nickel to Aquatic Organisms:
Review and Recommendations for Improvement of Toxicity Tests**

**LIST OF ABBREVIATIONS**

AF4-ICP-MS asymmetrical flow field flow fractionation inductively-coupled plasma mass spectrometry

CEN complex engineered nanomaterial (sensu Mahoney et al. 2016)

DOC dissolved organic carbon

DOM dissolved organic matter

EC50 median effect concentration; the concentration of a chemical that causes 50% impairment of a biological function or process (e.g., usually a decrease of survival, growth, or reproduction)

ICP-TOFMS inductively-coupled plasma time-of-flight mass spectrometry

LC50 median lethal concentration; the concentration of a chemical that causes 50% mortality (i.e., it results in 50% survival)

L(E)C50 median lethal (or effect) concentration; the concentration of a chemical that causes 50% impairment of a biological function or process (e.g., usually a decrease of survival, growth, or reproduction)

LOEC lowest observed effect concentration; the lowest tested concentration of a chemical in a toxicity test that caused a statistically significant difference in response from the control organisms

MBC minimum bactericidal concentration

MFC minimum fungicidal concentration

MIC minimum inhibitory concentration

nano-Ni nano-nickel (a collective term for all nickel-containing nanomaterials)

nano-NiO nanoparticles comprised predominantly of nickel oxide

nano-Ni^0^ nanoparticles comprised predominantly of elemental nickel

Ni nickel

NiO nickel oxide

Ni^0^ elemental nickel

NOEC no observed effect concentration; the highest tested concentration of a chemical in a toxicity test that did not cause a statistically significant difference in response from the control organisms

OS oxidative stress

ROS reactive oxygen species

SP-ICP-MS single-particle inductively-coupled plasma mass spectrometry

UV ultraviolet

**NANOMATERIAL DEFINITIONS**

The European Commission’s definitions of nanomaterial, particle, agglomerate, and aggregate are adopted for this risk analysis (EC 2011: page L 275/40):

“[A nanomaterial is a] natural, incidental or manufactured material containing particles, in an unbound state or as an aggregate or as an agglomerate and where, for 50% or more of the particles in the number size distribution, one or more external dimensions is in the size range 1 nm - 100 nm. In specific cases and where warranted by concerns for the environment, health, safety or competitiveness the number size distribution threshold of 50% may be replaced by a threshold between 1 and 50%.

… ‘particle’ means a minute piece of matter with defined physical boundaries;

… ‘agglomerate’ means a collection of weakly bound particles or aggregates where the resulting external surface area is similar to the sum of the surface areas of the individual components;

… ‘aggregate’ means a particle comprising of strongly bound or fused particles.”

Some other regulatory jurisdictions have slightly different definitions of nanomaterials (e.g., see review in EuMBC 2017), but a common theme among most of the definitions is that nanomaterials have at least one size dimension in the range of 1 to 100 nm (Sukhanova et al. 2018). Supplementing that definition, Shaw and Handy (2011: page 1084) suggested “…it may be prudent to also consider aggregates of [nanoparticles] that can be a few hundred nanometers wide.” In contrast, Auffan et al^.^ (2009: page 1) proposed that “evidence for novel size-dependent properties alone, rather than particle size, should be the primary criterion in any definition of nanoparticles when making decisions about their regulation for environmental, health and safety reasons” and concluded that “particles larger than about 30 nm do not in general show properties that would require regulatory scrutiny beyond that required for their bulk counterparts.” However, the more traditional size range of 1 to 100 nm is retained herein as the definition of nanomaterials.

**SUPPORTING INFORMATION METHODS**

**Quality screening of literature**

The compiled publications on the toxicity of nano-Ni to aquatic organisms were sorted into two groups: those that included primary reports of toxicity tests and physiological experiments, and those that did not (i.e., secondary reports such as review papers). Then we screened the primary publications for relevance (i.e., appropriate test organisms, exposure pathways, and toxicity endpoints for use in regulatory applications) and reliability (i.e., data quality).

Relevant major taxonomic groups of test organisms for this meta-analysis were defined as fish, amphibians, aquatic invertebrates, aquatic plants, aquatic macroalgae and microalgae, yeasts, fungi, bacteria, and viruses that might occur in aquatic systems. However, some of the bacterial and fungal taxa were only tested with the bacteria, fungi, and/or nano-Ni particles embedded in or spread on top of solidified agar, thus making it challenging to translate those results into meaningful aqueous exposure concentrations (i.e., we could not validly conclude what aqueous concentration of nano-Ni the organisms had experienced). For completeness, publications that reported results for bacteria and fungi tested on/in agar were included in the data-quality screening and in Tables S4 and S8; however, we excluded from the present analysis of the aquatic toxicity of nano-Ni, results in which the exposure to nano-Ni occurred in or on solidified agar.

The only exposure pathway included in the present meta-analysis is waterborne exposure to nano-Ni. Although exposure to nano-Ni in sediments is an important topic, few sediment-pathway studies were located, and the concentration units in those studies (mg/kg sediment) could not be directly compared to concentrations in the waterborne-exposure studies (e.g., mg/L). Thus, toxicity tests with organisms exposed to nano-Ni in sediments were not included in this meta-analysis. For a similar reason, dietborne-pathway studies were excluded. Additionally, toxicity tests conducted with exclusively terrestrial plants (e.g., wheat, radish, tomato; even in hydroponic exposure) and terrestrial invertebrates (e.g., ticks) exposed to nano-Ni were excluded from the meta-analysis.

Relevant toxicity endpoints included measures of survival, somatic growth, reproduction, and population growth (e.g., increased optical density in exposure water or growth medium containing bacteria, fungi, yeasts, or algae). Physiology and pathology endpoints that were not directly related to survival, somatic growth, reproduction, or population growth [e.g., production of ROS, hemoglobin, and serotonin; protein synthesis; activity of antioxidative enzymes; tissue lesions; cell proliferation] were considered only as biomarkers that might be used in an adverse outcome pathway. Therefore, metrics related to those physiology and pathology endpoints were not included in our tabulations of acute and chronic toxicity. Behavior (e.g., avoidance of a chemical, or impaired avoidance of a predator) is considered an important toxicity endpoint in some regulatory jurisdictions; however, behavioral response was quantified as a toxicity endpoint in only 7 of the 100 studies included in the present meta-analysis.

The 3 relevance criteria (test organism, exposure pathway, and toxicity endpoint) were incorporated into an evaluation of the study design for each toxicity test, in addition to consideration of whether a series of nanoparticle concentrations and a negative control were used. That study-design evaluation was the first of 10 categories in the quality-rating system we used to evaluate each toxicity test (Table S2). The other 9 categories in the quality-rating scheme were: source and purity of nanoparticles; toxicity test methods; chemistry of exposure water/medium; measured exposure concentrations of nanoparticles; measured exposure concentrations of dissolved metals; toxicity results; dry size of nanoparticles; physical-chemical characteristics of dry nanoparticles; and physical-chemical characteristics of nanoparticles in exposure water/medium.

Physical, chemical, and biological factors that affect the toxicity of dissolved and nanoparticulate Ni to aquatic organisms are important to report (MacCuspie 2018) and constituted major criteria in the rating categories used in the present meta-analysis. For example, impurities can bias toxicity results (source and purity of nanoparticles category). Toxicity can vary by age, life stage, and/or size (toxicity test methods category). Exposure-water chemistry [e.g., pH, alkalinity, hardness, and concentration of dissolved organic carbon (DOC)] can modify toxicity of dissolved Ni and possibly nano-Ni (chemistry of exposure water/medium category). Nominal (i.e., intended, but not measured) concentrations of metals do not necessarily equal the actual exposure concentrations (measured exposure concentrations of nanoparticles category, and measured exposure concentrations of dissolved metals category). And characteristics of the dry nanoparticles (e.g., size, morphology/shape, surface chemistry, crystallinity) and of the wet nanoparticles in the exposure water/medium (e.g., zeta potential, hydrodynamic size, agglomeration state, solubility in exposure water/medium) probably modify nanoparticle toxicity (dry size of nanoparticles category, physical-chemical characteristics of dry nanoparticles category, and physical-chemical characteristics of nanoparticles in exposure water/medium category).

Several publications have recommended ways to report physical-chemical characteristics of nanoparticles. For example, Murphy and Buriak (2015) and MacCuspie (2018) recommended best practices for the reporting of inorganic nanoparticles, including suggested analytical techniques. Card and Magnuson (2010) proposed a “nanomaterial score” to complement ratings of toxicity test methods, which evaluate the reporting of (1) agglomeration and/or aggregation, (2) chemical composition, (3) crystal structure/crystallinity, (4) particle size/size distribution, (5) purity, (6) shape, (7) surface area, (8) surface charge, (9) surface chemistry (including composition and reactivity), and (10) whether any characterization was conducted in the relevant experimental media. We incorporated those recommendations into the following quality-rating scheme for nano-Ni toxicity tests.

For each toxicity test, each of the 10 categories listed in Table S2 was assigned a rating of low (“L”), medium (“M”), or high (“H”), according to the criteria listed in that table. Then the ratings of all 10 categories were averaged to produce an overall rating of L, M, or H for each toxicity test. However, because of their special importance for the quality and reporting of study design, test methods, and nanoparticle characterization, we also designated 5 of those 10 categories as prime categories: study design; toxicity test methods; chemistry of exposure water/medium; toxicity results; and dry size of nanoparticles. If the lowest individual rating among these 5 prime rating categories was lower than the averaged overall rating, the final overall rating was decreased to that lowest prime-category rating. For example, an L rating for the toxicity test methods category would lower the overall rating to an L even if the averaged rating of all 10 categories for a given toxicity test was an M. Additionally, the toxicity results category received an automatic L rating if the authors did not confirm that the controls passed appropriate acceptability criteria (or if results presented for the controls did not demonstrate acceptable performance), unless a standardized protocol that includes specific control-performance requirements was followed for the toxicity test (e.g., Kovrižnych et al. 2013). Appropriate controls are important to avoid artifacts and misinterpretations of the effects of nanomaterials (Petersen et al. 2014; Petersen 2015).

Toxicity tests that received an overall rating of H or M were used in our meta-analysis; tests that were rated as L were excluded. This does not necessarily mean the scientific merit of an L-rated study was low. Instead, the L rating simply indicates that the toxicity test might receive low priority for inclusion in the derivation of a water quality criterion, water quality guideline, or environmental quality standard, depending on the regulatory jurisdiction under consideration. In the *Results and Discussion* in the main text and the *Recommendations* below, we suggest ways to improve aquatic toxicity studies with nanoparticles that would help to increase their quality ratings.

Even some of the toxicity tests that we rated as M might not be used by some regulatory jurisdictions, because we used relatively liberal criteria for some of the quality-rating categories. For example, an M rating for the chemistry of exposure water/medium category only required reporting of temperature, pH, and water hardness (i.e., DOC, which is an important determinant of metal toxicity, was not required for an M rating; as was alkalinity, another potentially important toxicity-modifying factor for some metals). And more important, an M or H rating for the study design category only required reporting of the nominal nano-Ni concentrations, even though actual exposure concentrations of nanoparticles can differ considerably from nominal concentrations because of particle settling, dosing errors, etc. (see *Uncertainties in nano-Ni exposure during toxicity tests* in the main text). However, without these relatively liberal rating criteria, few studies would have survived the quality screening (see *Results and Discussion* in the main text).

Several taxonomic assumptions had to be made. Although Pang et al. (2009) reported tests conducted with “*S. faecalis*”, we assumed they tested *Enterococcus faecalis* because the bacteria formerly called *Streptococcus faecalis* has been taxonomically reassigned to the genus *Enterococcus*. Likewise, although Kganyago et al. (2018) reported tests conducted with “*Enterobacter faecalis*”, we assumed they tested *Enterococcus faecalis* because the genus *Enterobacter* does not contain a species name of *faecalis*. Baek and An (2011) reported results for “*Streptococcus aureus*”, but we assumed they tested *Staphylococcus aureus* because the genus *Streptococcus* does not contain a species name of *aureus*. Ezhilarasi et al. (2016) reported a result for “*Staphylococcus pneumoniae*” in their Figure 8, but we assumed they tested *Streptococcus pneumoniae* (as they indicated in the text of that article), because the genus *Staphylococcus* does not contain a species name of *pneumoniae*. Although Khalil et al. (2018) reported results for “*Staphylococcus epidermis*”, we assumed the species name was “*epidermidis*” because the genus *Staphylococcus* does not contain a species name of *epidermis*.

For one study (Kheiri Hafshejani et al. 2018), we assumed the concentration units were incorrectly reported as mg/ml instead of as μg/ml (or equivalently as mg/L). This decision was based (1) on the unrealistically high exposure concentrations those authors reported (up to 13 mg nanoparticles/ml, which is equivalent to 1.3% weight/volume nanoparticles in the exposure waters) and (2) on the authors’ comparisons to toxic concentrations reported in other publications [which were mistakenly reported as mg/ml by Kheiri Hafshejani et al. (2018) instead of as the μg/ml units reported in the original publications]. In a similar instance of apparently incorrect concentration units (Kganyago et al. 2018), the corresponding author confirmed that the published units of mg/ml (i.e., equivalent to g/L) were intended to be μg/ml (i.e., equivalent to mg/L) [personal communication, Dr. Nomso Hintsho-Mbita, University of Limpopo, South Africa, 26 June 2019].

**Processing of toxicity data**

Consistent with the approach used in most regulatory and risk assessment settings, we divided the toxicity tests into acute and chronic exposure durations. Because chronic-toxicity data are favored in many regulatory jurisdictions [e.g., Canada (CCME 2007), Europe (ECHA 2008)], there is a general preference for those studies; however, we have also included acute-toxicity data because of their predominant use in some other jurisdictions (e.g., within the USA). Test-duration cutoffs that we used to differentiate between acute and chronic exposures for the major taxonomic groups that are included in this meta-analysis were: amphibians and fish – 4 d; invertebrates – 2 d; plants – 7 d; macroalgae – 2 d. In this analysis, acute-toxicity results were only represented by median lethal (or effect) concentrations [L(E)C50s], while chronic toxicity results were represented by no observed effect concentrations (NOECs), lowest observed effect concentrations (LOECs), and 10 percent lethal (or effect) concentrations [L(E)C10s].

When they were reported, L(E)C50s, L(E)C10s, NOECs, and LOECs were used as reported by a study’s authors. For bacteria and fungi, some authors also reported minimum inhibitory concentrations (MICs; analogous to LOECs) and minimum bactericidal (or fungicidal) concentrations (MBCs or MFCs). Various authors defined MIC and MBC somewhat differently. For example, Kheiri Hafshejani et al. (2018: page 22) defined MIC as the lowest concentration of nanoparticles that “lead to growth inhibition of bacteria” and MBC as the lowest concentration that “resulted in more than 99.9% reduction of the inoculums”; whereas Paul and Neogi (2019: page 3) defined MIC as “the least concentration of nanoparticle that inhibits any visible growth of bacteria after 24 h of incubation” and MBC as “the least concentration of nanoparticle that inhibits any visible growth of bacteria after 48-96 h of incubation.” Some other authors reported MICs and/or MBCs but did not provide their definitions. Thus, we accepted authors’ MICs and MBCs as reported, because insufficient information was provided to adjust the MICs and MBCs to any given set of definitions.

We selected the L(E)C10 endpoint as a conservative regression-based estimate of the chronic threshold effect concentration (TEC), although a 20% or 25% effect concentration is sometimes used as a less conservative estimate of a TEC. In some cases, we could additionally [or in lieu of an L(E)C10] estimate the TEC as the geometric mean of the NOEC and LOEC when dealing with chronic toxicity data, or analogously as the geometric mean of the MIC and the next-lower exposure concentration tested in some bacterial and fungal toxicity tests. The geometric mean of the NOEC and LOEC is sometimes referred to as a chronic value (ChV) or the maximum allowable toxicant concentration (MATC). If a TEC could be estimated by more than one of those three methods in any given toxicity test or experiment, the lowest of those values was adopted as the TEC for that test or experiment.

If the L(E)C10s and L(E)C50s were not specifically reported in a publication but sufficient concentration-response data were provided in the text, a table, or a figure, the L(E)Cx values were calculated using the log-probit regression option (two-parameter Gaussian tolerance distribution with logarithmic transformation of the exposure concentrations) in the United States Environmental Protection Agency’s Toxicity Relationship Analysis Program (TRAP), Version 1.30a (https://archive.epa.gov/med/med_archive_03/web/html/trap.html). If TRAP could not converge on a regression solution but the responses in two non-control exposure concentrations bracketed 50% impairment, the L(E)C50 was estimated by log-linear interpolation [i.e., a linear relationship was assumed for response versus log_10_(concentration) in that region]. Similarly, if TRAP could not converge on a solution, the L(E)C10 was estimated by log-linear interpolation between the two non-control exposures that bracketed 10% impairment, if available. But if the response in the lowest non-control concentration exceeded 50% (or 10%) impairment, the L(E)C50 [or the L(E)C10] was recorded as less than the lowest non-control concentration; and if the response in the highest non-control concentration was less than 50% (or 10%) impairment, the L(E)C50 [or the L(E)C10] was recorded as greater than the highest non-control concentration.

If the NOEC and/or LOEC were not specifically reported in a publication but statistical significance levels between exposure concentrations and the controls were indicated in the text, a table, or a figure, the NOEC and/or LOEC were inferred from the indicated non-significant and significant exposure concentrations. Additionally, if an MIC, MBC, or MFC was not stated but the magnitudes of survival or effect responses in the exposure concentrations were reported in the text, a table, a figure, the MIC, MBC, or MFC sometimes could be estimated as less than or greater than one of the exposure concentrations.

If more than one nano-Ni^0^ and/or nano-NiO L(E)C50 that passed the quality screening was available for a given taxon, the geometric mean of all the available L(E)C50s that passed the quality screening for that taxon was calculated. Similarly, if more than one nano-Ni^0^ and/or nano-NiO TEC that passed the quality screening was available for a given taxon in separate tests/experiments within a study or in separate studies, the geometric mean of all the available TECs that passed the quality screening for that taxon was calculated. If an L(E)C50 or TEC was an indefinite less-than or greater-than value, the geometric mean for that taxon was calculated as if that value was an exact number; however, the resulting geometric mean is presented in tables and figures as a less-than or a greater-than value. If the compilation of L(E)C50 or TEC values that passed the quality screening for a given taxon contained at least one less-than value and at least one greater-than value, the resulting geometric mean L(E)C50 or TEC for that taxon is presented as an approximate value with a tilde (~) preceding the number.

Per ECHA (2008) guidance, the L(E)C50 for algal population-growth inhibition is considered a short-term toxicity concentration, but the L(E)C10 in the same test can be considered a long-term toxicity concentration. We adopted this same approach for toxicity tests that were conducted with yeasts, fungi, bacteria, and viruses.

Although most authors reported exposure concentrations as mg of nanomaterial/L (or sometimes as the equivalent units of μg of nanomaterial/ml), we converted all concentrations of Ni-containing nanomaterials (and bulk particles) to units of mg Ni/L. For example, as a default, we multiplied mg NiO/L by 0.7878 to convert to mg Ni/L, unless the authors reported a measured Ni percentage in the nanomaterial [e.g., Paul and Neogi (2019) reported their nano-NiO contained 81% Ni, and Wang et al. (2010) reported their nano-NiO contained 93.12% Ni]; and we assumed nano-Ni^0^ particles contained 100% Ni. If an analyzed composition of a CEN was not reported, its L(E)C50 and TEC are reported as less than the reported values that were based on mass of the entire nanomaterial. The conversion of concentration units to a common basis of mg Ni/L allows a more direct comparison of Ni content among different chemical forms of nano-Ni (e.g., between nano-Ni^0^ and nano-NiO) and between nano-Ni and bulk Ni-containing particles or dissolved Ni salts, thus allowing more appropriate toxicity comparisons.

**SUPPORTING INFORMATION RESULTS**

**Composition of database – Additional information**

Chemical forms of nano-Ni and the taxa that have been used in nano-Ni toxicity tests are listed in Tables S3 and S4. Quantitative results [L(E)C50s, TECs, MICs] of toxicity tests that were conducted in an aqueous-based medium are listed in Tables S5 (nano-Ni^0^), S6 (nano-NiO), and S7 (Ni-containing CENs), where the final overall rating of each toxicity test (L, M, or H) is also indicated. Results of toxicity tests with nano-Ni^0^ and nano-NiO that survived the quality screening (i.e., with a final overall rating of M or H) are summarized by test organism in Tables 1 and 2, respectively, in the main text. Studies not included in this meta-analysis because they received a final overall rating of L are listed in Table S8, along with the reason(s) for that rating. Details of the scores assigned in each of the individual rating categories for each study are contained in the Supporting Information Excel file.

One hundred primary-research publications reported a total of 307 tests that resulted in toxicity and/or physiology information for fish, amphibians, aquatic invertebrates, aquatic plants, macroalgae, microalgae, yeasts, fungi, bacteria, and a virus exposed to a total of 38 chemical forms of nano-Ni [nano-Ni^0^, nano-NiO, nano-Ni_2_O_3_, nano-Ni(OH)_2_, and 34 CENs with other metals or organic-carbon-containing chemicals; Tables S3 and S4]. Seventy-three freshwater and saltwater taxa were tested [24 bacteria (plus 4 microbial assemblages), 16 invertebrates, 11 fungi, 8 fish, 2 microalgae, 2 plants, 2 protozoa, 1 amphibian, 1 macroalga, 1 yeast, and 1 virus].

A majority of the toxicity studies were conducted with organisms exposed to either nano-Ni^0^ or nano-NiO. Of the 304 combinations of nano-Ni form x biological species x publication listed in Table S4, 24.3% used nano-Ni^0^ and 34.5% used nano-NiO; the remainder used Ni-containing CENs (37.5%), other nano-Ni-oxides/hydroxides (1.3%), or nano-Ni in mixtures with other nanomaterials (2.3%).

However, only 31 publications survived the quality screening, narrowing the number of taxa included in the meta-analysis to 22 (8 invertebrates, 6 bacteria, 3 fish, 2 microalgae, 1 plant, 1 amphibian, and 1 yeast). The main deficiencies that eliminated studies included conducting exposures to nano-Ni in or on solidified agar, not adequately describing the toxicity test method or not referring to a standardized test method, not adequately reporting exposure-water chemistry, and not reporting control acceptability. A surprisingly large 13.5% of the reported toxicity tests in 9% of the publications had unspecified test durations (41 of the tests, in 9 of the publications). Only 53 of the combinations of nano-Ni form x biological species x publication survived the quality screening [32.1% used nano-Ni^0^ and 37.7% used nano-NiO; the remainder used Ni-containing CENs (28.3%) or other nano-Ni-oxides/hydroxides (1.9%)].

A total of 53 publications reported acute L(E)C50s and/or chronic L(E)C10s, NOECs, LOECs, and/or MICs for aquatic organisms exposed to nano-Ni^0^ or nano-NiO in aqueous media, or they contained concentration-response data from which we calculated acute L(E)C50s or at least one of the chronic TECs. Of the 53 publications, 46 resulted in acute L(E)C50s for 39 aquatic taxa: 2 freshwater fish, 12 invertebrates (7 freshwater, 5 saltwater), 1 freshwater plant, 2 freshwater microalgae, 1 saltwater macroalga, 1 yeast, 5 fungi, 13 bacteria, 1 microbial assemblage, and 1 virus (Table S5). Thirty-four of the 53 publications resulted in chronic L(E)C10s, NOECs, LOECs, and/or MICs for 32 aquatic taxa: 4 freshwater fish, 2 invertebrates (1 freshwater, 1 saltwater), 1 freshwater plant, 2 freshwater microalgae, 1 yeast, 5 fungi, 15 bacteria, 1 microbial assemblage, and 1 virus (Table S6). However, only 25 of those 53 publications survived the quality screening, narrowing the number of publications that contained acceptable acute L(E)C50s to 24 [17 taxa: 1 freshwater fish, 8 invertebrates (3 freshwater, 5 saltwater), 2 freshwater microalgae, 1 yeast, 5 bacteria] and the number that contained acceptable chronic L(E)C10s, NOECs, LOECs, and/or MICs to 16 [12 taxa: 1 freshwater fish, 2 invertebrates (1 freshwater, 1 saltwater), 1 freshwater plant, 2 freshwater microalgae, 1 yeast, 5 bacteria].

Thirty-one publications reported results for aquatic organisms exposed to Ni-containing CENs; and 21 of those 31 resulted in acute L(E)C50s or chronic L(E)C10s or MICs in aqueous media (Table S7). The tested taxa included 3 freshwater fish, 1 freshwater amphibian, 4 freshwater invertebrates, 1 freshwater microalga, 2 fungi, 13 bacteria, 3 microbial assemblages, and 1 virus. However, only 8 of those 31 publications survived the quality screening, narrowing the number of publications that resulted in acute L(E)C50s to 8 (9 taxa: 3 freshwater fish, 1 freshwater amphibian, 1 freshwater invertebrate, 4 bacteria) and the number of publications that resulted in chronic L(E)C10s or MICs in aqueous media to 5 (5 taxa: 1 freshwater invertebrate, 4 bacteria).

Ten taxa (1 freshwater fish, 1 freshwater invertebrate, 2 freshwater microalgae, 6 bacteria) had results allowing comparison of the toxicity of Ni^0^ and NiO using the same test methods in the same water chemistry within the same study. However, only 5 of those 10 taxa had results that survived the quality screening (1 freshwater fish, 1 freshwater invertebrate, 1 freshwater microalga, 2 bacteria; Table 3 in the main text).

For six taxa [1 freshwater fish, 3 invertebrates (1 freshwater, 2 saltwater), 1 freshwater plant, 1 freshwater microalga], toxicity results could be compared among at least two sizes of nano-Ni using the same test methods in the same water chemistry within the same study, and all of those studies survived the quality screening (Table 4 in the main text).

One taxon (*Lemna gibba*, a freshwater plant; Oukarroum et al. 2015) had data for comparison of the toxicity of nano-NiO to the toxicity of bulk NiO particles, but that study did not survive the quality screening.

Nine taxa [1 freshwater fish, 5 invertebrates (2 freshwater, 3 saltwater), 1 freshwater alga, 1 yeast, 1 bacteria] had data for comparison of the toxicity of nano-Ni to the toxicity of a dissolved Ni salt using the same test methods in the same water chemistry within the same study. However, only 8 of those 9 taxa had results that survived the quality screening [1 freshwater fish, 5 invertebrates (2 freshwater, 3 saltwater), 1 freshwater alga, 1 yeast; Table 5 in the main text].

**Study quality – Additional information**

Most nano-Ni toxicity studies reviewed for this meta-analysis had several deficiencies in the design of the study and/or reporting of results. The most important are the following.

Of the 304 combinations of nano-Ni form x biological species x publication listed in Table S4, 122 were tested only in or on solidified agar (thus making it challenging to translate those results into meaningful aqueous exposure concentrations). Those results are not included in the summary results in Tables 1 and 2 in the main text and in the individual-test results in Tables S5 and S6. Overall, 11 of the 28 bacterial taxa and microbial assemblages (*Bacillus cereus*, *Enterobacter aerogenes*, *Enterobacter* sp., *Escherichia hermannii*, *Klebsiella* sp., *Lactobacillus* sp., *Pneumonia typhus*, *Proteus vulgaris*, *Salmonella typhimurium*, *Streptococcus pneumoniae*, *Vibrio cholerae*) and 6 of the 11 fungal taxa (*Alternaria solani*, *Aspergillus flavus*, *Fusarium oxysporum*, *Fusarium solani*, *Mucor racemosus*, *Rhizoctonia solani*) were tested only in or on solidified agar and thus are not included in Tables 1 and 2 in the main text and in Tables S5 and S6. Another 37 of the 304 combinations were tested in aqueous-based exposure medium and also in or on solidified agar within the same study. The results of those agar-based toxicity tests are not included in Tables 1 and 2 in the main text and in Tables S5 and S6, but the results of the tests conducted in aqueous-based exposure medium are included. In 2 of the combinations of nano-Ni form x biological species x publication, the authors did not identify the type of exposure medium; thus, those studies were also screened-out.

The total number of toxicity tests (307) exceeds the number of combinations of nano-Ni form x biological species x publication (304) listed in Table S4 because more than one toxicity test was conducted with some combinations of nano-Ni form x biological species. In the total of 307 separate toxicity tests compiled for the present analysis (listed in Supporting Information Excel file), almost all of the reported nano-Ni concentrations were nominal (i.e., they were based on the amount of nano-Ni intended to be added to the exposure waters and were not measured in the exposure waters). In only 2 studies, the authors attempted to quantify the actual nano-Ni concentrations in the exposure waters, either by filtration followed by high-speed centrifugation (Zhou et al. 2016) or by difference between total Ni and ultra-filtered Ni concentrations (Boran and Șaffak 2018).

In other toxicity test-related areas, the authors of 88 of the 307 toxicity tests did not state the reference for a standardized toxicity test method or did not report details of a test procedure that was consistent with a standardized test method (e.g., was not comparable to a population-growth test for microalgae) or appropriate for the taxon being tested (e.g., was not a population-growth test for bacteria). Additionally, authors for 186 of the toxicity tests did not report minimal physical-chemical details of the exposure water/medium or cite a recipe if a reconstituted exposure water/medium was used; and at least minimal quantitative toxicity results or summary metrics (e.g., LCx values) were not reported for 143 of the tests.

Regarding nanoparticle characterization, dry size of the nano-Ni particles (average size, range of sizes, and/or a size distribution) was not measured by the authors or reported from the commercial supplier in 33 of the 307 toxicity tests. No other physical-chemical characteristic of the dry nanoparticles were reported for 55 of the tests, and no other physical-chemical characteristic of the nanoparticles after their addition into the exposure water/medium (e.g., hydrodynamic size or zeta potential, both of which are relatively easy to measure) was reported for 196 of the tests.

Because the presence of nanoparticles in the exposure water in toxicity tests introduces a new physical form of toxicant, it is especially important to characterize the particles well. Some researchers reported a relatively thorough set of physical-chemical characteristics of the nano-Ni particles in dry and wet forms in their toxicity tests. Reported dry-particle characteristics included source of the particles, chemical composition, purity, average size and/or the size distribution, specific surface area, and density, usually relying on the manufacturer’s product information when purchased (although a manufacturer’s product information, including chemical composition, is not always accurate). Reported wet-particle characteristics included hydrodynamic diameter, zeta potential (a measure of average surface charge on the nanoparticles), and polydispersity index (a measure of the uniformity of size of the wet particles in the exposure water). However, only some (or none) of these wet-particle characteristics were routinely measured, in part because special instruments are needed. Even when reported, the wet-particle characteristics often were measured only at the start of a test and sometimes not in the actual exposure water.

**Toxicity of Ni-containing CENs – Additional information**

A relatively definitive toxicity comparison appears to be possible between a Ni^0^-Cu^0^ CEN (Argueta-Figueroa et al. 2014) and a NiO-CuO CEN (Paul and Neogi 2019; Table S7). The Ni^0^-Cu^0^ CEN was considerably less toxic than the NiO-CuO CEN [e.g., 1-h L(E)C50s for population growth of *Escherichia coli* (bacteria) were 7.03 and <0.309 mg Ni/L for Ni^0^-Cu^0^ and NiO-CuO, respectively; and 1-h L(E)C50s for population growth of *Staphylococcus aureus* (bacteria) were 12.2 and <0.309 mg Ni/L, respectively]. An obvious possible explanation is that the combination of the oxides (NiO and CuO) was more toxic than the combination of the elemental forms of Ni and Cu. However, the atomic Cu:Ni ratios also differed between the two CENs (1.05:1 for the Ni^0^-Cu^0^ combination versus 1.65:1 for the NiO-CuO combination), and acute L(E)C50s for dissolved Cu usually are lower than for dissolved Ni (e.g., compare ranges of L(E)C50s for Cu and Ni in Appendix A in Meyer et al. 2007). Additionally, different exposure media (Luria Bertani broth for NiO-CuO, but an unspecified broth for Ni^0^-Cu^0^) also might have contributed to the difference in toxicity, especially because Cu bioavailability can be strongly modified by complexation with organic ligands (e.g., Meyer et al. 2007).

**Nano-Ni^0^ versus nano-NiO toxicity – Additional information**

Based on the available data presented in the main text, nano-Ni^0^ and nano-NiO generally did not differ in their toxicity to aquatic organisms. This conclusion might be explained from a chemical-fate perspective, because the surface of a dry nano-Ni^0^ particle might be expected to oxidize when it is exposed to air and/or after it enters oxic water, and thus might be expected to form at least a surface layer of NiO on the particle (Lambers et al. 1996; Zhou et al. 2016). Alternatively (or in addition), a layer of Ni(OH)_2_ or other Ni-oxide might form on nano-Ni^0^ and nano-NiO surfaces, especially at circumneutral and higher pH values [i.e., in water having a pH greater than or equal to approximately 7, because the concentration of hydroxyl ions (OH-) increases as pH increases]. Either way, both types of nano-Ni particle might be expected to have similar surface chemistry and presumably similar biological reactivity to aquatic organisms in identical water chemistry. However, nano-Ni^0^ and nano-NiO may differ in human-health effects in non-aquatic exposure pathways (Buxton et al. 2019).

**Molecular, physiological, and structural responses – Additional information**

Nanoparticles can be internalized by endocytosis (envelopment of particles by an external membrane, followed by release of the resulting endosome into the cell’s cytoplasm; Shaw and Handy 2011; Jimeno-Romero et al. 2019). It is also possible that small nanoparticles having a hydrophobic coating (e.g., coated with phospholipids) or having no net charge might be internalized by diffusing through a cell membrane (Shaw and Handy 2011; Jimeno-Romero et al. 2019). For example, these conditions might be met by nanoparticles whose surfaces are coated with mucus secreted by an organism to which the nanoparticle is adsorbed, or by nanoparticles whose surfaces are at the isoelectric point.

An important consideration related to internalization is that nanoparticles can be coated with a “corona”, which is a layer of dissolved and/or particulate organic matter sorbed to the particle surface (Jimeno-Romero et al. 2019). When the corona develops while the particle is in the exposure water, it is referred to as an “eco-corona” comprised of natural organic matter, polysaccharides, colloids, and/or exopolymeric substances; but when the corona develops inside an organism or transforms after the nanoparticle is internalized, it is referred to as a “bio-corona” comprised of internally-secreted proteins, polysaccharides, and cellular/body fluids (Canesi and Corsi 2016). Eco-coronas can disguise a metal-containing nanoparticle and alters its surface charge and/or extent of aggregation, thus altering its interaction with the surface of an organism and its potential internalization. In protein-containing culture media used in laboratory experiments, this potential bias (called the “protein corona paradigm”; Katsumiti and Cajaraville 2019) should be considered in study design and interpretation of results. Bio-coronas can alter the toxicity of internalized nanoparticles by, for example, increasing their sorption to surfaces of organelles (possibly increasing toxicity) and/or by decreasing the rate of their dissolution inside cells (possibly decreasing toxicity). Thus, intrinsic properties of Ni-containing nanoparticles alone might not be sufficient to accurately predict the details of their toxicity to aquatic organisms (Corsi and Grassi 2019); however, little research is available to illuminate the role(s) played by coronas in nano-Ni toxicity.

**Source of nano-Ni toxicity: Nano-Ni particles or dissolved Ni? – Additional information**

Most of the authors’ valid conclusions in Table 7 in the main text about the extent of the contribution of dissolved Ni to observed toxicity were based on dissolved Ni concentrations measured in the exposure waters in nano-Ni toxicity tests when compared to concentrations of dissolved Ni needed to cause the same effect (e.g., mortality) in parallel Ni salt-only toxicity tests. However, that line of evidence does not preclude toxicity caused by dissolved Ni released from nano-Ni particles in close proximity to or contacting organisms, where the dissolved Ni concentrations might be considerably higher than in the exposure water away from the microenvironment surrounding the organism.

In a clever departure for determining the contribution of dissolved Ni to nano-Ni toxicity, Lin et al. (2011, 2013) eliminated embryo hatching impairment in *Danio rerio* (a freshwater fish) by adding DTPA (a metal chelator) to exposure water that contained nano-NiO. They concluded that this demonstrated a major contribution of dissolved Ni to the observed toxicity. That is, they concluded the DTPA complexed Ni^2+^ ions and thus decreased the bioavailability of the dissolved Ni considerably, thereby decreasing the hatching impairment caused by the nano-NiO. However, that presumed toxicity-modification mechanism does not preclude possible modification of the surface of the nano-NiO particles by DTPA, thus also decreasing another possible contribution of the particles to the observed toxicity. Additionally, complexation of Ni^2+^ with DTPA does not distinguish between toxicity caused by dissolved Ni in the exposure water and by possibly higher dissolved Ni concentrations in the microenvironment surrounding the organism.

Water chemistry appears to at least partially control the dissolution of nano-Ni particles, thus at least partially controlling the contribution of dissolved Ni to nano-Ni toxicity. Based on the available information in Table 7 in the main text, nano-Ni particles are generally less soluble in sea water than in fresh water. Although the ranges of the percent dissolution of nano-Ni particles overlap considerably between saltwater (~0.02-8%) and freshwater (~0.05-26%) exposures, 2 of the 3 saltwater entries had <2% dissolution while only 3 of the 11 freshwater entries had <2% dissolution. The chemical form of the nano-Ni did not appear to affect dissolution, because results for nano-Ni^0^ and nano-NiO were distributed throughout the ranges of dissolution percentages for freshwater and saltwater exposures. Additionally, percent dissolution of nano-Ni did not appear to be related to the dry or hydrodynamic (wet) size of the particles (Table 7 in the main text). However, common to all exposure waters, the hydrodynamic size of the particles usually greatly exceeded the dry size (Table 7 in the main text), meaning agglomeration and the accompanying increased sedimentation of the particles (Zhou et al. 2016) affected the quantity and quality of exposure of the organisms to nano-Ni.

A major factor affecting dissolution of nano-Ni particles might have been the presence of organic matter in the exposure water. Four of the 14 entries in Table 7 in the main text had >10% dissolution of nano-Ni particles (all in fresh water). Two of those 4 exposure waters contained added alginate (a polysaccharide of the C_6_H_7_O_6_^-^ ion), which contains a carboxylate moiety that might have complexed Ni^2+^ and thus increased the amount of Ni dissolved from the nano-Ni particles [both exposures were in fresh water with 100 mg alginate/L added (~0.6 mM; Lin et al. 2011, 2013)]. One of the other 2 waters contained OECD algal growth medium (Sousa et al. 2018b), which contains EDTA (a strong chelator of metals). In contrast, only 2 of the 11 exposure waters in which dissolution of nano-Ni was <10% contained dissolved organic matter that might have been able to complex Ni and thus increase the dissolved Ni concentration. Both of those exposures were in freshwater-based media: YEP (yeast extract-peptone-dextrose) broth and MES [2-(N-morpholino) ethane-sulfonic acid] buffer containing 20 g glucose/L (both in Sousa et al. 2018a). The last of the 4 entries in Table 7 in the main text that had >10% dissolution of nano-Ni used dechlorinated tap water in the toxicity test and did not report the DOC concentration (Griffitt et al. 2008).

Overall, nano-Ni and dissolved Ni are similar but importantly different in some of their physical and chemical interactions with organisms (Ispas et al. 2009; Shaw and Handy 2011). In their similarities, both cause OS and its associated molecular, physiological, and structural responses that can impair survival, growth, and/or reproduction of organisms. Figure 5 in Gomes et al. (2019) illustrates the commonalities and a few differences in adverse outcome pathways for an invertebrate exposed to nano-Ni or dissolved Ni.

The major toxicity differences between nano-Ni and dissolved Ni are related to their physical and chemical behaviors (Shaw and Handy 2011), including agglomeration and increased surface area:volume ratio (which might result in increased biological reactivity). Physically, nano-Ni particles are not thought to enter cells via uptake channels or paracellular diffusion and thus are mainly incorporated via endocytosis, in contrast to dissolved Ni that can pass through uptake channels. Additionally, nano-Ni particles can adsorb/adhere to surfaces of organisms, whereas dissolved Ni can only adsorb to organisms and cannot physically interfere with organismal functioning (e.g., dissolved Ni does not physically irritate gill and gut epithelia, damage skin, and impair movement of invertebrates, filtering of water, or transmission of light into photosynthetic cells like particles can). Chemically, the behavior and toxicity of dissolved Ni can be modeled as an equilibrium system (despite exhibiting many important biodynamic features; e.g., Luoma and Rainbow 2005), but dynamic processes dominate for nanoparticles (Shaw and Handy 2011). Examples of non-equilibrium, dynamic processes include attachment of particles to surfaces of organisms; endocytosis; production of ROS; and partial dissolution in the exposure water, at cell surfaces, or inside cells. These complexities present challenges for modeling the fate and effects of nano-Ni in aqueous systems.

**SUPPORTING INFORMATION REFERENCES**

Abdelwahab M, Salahuddin N, Gaber M, Mousa M. 2018. Poly(3-hydroxybutyrate)/polyethylene glycol-NiO nanocomposite for NOR delivery: Antibacterial activity and cytotoxic effect against cancer cell lines. *Int J Biol Macromol* 114:717-727.

Ananpattarachai J, Boonto Y, Kajitvichyanukul P. 2016. Visible light photocatalytic antibacterial activity of Ni-doped and N-doped Ti0_2_ on *Staphylococcus aureus* and *Escherichia coli* bacteria. *Environ Sci Pollut Res* 23:4111-4119.

Argueta-Figueroa L, Morales-Luckie RA, Scougall-Vilchis RJ, Olea-Mejía. 2014. Synthesis, characterization and antibacterial activity of copper, nickel and bimetallic Cu-Ni nanoparticles for potential use in dental materials. *Prog Nat Sci Mater Int* 24:321-328.

Ates M, Demir V, Arslan Z, Camas M, Celik F. 2016. Toxicity of engineered nickel oxide and cobalt oxide nanoparticles to *Artemia salina* in seawater. *Water Air Soil Pollut* 227:70.

Auffan M, Rose J, Bottero JY, Lowry GV, Jolivet J-P, Wiesner MR. 2009. Towards a definition of inorganic nanoparticles from an environmental, health and safety perspective. *Nat Nanotechnol* 4:634-641.

Baek Y-W, An Y-J. 2011. Microbial toxicity of metal oxide nanoparticles (CuO, NiO, ZnO, and Sb_2_O_3_) to *Escherichia coli*, *Bacillus subtilis*, and *Streptococcus aureus*. *Sci Total Environ* 409:1603-1608.

Bagirov VA, Sizova EA, Miroshnikova EP, Gavrish IA, Konovalov AV. 2019. Intestinal microbiocenosis disorders in *Danio rerio* (Hamilton, 1882) and inhibition of protective mechanisms under nickel-containing nanoparticle-induced effects. *Inland Water Biol* 12:115-123.

Bhushan M, Kumar Y, Periyasamy L, Viswanath AK. 2019. Fabrication and a detailed study of antibacterial properties of α-Fe_2_O_3_/NiO nanocomposites along with their structural, optical, thermal, magnetic and cytotoxic features. *Nanotechnology* 30:185101.

Blaise C, Gagné F, Férard JF, Eullaffroy P. 2008. Ecotoxicity of selected nano-materials to aquatic organisms. *Environ Toxicol* 23:591-598.

Bozich J, Hang M, Hamers R, Klaper R. 2017. Core chemistry influences the toxicity of multicomponent metal oxide nanomaterials, lithium nickel manganese cobalt oxide, and lithium cobalt oxide to *Daphnia magna*. *Environ Toxicol Chem* 36:2493-2502.

Buxton S, Garman E, Heim KE, Lyons-Darden T, Schlekat CE, Taylor MD, Oller AR. 2019. Concise review of nickel human health toxicology and ecotoxicology. *Inorganics* 7:89.

Canesi L, Corsi I. 2016. Effects of nanomaterials on marine invertebrates. *Sci Total Environ* 565:933-940.

Card JW, Magnuson BA. 2010. A method to assess the quality of studies that examine the toxicity of engineered nanomaterials. *Int J Toxicol* 29:402-410.

CCME (Canadian Council of Ministers of the Environment). 2007. A protocol for the derivation of water quality guidelines for the protection of aquatic life 2007. Canadian Council of Ministers of the Environment, Winnipeg, MB, Canada.

Chaudhary J, Tailor G, Yadav BL, Michael O. 2019. Synthesis and biological function of nickel and copper nanoparticles. *Heliyon* 5:e01878.

Chaudhary RG, Tanna JA, Gandhare NV, Rai AR, Juneja HD. 2015. Synthesis of nickel nanoparticles: Microscopic investigation, an efficient catalyst and effective antibacterial activity. *Adv Mater Lett* 6:990-998.

Chen JL, Steele TWJ, Stuckey DCC. 2018. The effect of Fe_2_NiO_4_ and Fe_4_NiO_4_Zn magnetic nanoparticles on anaerobic digestion activity. *Sci Total Environ* 642:276-284.

Cheng R, Kanga M, Zhuang S, Wang S, Zheng X, Pan X, Shi L, Wang J. 2019. Removal of bacteriophage f2 in water by Fe/Ni nanoparticles: Optimization of Fe/Ni ratio and influencing factors. *Sci Total Environ* 649:995-1003.

Corsi I, Grassi G. 2019. The role of ecotoxicology in the eco-design of nanomaterials for water remediation. In Blasco J, Corsi L, eds, *Ecotoxicology of Nanoparticles in Aquatic Systems*. CRC Press, Boca Raton, FL, USA, pp 219-229.

Din MI, Nabi AG, Rani A, Aihetasham A, Mukhtar M. 2018. Single step green synthesis of stable nickel and nickel oxide nanoparticles from *Calotropis gigantea*: Catalytic and antimicrobial potentials. *Environ Nanotechnol Monitor Manage* 9:29-36.

Dong H, Cheng Y, Lu Y, Hou K, Zhang L, Li L, Wang B, Wang Y, Ning Q, Zeng G. 2019. Comparison of toxicity of Fe/Ni and starch-stabilized Fe/Ni nanoparticles toward *Escherichia coli*. *Sep Purif Technol* 210:504-510.

EC (European Commission). 2011. Commission recommendation of 18 October 2011 on the definition of nanomaterial (2011/696/EU). *Offic J Eur Union*.

ECHA (European Chemicals Agency). 2008. Guidance on information requirements and chemical safety assessment. Chapter R.10: Characterization of dose [concentration]-response for environment. Accessed 2020 February 25. Available from: <https://echa.europa.eu/guidance-documents/guidance-on-information-requirements-and-chemical-safety-assessment>

EuMBC (European Masterbatchers and Compounders). 2017. Nanomaterial product registries. Position paper - 2 May 2017. Accessed 2020 February 25. Available from: <https://www.compounders.eu/documents>

Ezhilarasi AA, Vijaya JJ, Kaviyarasu K, Kennedy LJ, Ramalingam RJ, Al-Lohedan HA. 2018. Green synthesis of NiO nanoparticles using *Aegle marmelos* leaf extract for the evaluation of in-vitro cytotoxicity, antibacterial and photocatalytic properties. *J Photochem Photobiol B* 180:39-50.

Ezhilarasi AA, Vijaya JJ, Kaviyarasu K, Maaza M, Ayeshamariam A, Kennedy LJ. 2016. Green synthesis of NiO nanoparticles using *Moringa oleifera* extract and their biomedical applications: Cytotoxicity effect of nanoparticles against HT-29 cancer cells. *J Photochem Photobiol B* 164:352-360.

Gabrielson J, Kühn I, Colque-Navarro P, Hart M, Iversen A, McKenzie D, Möllby R. 2003. Microplate-based microbial assay for risk assessment and (eco)toxic fingerprinting of chemicals. *Anal Chim Acta* 485:121-130.

Gallo A, Boni R, Buttino I, Tosti E. 2016. Spermiotoxicity of nickel nanoparticles in the marine invertebrate *Ciona intestinalis* (Ascidians). *Nanotoxicology* 10:1096-1104.

Garza-Cervantes JA, Escárcega-González CE, Castro EDB, Mendiola-Garza G, Marichal-Cancino BA, López-Vázquez MA, Morones-Ramirez JR. 2019. Antimicrobial and antibiofilm activity of biopolymer-Ni, Zn nanoparticle biocomposites synthesized using *R. mucilaginosa* UANL-001L exopolysaccharide as a capping agent. *Int J Nanomedicine* 2019:2557-2571.

Golkhatmi FM, Bahramain B, Mamarabadi M. 2017. Application of surface modified nano ferrite nickel in catalytic reaction (epoxidation of alkenes) and investigation on its antibacterial and antifungal activities. *Mater Sci Eng C* 78:1-11.

Gomes SIL, Roca CP, Scott-Fordsmand JJ, Amorim MJB. 2019. High-throughput transcriptomics: Insights into the pathways involved in (nano) nickel toxicity in a key invertebrate test species. *Environ Pollut* 245:131-140.

Gong N, Shao K, Feng W, Lin Z, Liang C, Sun Y. 2011. Biotoxicity of nickel oxide nanoparticles and bio-remediation by microalgae *Chlorella vulgaris*. *Chemosphere* 83:510-516.

Gong N, Shao K, Li G, Sun Y. 2016. Acute and chronic toxicity of nickel oxide nanoparticles to *Daphnia magna*: The influence of algal enrichment. *NanoImpact* 3-4:104-109.

Griffitt RJ, Luo J, Gao J, Bonzongo J-C, Barber DS. 2008. Effects of particle composition and species on toxicity of metallic nanomaterials in aquatic organisms. *Environ Toxicol Chem* 27:1972-1978.

Gunsolus IL, Hang MN, Hudson-Smith NV, Buchman JT, Bennett JW, Conroy D, Mason SE, Hamers RJ, Haynes CL. 2017. Influence of nickel manganese cobalt oxide nanoparticle composition on toxicity toward *Shewanella oneidensis* MR-1: Redesigning for reduced biological impact. *Environ Sci Nano* 4:636-346.

Han Z, Zhangmeng, Lv C-X. 2012. Bioaccumulation and toxicity of NiO nanoparticles. *Adv Mat Res* 518-523:942-945.

Hang MN, Gunsolus IL, Wayland H, Melby ES, Mensch AC, Hurley KR, Pedersen JA, Haynes CL, Hamers RJ. 2016. Impact of nanoscale lithium nickel manganese cobalt oxide (NMC) on the bacterium *Shewanella oneidensis* MR‑1. *Chem Mater* 28:1092-1100.

Hang MN, Hudson-Smith NV, Clement PL, Zhang Y, Wang C, Haynes CL, Hamers RJ. 2018. Influence of nanoparticle morphology on ion release and biological impact of nickel manganese cobalt oxide (NMC) complex oxide nanomaterials. *ACS Appl Nano Mater* 1:1721-1730.

Hanna SK, Miler RJ, Zhou D. Keller AA, Lenilhan HS. 2013. Accumulation and toxicity of metal oxide nanoparticles in a soft-sediment estuarine amphipod. *Aquat Toxicol* 142-143:441-446.

He C-S, Ding R-R, Wang Y-R, Li Q, Wang Y-X, Mu Y. 2019a. Insights into short- and long-term effects of loading nickel nanoparticles on anaerobic digestion with flocculent sludge. *Environ Sci Nano* 6:2820-2831.

He C-S, Huang L, Ding R-R, Yang H-Y, Wang Y-X, Li J, Mu Y. 2019b. Progressive stress response of the anaerobic granular sludge to nickel nanoparticles: Experimental investigations and mathematic modelling. *Environ Sci Nano* 6:1536-1548.

Helen SM, Rani MHE. 2015. Characterization and antimicrobial study of nickel nanoparticles synthesized from *Dioscorea* (Elephant yam) by green route. *Int J Sci Res* 4:216-219.

Hoop M, Shen Y, Chen X-Z, Mushtaq F, Luliano LM, Sakar MS, Petruska A, Loessner MJ, Nelson BJ, Pané S. 2016. Magnetically driven silver-coated nanocoils for efﬁcient bacterial contact killing. *Adv Funct Mater* 26:1063-1069.

Horst AK. 2009. Antimicrobial effects of metal oxide nanoparticles. *2009 NNIN REU Res Accomplish*:12-13.

Hou J, Liu H, Wang L, Duan L, Li S, Wang X. 2018a. Molecular toxicity of metal oxide nanoparticles in *Danio rerio*. *Environ Sci Technol* 52:7996-8004.

Hou Y-X, Abdullah H, Kuo D-H, Leu S-J, Gultom NS, Su C-H. 2018b. A comparison study of SiO_2_/nano metal oxide composite sphere for antibacterial application. *Compos B* 133:166-176.

Hrenovic J, Milenkovic J, Daneu N, Kepcja RM, Rajic N. 2012. Antimicrobial activity of metal oxide nanoparticles supported onto natural clinoptilolite. *Chemosphere* 88:1103-1107.

Iqbal J, Abbasi BA, Mahmood T, Hameed S, Munir A, Kanwal S. 2019. Green synthesis and characterizations of nickel oxide nanoparticles using leaf extract of *Rhamnus virgata* and their potential biological applications. *Appl Organomet Chem* 33:e4950.

Ispas C, Andreescu D, Patel A, Goia DV, Andreescu S, Wallace KN. 2009. Toxicity and developmental defects of different sizes and shape nickel nanoparticles in zebrafish. *Environ Sci Technol* 43:6349-6356.

Jayaseelan C, Rahuman AA, Ramkumar R, Perumal P, Rajakumar G, Kirthi AV, Santhoshkumara T, Marimuthu S. 2014. Effect of sub-acute exposure to nickel nanoparticles on oxidative stress and histopathological changes in mozambique tilapia, *Oreochromis mossambicus*. *Ecotoxicol Environ Saf* 107:220-228.

Jeyaraj Pandian C, Palanivel R, Dhanasekaran S. 2016. Screening antimicrobial activity of nickel nanoparticles synthesized using *Ocimum sanctum* leaf extract. *J Nanopart* 2016:4694367.

Jimeno-Romero A, Kohl Y, Marigómez I, Soto M. 2019. Intracellular fate and toxic effects on cells of aquatic organisms as revealed by microscopy. In Blasco J, Corsi L, eds, *Ecotoxicology of Nanoparticles in Aquatic Systems*. CRC Press, Boca Raton, FL, USA, pp 169-188.

Jośko I, Oleszczuk P, Skwarek E. 2016. The bioavailability and toxicity of ZnO and Ni nanoparticles and their bulk counterparts in different sediments. *J Soils Sed* 16:1798-1808.

Kanold JM, Wang J, Brümmer F, Šiller L. 2016. Metallic nickel nanoparticles and their effect on the embryonic development of the sea urchin *Paracentrotus lividus*. *Environ Pollut* 212:224-229.

Kanwal Z, Raza MA, Manzoor F, Riaz S, Jabeen G, Fatima S, Naseem S. 2019. A comparative assessment of nanotoxicity induced by metal (silver, nickel) and metal oxide (cobalt chromium) nanoparticles in *Labeo rohita*. *Nanomaterials* 9:309.

Katsumiti A, Cajaraville MP. 2019. In vitro toxicity testing with bivalve mollusc and fish cells for the risk assessment of nanoparticles in the aquatic environment. In Blasco J, Corsi L, eds, *Ecotoxicology of Nanoparticles in Aquatic Systems*. CRC Press, Boca Raton, FL, USA, pp 62-98.

Kganyago P, Mahlaule-Glory LM, Mathipa MM, Ntsendwana B, Mketo N, Mbita Z, Hintsho-Mbita NC. 2018. Synthesis of NiO nanoparticles via a green route using *Monsonia burkeana*: The physical and biological properties. *J Photochem Photobiol B* 182:18-26.

Khalil AT, Ovais M, Ullah I, Ali M, Shinwari ZK, Hassan D, Maaza M. 2018. *Sagretia thea* (Osbeck.) modulated biosynthesis of NiO nanoparticles and their in vitro pharmacognostic, antioxidant, and cytotoxic potential. *Artif Cells Nanomed Biotechnol* 46:838-852.

Kheiri Hafshejani B, Mirhosseini M, Dashtestani F, Hakimian F, Haghirosadat BF. 2018. Antibacterial activity of nickel and nickel hydroxide nanoparticles against multidrug resistance *K. pneumonia* and *E. coli* isolated urinary tract. *Nanomed J* 5:19-26.

Ko K-S, Koh D-C, Kong IC. 2017. Evaluation of the effects of nanoparticle mixtures on *Brassica* seed germination and bacterial bioluminescence activity based on the theory of probability. *Nanomaterials* 7:344.

Ko K-S, Koh D-C, Kong IC. 2018. Toxicity evaluation of individual and mixtures of nanoparticles based on algal chlorophyll content and cell count. *Materials* 11:121.

Ko K-S, Kong IC. 2014. Toxic effects of nanoparticles on bioluminescence activity, seed germination, and gene mutation. *Appl Microbiol Biotechnol* 98:3295-3303.

Kovrižnych JA, Sotníková R, Zeljenková D, Rollerová E, Szabová E. 2014. Long-term (30 days) toxicity of NiO nanoparticles for adult zebrafish *Danio rerio*. *Interdiscip Toxicol* 7:23-26.

Kovrižnych JA, Sotníková R, Zeljenková D, Rollerová E, Szabová E, Wimmerová S. 2013. Acute toxicity of 31 different nanoparticles to zebrafish (*Danio rerio*) tested in adulthood and in early life stages – comparative study. *Interdiscip Toxicol* 6:67-73.

Kumar H, Rani R, Salar R. 2010. Reverse micellar synthesis, characterization and antibacterial study of nickel nanoparticles. In Mladenov V, Psarris K, Mastorakis N, Caballero A, Vachtsevanos G, eds, *Advances in Control, Chemical Engineering, Civil Engineering and Mechanical Engineering*. World Scientific and Engineering Academy and Society, Stevens Point, WI, USA, pp 88-94.

Lambers ES, Dykstal CN, Seo JM, Rowe JE, Holloway PH. 1996. Room-temerature oxidation of Ni(110) at low and atmospheric oxygen pressures. *Oxid Met* 45:301-321.

Lashkenari MS, Ghorbani M, Naghibi H, Khalaj P. 2019. Synthesis and characterization of polyrhodanine/nickel ferrite nanocomposite with an effective and broad spectrum antibacterial activity. *Polymer-Plastics Technol Mater* 58:1461-1470.

Le TT, Murugesan K, Kim E-J, Chang Y-S. 2014. Effects of inorganic nanoparticles on viability and catabolic activities of *Agrobacterium* sp. PH-08 during biodegradation of dibenzofuran. *Biodegradation* 25:655-668.

Li Y, Ran X, Zonglai L. 2017. Cytotoxicity of NiO nanoparticles and its conversion inside *Chlorella vulgaris*. *Chem Res Chin Univ* 33:107-111.

Lin S, Zhao Y, Ji Z, Ear J, Chang CH, Zhang H, Low-Kam C, Yamada K, Meng H, Wang X, Liu R, Pokhrel S, Mädler L, Damoiseaux R, Xia T, Godwin HA, Lin S, Nel AE. 2013. Zebrafish high-throughput screening to study the impact of dissolvable metal oxide nanoparticles on the hatching enzyme, ZHE_1_. *Small* 9:1776-1785.

Lin S, Zhao Y, Xia T, Meng H, Ji Z, Liu R, George S, Xiong S, Wang X, Zhang H, Pokhrel S, Mädler L, Damoiseaux R, Lin S, Nel AE. 2011. High content screening in zebrafish speeds up hazard ranking of transition metal oxide nanoparticles. *ACS Nano* 5:7284-7295.

Luoma SN, Rainbow PS. 2005. Why is metal bioaccumulation so variable? Biodynamics as a unifying concept. *Environ Sci Technol* 39:1921-1931.

MacCuspie RI. 2018. Characterization of nanomaterials for nanoEHS studies. In Hull MS, Bowman DM, eds, *Nanotechnology Environmental Health and Safety: Risks, Regulation, and Management*. Elsevier, Amsterdam, The Netherlands, pp 59-82.

Mahoney S, Najera M, Bai Q, Burton EA, Veser G. 2016. The developmental toxicity of complex silica-embedded nickel nanoparticles is determined by their physicochemical properties. *PLoS One* 11:e0152010.

Meyer JS, Clearwater SJ, Doser TA, Rogaczewski MJ, Hansen JA. 2007. *Effects of Water Chemistry on the Bioavailability and Toxicity of Waterborne Cadmium, Copper, Nickel, Lead, and Zinc to Freshwater Organisms*. SETAC Press, Pensacola, FL, USA.

Mishra P, Thakur S, Mahapatra DM, Wahid ZA, Liu H, Singh L. 2018. Impacts of nano-metal oxides on hydrogen production in anaerobic digestion of palm oil mill effluent – A novel approach. *Int J Hydrogen Energy* 43:2666-2676.

Mohebbi M, Allafchian A, Kameli P, Jalali SA. 2019. Preparation and structural characterisation of magnetic NiFe_2_O_4_@ABS@Ag nanocompound with antibacterial property. *Micro Nano Lett* 14:445-449.

Morgaleva T, Morgalev Y, Gosteva I, Morgalev S. 2015. Research of nickel nanoparticles toxicity with use of aquatic organisms. *IOP Conf Ser Mater Sci Eng* 98:012012.

Morgaleva T, Morgalev Y, Gosteva I, Morgalev S, Nesterenya D. 2017. Embryotoxicity of poorly soluble nanoparticles at various stages of zebrafish development. *AIP Conf Proc* 1899:050004.

Murphy CJ, Buriak JM. 2015. Best practices for the reporting of colloidal inorganic nanomaterials. *Chem Mater* 27:4911-4913.

Naik MM, Naik HSB, Nagaraju G, Vinuth M, Vinu K, Rashmi SK. 2018. Effect of aluminium doping on structural, optical, photocatalytic and antibacterial activity on nickel ferrite nanoparticles by sol-gel auto-combustion method. *J Mater Sci Mater Electron* 29:20395-20414.

Nazdar N, Imani A, Noori F, Moghanlou KS. 2018. Effect of silymarin supplementation on nickel oxide nanoparticle toxicity to rainbow trout (*Oncorhynchus mykiss*) fingerlings: Pancreas tissue histopathology and alkaline protease activity. *Iran J Sci Technol Trans A Sci* 42:353-361.

Niemuth NJ, Curtis BJ, Hang MN, Gallagher MJ, Fairbrother DH, Hamers RJ, Klaper RD. 2019. Next-generation complex metal oxide nanomaterials negatively impact growth and development in the benthic invertebrate *Chironomus riparius* upon settling. *Environ Sci Technol* 53:3860-3870.

Nogueira V, Lopes I, Rocha- Santos TAP, Rasteiro MG, Abrantes N, Gonçalves F, Soares AMVM, Duarte AC, Pereira R. 2015. Assessing the ecotoxicity of metal nano-oxides with potential for wastewater treatment. *Environ Sci Pollut Res* 22:13212-13224.

Oleszczuk P, Jośko I, Skwarek E. 2015. Surfactants decrease the toxicity of ZnO, TiO_2_ and Ni nanoparticles to *Daphnia magna*. *Ecotoxicology* 24:1923-1932.

Oukarroum A, Barhoumi L, Samadani M, Dewez D. 2015. Toxic effects of nickel oxide bulk and nanoparticles on the aquatic plant *Lemna gibba* L. *Biomed Res Int* 2015:501326.

Oukarroum A, Zaidi W, Samadani M, Dewez D. 2017. Toxicity of nickel oxide nanoparticles on a freshwater green algal strain of *Chlorella vulgaris*. *Biomed Res Int* 2017:9528180.

Özel RE, Wallace KN, Andreescu S. 2014. Alterations of intestinal serotonin following nanoparticle exposure in embryonic zebrafish. *Environ Sci Nano* 1:27-36.

Pang H, Lu Q, Li Y, Gao F. 2009. Facile synthesis of nickel oxide nanotubes and their antibacterial, electrochemical and magnetic properties. *Chem Commun* 2009:7542-7544.

Paul D, Neogi S. 2019. Synthesis, characterization and a comparative antibacterial study of CuO, NiO and CuO-NiO mixed metal oxide. *Mater Res Express* 6:055004.

Peng B, Zhang X, Aarts DGAL, Dullens RPA. 2018a. Superparamagnetic nickel colloidal nanocrystal clusters with antibacterial activity and bacteria binding ability. *Nat Nanotechnol* 13:478-482.

Peng G, He Y, Zhao M, Yu T, Qin Y, Lin S. 2018b. Differential effects of metal oxide nanoparticles on zebrafish embryos and developing larvae. *Environ Sci Nano* 5:1200-1207.

Perachiselvi M, Bagavathy JJSMS, Feiona TA, Krishnaveni P, Laksmi EP, Swetha V, Leema MM, Britto SJ, Annadurai G. 2018. Fabrication of nickel oxide nanoparticles for antibacterial and photocatalytic activity. *Res J Life Sci Bioinform Pharmaceut Chem Sci* 4:749.

Petersen EJ. 2015. Control experiments to avoid artifacts and misinterpretations in nanoecotoxicology testing. NIST Special Publication 1200-11. National Institute of Standards and Technology, Gaithersburg, MD, USA.

Petersen EJ, Henry TB, Zhao J, MacCuspie RI, Kirschling TL, Dobrovolskaia MA, Hackley V, Xing B, White JC. 2014. Identification and avoidance of potential artifacts and misinterpretations in nanomaterial ecotoxicity measurements. *Environ Sci Technol* 48:4226-4246.

Pirsaheb M, Azadi NA, Miglietta ML, Sayadi MH, Blahova J, Fathi M, Mansouri B. 2019. Toxicological effects of transition metal-doped titanium dioxide nanoparticles on goldfish (*Carassius auratus*) and common carp (*Cyprinus carpio*). *Chemosphere* 215:904-915.

Poornavaishnavi C, Gowthami R, Srikanth K, Bramhachari PV, Venkatramaiah N. 2019. Nickel nanoparticles induces cytotoxicity, cell morphology and oxidative stress in bluegill sunfish (BF-2) cells. *Appl Surf Sci* 483:1174-1181.

Rajakumar G, Rahuman AA, Velayutham K, Ramyadevi J, Jeyasubramanian K, Marikani A, Elango G, Kamaraj C, Santhoshkumar T, Marimuthu S, Zahir AA, Bagavan A, Jayaseelan C, Kirthi AV, Iyappan M, Siva C. 2013. Novel and simple approach using synthesized nickel nanoparticles to control blood-sucking parasites. *Vet Parasitol* 191:332-339.

Rajan PI, Vijaya JJ, Jesudoss SK, Kaviyarasu K, Kennedy LJ, Jothiramalingam R, Al-Lohedan HA, Vaali-Mohammed M-A. 2017. Green-fuel-mediated synthesis of self-assembled NiO nano-sticks for dual applications—photocatalytic activity on Rose Bengal dye and antimicrobial action on bacterial strains. *Mater Res Express* 4:085030.

Rajivgandhi G, Maruthupandy M, Querob F, Li W-J. 2019. Graphene/nickel oxide nanocomposites against isolated ESBL producing bacteria and A549 cancer cells. *Mater Sci Eng* C 102:829-843.

Ramalingam R, Fazil MHUT, Verma NK, Arunachalam KD. 2019. Green synthesis, characterization and antibacterial evaluation of electrospun nickel oxide nanofibers. *Mater Lett* 256:126616.

Ray SK, Dhakal D, Sohng JK, Kim S-Y, Lee SW. 2018. Efficient inactivation of *Pseudomonas aeruginosa* by Cu/Co-α-NiMoO_4_ in visible light. *Chem Eng J* 347:366-378.

Sabouri Z, Akbari A, Hosseini HA, Hashemzadeh A, Darroudi M. 2019. Eco-friendly biosynthesis of nickel oxide nanoparticles mediated by okra plant extract and investigation of their photocatalytic, magnetic, cytotoxicity, and antibacterial properties. *J Clust Sci* 30:1425-1434.

Santhoshkumar A, Kavitha HP, Suresh R. 2016. Hydrothermal synthesis, characterization and antibacterial activity of NiO nanoparticles. *J Adv Chem Sci* 2:230-232.

Sedghi R, Shariati M, Zarehbin MR, Soorki AA. 2017. High-performance visible light-driven Ni-ZnO/rGO/nylon-6 & Ni-ZnO/rGO/nylon-6/Ag nanofiber webs for degrading dye pollutant and study their antibacterial properties. *J Alloys Compounds* 729:921-928.

Shaw BJ, Handy RD. 2011. Physiological effects of nanoparticles on fish: A comparison of nanometals versus metal ions. *Environ Int* 37:1083-1097.

Sousa CA, Soares HMVM, Soares EV. 2018a. Nickel oxide (NiO) nanoparticles disturb physiology and induce cell death in the yeast *Saccharomyces cerevisiae*. *Appl Microbiol Biotechnol* 102:2827-2838.

Sousa CA, Soares HMVM, Soares EV. 2018b. Toxic effects of nickel oxide (NiO) nanoparticles on the freshwater alga *Pseudokirchneriella subcapitata*. *Aquat Toxicol* 204:80-90.

Sousa CA, Soares HMVM, Soares EV. 2018c. Nickel oxide (NiO) nanoparticles induce loss of cell viability in yeast mediated by oxidative stress. *Chem Res Toxicol* 31:658-665.

Sousa CA, Soares HMVM, Soares EV. 2019. Nickel oxide nanoparticles trigger caspase- and mitochondria-dependent apoptosis in the yeast *Saccharomyces cerevisiae*. *Chem Res Toxicol* 32:245-254.

Sudhasree S, Shakila Banu A, Brindha P, Kurian GA. 2014. Synthesis of nickel nanoparticles by chemical and green route and their comparison in respect to biological effect and toxicity. *Toxicol Environ Chem* 96:743-754.

Sukhanova A, Bozrova S, Sokolov P, Berestovoy M, Karaulov A, Nabiev I. 2018. Dependence of nanoparticle toxicity on their physical and chemical properties. *Nanoscale Res Lett* 13:44.

Svartz G, Papa M, Gosatti M, Jordán M, Soldati A, Samter P, Guraya MM, Coll CP, Catán SP. 2017. Monitoring the ecotoxicity of γ-Al_2_O_3_ and Ni/γ-Al_2_O_3_ nanomaterials by means of a battery of bioassays. *Ecotoxicol Environ Saf* 144:200-207.

Svartz G, Sandoval MT, Gosatti M, Perez Catán S, Pérez Coll C. 2019. Lethality, neurotoxicity, morphological, histological and cellular alterations of Ni-Al nanoceramics on the embryo-larval development of *Rhinella arenarum*. *Environ Toxicol Pharmacol* 69:36-43.

Udhaya PA, Bessy TC, Meena M. 2019. Antibacterial activity of nickel and magnesium substituted ferrite nanoparticles synthesized via self-combustion method. *Mater Today Proc* 8:169-175.

Wang D, Lin Z, Wang T, Yao Z, Qin M, Zheng S, Lu W. 2016. Where does the toxicity of metal oxide nanoparticles come from: The nanoparticles, the ions, Or a combination of both? *J Hazard Mater* 308:328-334.

Wang Z, Lee Y-H, Wu B, Horst A, Kang Y, Tang YJ, Chen D-R. 2010. Anti-microbial activities of aerosolized transition metal oxide nanoparticles. *Chemosphere* 80:525-529.

Xu J-J, Cheng Y-F, Xu L-Z-J, Liu Y-Y, Zhu B-Q, Fan N-S, Huang B-C, Jin R-C. 2019. The revolution of performance, sludge characteristics and microbial community of anammox biogranules under long-termNiO NPs exposure. *Sci Total Environ* 649:440-447.

Zhang W, Li Y, Niu J, Chen Y. 2013. Photogeneration of reactive oxygen species on uncoated silver, gold, nickel, and silicon nanoparticles and their antibacterial eﬀects. *Langmuir* 29:4647-4651.

Zhou C, Carotenuto Y, Vitiello V, Wu C, Zhang J, Buttino I. 2018. De novo transcriptome assembly and differential gene expression analysis of the calanoid copepod *Acartia tonsa* exposed to nickel nanoparticles. *Chemosphere* 209:163-172.

Zhou C, Vitiello V, Casals E, Puntes VF, Iamunno F, Pellegrini D, Changwen W, Benvenuto G, Buttino I. 2016. Toxicity of nickel in the marine calanoid copepod *Acartia tonsa*: Nickel chloride versus nanoparticles. *Aquat Toxicol* 170:1-12.

Table S1. Journals searched for publications related to the ecotoxicity of nickel-containing nanomaterials.

_____________________________________________________________________________________________

ACS Nano

Advances in Natural Sciences: Nanoscience and Nanotechnology

All ACS journals

Ambio

Applied Microbiology and Biotechnology

Aquatic Toxicology

Archives of Environmental Contamination and Toxicology

Archives of Toxicology

Chemosphere

Ecotoxicology

Ecotoxicology and Environmental Safety

Environmental Chemistry Letters

Environmental Nanotechnology, Monitoring and Management

Environmental Pollution

Environmental Science: Nano

Environmental Science and Pollution Research

Environmental Toxicology

Environmental Toxicology and Chemistry

Eurasian Soil Science

IEE Transactions of NanoBioscience

Integrated Environmental Assessment and Management

International Nano Letters

Journal of Environmental Quality

Journal of Experimental Nanoscience

Journal of Hazardous Materials

Journal of Nano Research

Journal of Nanobiotechnology

Journal of Nanoparticle Research

Journal of Nanoscience and Nanotechnology

Journal of Nanoscience and Technology

Nano Biotechnology

Nano Impact

Nano Letters

Nano Research

Nano Today

Nano-Micro Letters

Nanomaterials

Nanoscale

Nanoscale Research Letters

Nanoscience and Technology

Nanotechnology, Science and Applications

Nanotoxicology

Open Nano

Plant Physiology and Biochemistry

PLoS ONE

Science of the Total Environment

Small

Water, Air, Soil Pollution

_____________________________________________________________________________________________

Table S2. Criteria used in quality ratings of nanoparticulate-nickel toxicity publications. L(E)Cx = concentration causing x% mortality or other adverse effect (e.g., immobilization, or decreased growth or reproduction); LOEC = lowest observed effect concentration; NOEC = no observed effect concentration.

|  |  | Criteria for quality rating |  |
| --- | --- | --- | --- |
| Category ^a^ | High | Medium | Low |
| **Quality of toxicity testing and reporting of results** | | | |
| Study design * | • Used a relevant test organism; and  • Used a waterborne pathway of exposure; and  • Used a relevant toxicity endpoint (i.e., survival, somatic growth, reproduction, population growth, or a direct correlate); and  • Used a series of nanoparticle concentrations and a negative control; and  • Reported measured nanoparticle concentrations. | • Used a relevant test organism; and  • Used a waterborne pathway of exposure; and  • Used a relevant toxicity endpoint (i.e., survival, somatic growth, reproduction, population growth, or a direct correlate); and  • Used only one nanoparticle concentration and a negative control; and  • Reported nominal or measured nanoparticle concentration. | • Did not use a relevant test organism; or  • Did not use a waterborne pathway of exposure (e.g., dietborne pathway, or a sprayed aerosol); or the exposure pathway was not apparent; or  • Did not use a relevant toxicity endpoint (e.g., a physiological endpoint not well-correlated with survival, somatic growth, reproduction, or population growth); or  • Did not use a negative control; or  • Did not report concentrations tested. |
| Source and purity of nanoparticles | • Reported the commercial source of the nanoparticles, or reported (or provided a reference to) the procedure used to synthesize the nanoparticles in the authors’ laboratory; and  • Reported a chemical analysis of the elemental composition/purity of the nanoparticles performed by the authors. | • Reported that the nanoparticles were synthesized in the authors’ laboratory, but did not provide sufficient details of (or a reference to) the procedure; or  • Reported a qualitative analysis of the elemental composition/purity of the nanoparticles; or  • Reported the purity of the nanoparticles, as stated by the commercial supplier. | • Did not report the source of the nanoparticles; or  • Did not report the purity of the nanoparticles. |
| Toxicity-test methods * | • Exposed the organisms to the nanoparticles in water or an aqueous-based medium (e.g., not solidified agar); and  • Stated the reference for a standardized test method (e.g., ASTM, OECD, USEPA); and  • Stated the age, lifestage, and/or size of the organisms; and  • If relevant, provided details on dispersion (or sonication) or other forms of manipulation of the nanoparticles before exposure of organisms; and  • Allowed equilibration of nanoparticles in the exposure waters/media (to allow time for aggregation and settling), and reported the amount of equilibration time. | • Exposed the organisms to the nanoparticles in water or an aqueous-based medium (e.g., not solidified agar); and  • Reported details of a test procedure that was consistent with a standardized test method or was appropriate for the taxon being tested; and  • Stated the age, lifestage, and/or size of the organisms; and  • If relevant, provided details on dispersion (or sonication) or other forms of manipulation of the nanoparticles before exposure of organisms; and  • Clearly reported that the nanoparticles were not equilibrated in the exposure waters/media. | • Did not expose the organisms to the nanoparticles in water or an aqueous-based medium (e.g., in solidified agar); or  • Did not report sufficient details of the test procedure, or the procedure was not consistent with a standardized test method or was not appropriate for the taxon being tested; or  • Did not state the age, lifestage, or size of the organisms; or  • Reported insufficient or no details about the preparation of the stock solution of nanoparticles or the exposure waters/media; or  • Reported insufficient or no details about equilibration (or lack of equilibration) of the nanoparticles in the exposure waters/media. |

Table S2 (continued).

|  |  | Criteria for quality rating |  |
| --- | --- | --- | --- |
| Category ^a^ | High | Medium | Low |
| **Quality of toxicity-testing and reporting of results (continued)** | | | |
| Chemistry of exposure water/medium * | At a minimum, reported:  • Temperature, pH, alkalinity, hardness, and concentrations of major inorganic ions (Ca^2+^, Mg^2+^, Na^+^, K^+^, Cl^-^, SO_4_^2-^) and dissolved or total organic carbon (DOC or TOC) in freshwater medium; or  • Temperature, pH, salinity, and DOC or TOC in saltwater medium. | At a minimum, reported:  • Temperature, pH, and hardness in freshwater medium; or  • Temperature, pH, and salinity in saltwater medium; or  • Temperature and either the name or recipe if a reconstituted exposure water/medium was used. | Did not report minimum water chemistry. |
| Measured exposure concentrations of nanoparticles | Reported measured mass concentrations of nanoparticles in the water column or at the bottom of the exposure chambers, as appropriate for the organism being tested. Measured total Ni concentrations were not an acceptable substitute for nanoparticle concentrations. | Reported measured total Ni concentrations in the water column or at the bottom of the exposure chambers. | Did not report measured nanoparticle concentrations. |
| Measured exposure concentrations of dissolved metals | Reported measured dissolved-metal concentrations in the water column or at the bottom of the exposure chambers, as appropriate for the organism being tested, in all exposure concentrations. | • Reported measured dissolved-metal concentrations in only one or a few exposure concentrations; or  • Reported measured dissolved-metal concentrations in a separate experiment but using the same water chemistry. | Did not report measured dissolved-metal concentrations. |
| Toxicity results * | • Reported detailed quantitative results for survival, growth, or reproduction endpoints in all exposure concentrations and the controls at a specified exposure time (either in a graph, a table, or the text); and  • Controls passed acceptability criteria. | • Reported detailed quantitative results for survival, growth, or reproduction endpoints in all exposure concentrations, but only as a percentage of control performance (i.e., no detailed results for controls); or  • Reported minimal quantitative results for survival, growth, or reproduction endpoints; or  • Reported summary quantitative results such as L(E)Cx concentrations or NOEC and LOEC concentrations; and  • Controls passed acceptability criteria; or  •A standardized toxicity-testing protocol that includes specific control acceptability was used, leading to a presumption that the controls passed acceptability criteria. | • Did not report quantitative results for survival, growth, or reproduction endpoints; or did not report results as mass of nanoparticles per volume of aqueous-based exposure solution (e.g., as mg/L, μg/ml, or mg/ml); or  • Controls did not pass acceptability criteria. |

Table S2 (continued).

|  |  | | Criteria for quality rating |  |
| --- | --- | --- | --- | --- |
| Category ^a^ | High | | Medium | Low |
| **Quality of nanoparticle characterization** | | | | |
| Dry size of nanoparticles * | Reported the dry size of the nanoparticles (average, range, or distribution) measured by the authors. | | Reported the dry size of the nanoparticles stated by the commercial supplier; or reported only an approximate size measured by the authors (e.g., a less-than number such as “<100 nm”). | Did not report dry size of the nanoparticles. |
| Physical-chemical characteristics of dry nanoparticles | Reported more than one characteristic of the nanoparticles (e.g., particle mass, morphology/shape, surface area, surface chemistry, crystallinity) measured by the authors. | | Only reported one characteristic of the nanoparticles measured by the authors; or  Only reported characteristics stated by the commercial supplier. | Did not report characteristics of the dry nanoparticles. |
| Physical-chemical characteristics of nanoparticles in exposure water/medium | Reported more than one characteristic of the nanoparticles (e.g., zeta potential, surface charge density, point of zero charge, hydrodynamic size, agglomeration state, polydispersity index, electrophoretic mobility, solubility in exposure water/medium) in all nanoparticle treatments, measured in the exposure water/medium by the authors. | | • Reported more than one characteristic of the nanoparticles, but not in all nanoparticle treatments; or  • Only reported one characteristic of the nanoparticles in all nanoparticle treatments, measured in the exposure water/medium by the authors; or  • Reported at least one characteristic of the nanoparticles measured in a different (or unspecified) water/medium than was used in the toxicity test (e.g., in deionized water). | Did not report characteristics of the nanoparticles in exposure water/medium or in a different water/medium than was used in the toxicity test. |
| **Overall quality rating** | | | | |
| Lowest letter rating of the following: | | • Average of all 10 rating categories, equally weighted  • Study design  • Toxicity-test methods  • Chemistry of exposure waters/media  • Toxicity results  • Dry size of nanoparticles | | |
| e.g., an “L” (the lowest rating possible) in the average of all 10 rating categories or in any of the 5 individual “prime” categories listed immediately above, results in an overall quality rating of “L” | | | | |

^a^ An asterisk indicates a prime rating category.

Table S3. Types of nanoparticulate nickel (nano-Ni) that have been used in aquatic toxicity studies. See Table S4 for details about the toxicity tests [e.g., species tested, test duration, toxicity endpoint(s)].

| Predominant chemical form | Physical form |
| --- | --- |
| Ni^0^ (elemental Ni) | Powder |
| Ni(OH)_2_ (with and without citrate coating) | Powder |
| Ni_2_O_3_ | Powder |
| NiAl_0.5_Fe_1.5_O_4_ | Powder |
| Ni/γ-Al_2_O_3_ | Nano-ceramic particles |
| NiCoLiMn-oxides (6 different stoichiometries) | Nano-sheets |
| Ni-Cu | Powder |
| Ni-Fe | Powder |
| Ni-Fe, starch-stabilized | Powder |
| NiFe_2_O_4_ | Powder |
| NiFe_2_O_4_, ABS(acrylonitrile butadiene styrene)-coated | Powder |
| NiFe_2_O_4_, ABS(acrylonitrile butadiene styrene)- and Ag-coated | Powder |
| NiFe_2_O_4_, Ag-coated | Powder |
| NiFe_2_O_4_, PRh(polyrhodanine)-coated | Powder |
| NiFe_2_O_3_Zn | Powder |
| NiFe_4_O_4_Zn | Powder |
| α-NiMoO_4_ | Nanorods |
| α-NiMoO_4_, Co-doped | Nanorods |
| α-NiMoO_4_, Cu-doped | Nanorods |
| Ni-coated Pd (palladium) | Nano-coils |
| Ni/Ag-coated Pd (palladium) | Nano-coils |
| Ni embedded SiO_2_ (silicon dioxide) | Powder or tubes |
| Ni annealed onto SiO_2_ (silicon dioxide) | Powder |
| Ni-doped TiO_2_ (titanium dioxide) | Powder |
| Ni-ZnO/rGO (nickel-zinc oxide/reduced graphene oxide composite) | Webs of Nylon 6 nano-fibers containing  Ni-ZnO/rGO |
| Ni-ZnO/rGO/Ag (nickel-zinc oxide/reduced graphene oxide composite on silver) | Webs of Nylon 6 nano-fibers containing  Ni-ZnO/rGO attached to silver (Ag) nanoparticles |
| Ni coated with exopolysaccharide | Powder |
| NiO | Powder, nanotubes, nanoflowers |
| NiO/γ-Al_2_O_3_ | Nano-ceramic particles |
| NiO/clinoptile zeolite | Powder |
| NiO-CuO | Powder |
| NiO/graphene | Nano-sheets |
| NiO-coated SiO_2_ | Powder |
| NiO-PHB[poly(3-hydroxybutyrate)] | Film |
| NiO-PHB[poly(3-hydroxybutyrate)]/ PEG(polyethylene glycol) | Film |

Table S3 (continued).

| Predominant chemical form | Physical form |
| --- | --- |
| NiO-PHB[poly(3-hydroxybutyrate)] with adsorbed norfloxacin | Film |
| NiO-PHB[poly(3-hydroxybutyrate)]/ PEG(polyethylene glycol) with adsorbed norfloxacin | Film |
| NiO infused into electrospun poly-ε-caprolactone/gelatin | Hybrid nanofibrous mat |

Table S4. Publications that report results of aquatic toxicity studies with nickel-containing nanomaterials (nano-Ni).

| Taxon | Taxon-omic group ^a^ | Form of nano-Ni ^b^ | Exposure medium ^c^ | Exposure duration ^d^ | Quality rating ^e^ | Reference |
| --- | --- | --- | --- | --- | --- | --- |
| *Acartia tonsa* | I | Ni^0^ | SW | A: 48 h C: 4&7 d | M | Zhou et al. (2016) |
|  |  | Ni^0^ | SW | C: 4 d | L | Zhou et al. (2018) |
| *Agrobacterium* sp.  PH-08 | B | Ni^0^ | FW MM | A/C: 6 h | L | Le et al. (2014) |
| *Alternaria solani* | Fu | NiFe_2_O_4_@Ag | FW A | A/C: 6 d | L | Golkhatmi et al. (2017) |
| *Anopheles subpictus* | I | Ni^0^ | FW | A: 24 h | L | Rajakumar et al. (2013) |
| *Artemia salina* | I | NiO | SW | A: 24 h C: 96 h | L | Ates et al. (2016) |
|  |  | NiO | SW | A: 24 h | L | Iqbal et al. (2019) |
|  |  | NiO | SW | A: 24 h | M | Khalil et al. (2018) |
|  |  | NiO | SW | A: 24 h | M | Nogueira et al. (2015) |
| *Aspergillus clavatus* | Fu | Ni^0^ | FW A&MM | A/C: 48 h | L | Jeyaraj Pandian et al. (2016) |
| *Aspergillus flavus* | Fu | NiO | FW A | A/C: 48 h | L | Iqbal et al. (2019) |
|  |  | NiO | FW A | A/C: 48 h | L | Khalil et al. (2018) |
| *Aspergillus fumigatus* | Fu | Ni^0^ | FW A&MM | A/C: 48 h | L | Jeyaraj Pandian et al. (2016) |
|  |  | NiO | FW A | A/C: 48 h | L | Khalil et al. (2018) |
| *Aspergillus niger* | Fu | Ni^0^ | FW A&MM | A/C: 48 h | L | Jeyaraj Pandian et al. (2016) |
|  |  | NiO | FW A | A/C: 48 h | L | Iqbal et al. (2019) |
|  |  | NiO | FW A | A/C: 48 h | L | Khalil et al. (2018) |
| *Bacillus anthracis* | B | NiO | FW MM | A/C: 20-24 h | M | Mishra et al. (2018) |
|  |  | NiO | FW A | NR | L | Rajan et al. (2017) |
| *Bacillus cereus* | B | Ni^0^ | FW A | A/C: 24 h | L | Helen and Rani (2015) |
|  |  | NiFe_2_O_4_ | FW A | NR | L | Mohebbi et al. (2019) |
|  |  | NiFe_2_O_4_ @ABS (acrylonitrile butadiene styrene) | FW A | NR | L | Mohebbi et al. (2019) |
|  |  | NiFe_2_O_4_ @ABS (acrylonitrile butadiene styrene)@Ag | FW A | NR | L | Mohebbi et al. (2019) |
|  |  | NiFe_2_O_4_ @Ag | FW A | NR | L | Mohebbi et al. (2019) |
| *Bacillus subtilis* | B | Ni^0^ | FW A | NR | L | Din et al. (2018) |
|  |  | Ni^0^ | FW A&MM | A/C: 24 h | L | Jeyaraj Pandian et al. (2016) |
|  |  | Ni^0^ | FW A | A/C: 24 h | L | Kumar et al. (2010) |
|  |  | Ni^0^ | FW A&MM | A/C: 6 h | M | Peng et al. (2018a) |
|  |  | NiFe_2_O_4_ | FW A&MM | A/C: 24 h | L | Bhushan et al. (2019) |
|  |  | NiFe_2_O_4_ | FW A&MM | A/C: 72 h | L | Udhaya et al. (2019) |
|  |  | NiFe_2_O_4_@Ag | FW A | A/C: 48 h | L | Golkhatmi et al. (2017) |
|  |  | NiZnO/rGO/ nylon-6 | FW MM | A/C: 4 h | L | Sedghi et al. (2017) |
|  |  | NiZnO/rGO/ nylon-6/Ag | FW MM | A/C: 4 h | L | Sedghi et al. (2017) |

Table S4 (continued).

| Taxon | Taxon-omic group ^a^ | Form of nano-Ni ^b^ | Exposure medium ^c^ | Exposure duration ^d^ | Quality rating ^e^ | Reference |
| --- | --- | --- | --- | --- | --- | --- |
| *Bacillus subtilis* (cont.) | B | NiO | FW A | A/C: 24 h | L | Baek and An (2011) |
|  |  | NiO | FW A&MM | A/C: 24 h | L | Bhushan et al. (2019) |
|  |  | NiO | FW A | NR | L | Din et al. (2018) |
|  |  | NiO | FW A | A/C: 24 h | L | Iqbal et al. (2019) |
|  |  | NiO | FW A | A/C: 24 h | L | Khalil et al. (2018) |
|  |  | NiO (3 morphologies) | FW MM | NR | L | Pang et al. (2009) |
|  |  | NiO | FW A | A/C: 24 h | L | Perachiselvi et al. (2018) |
|  |  | NiO | FW A | A/C: 24 h | L | Santhoshkumar et al. (2016) |
| Bacteriophage f2 | V | Ni^0^ | FW MM | A/C: 1 h | L | Cheng et al. (2019) |
|  |  | Ni-Fe_3_ | FW MM | A/C: 1 h | L | Cheng et al. (2019) |
|  |  | Ni-Fe_5_ | FW MM | A/C: 1 h | L | Cheng et al. (2019) |
|  |  | Ni-Fe_10_ | FW MM | A/C: 1 h | L | Cheng et al. (2019) |
| *Brachionus plicatilis* | I | NiO | SW | A: 48 h | M | Nogueira et al. (2015) |
| *Candida albicans* | Fu | Ni^0^ | FW A&MM | A/C: 48 h | L | Jeyaraj Pandian et al. (2016) |
|  |  | NiO | FW A | A/C: 48 h | L | Iqbal et al. (2019) |
| *Candida tropicalis* | Fu | Ni^0^ | FW A&MM | A/C: 48 h | L | Jeyaraj Pandian et al. (2016) |
| *Carassius auratus* | F | Ni-doped TiO_2_ | FW | A: 96 h C: 7 d | M | Pirsaheb et al. (2019) |
| *Ceriodaphnia dubia* | I | Ni^0^ | FW | A: 48 h | M | Griffitt et al. (2008) |
| *Chironomus riparius* | I | Ni_y_Li_x_Mn_z_Co_1-y-z_O_2_ | FW | C: 50 d | L | Niemuth et al. (2019) |
| *Chlorella vulgaris* | MicA | Ni^0^ | FW | NR | L | Morgaleva et al. (2015) |
|  |  | NiO | BW | A/C: 120 h | M | Gong et al. (2011) |
|  |  | NiO | FW | A/C: 72 h | L | Ko et al. (2018) |
|  |  | NiO | FW | A/C: 120 h | L | Li et al. (2017) |
|  |  | NiO | FW | A/C: 96 h | L | Oukarroum et al. (2017) |
|  |  | NiO + CuO (mixture) | FW | A/C: 72 h | L | Ko et al. (2018) |
|  |  | NiO + Fe_2_O_3_ (mixture) | FW | A/C: 72 h | L | Ko et al. (2018) |
|  |  | NiO + TiO_2_ (mixture) | FW | A/C: 72 h | L | Ko et al. (2018) |
|  |  | NiO + ZnO (mixture) | FW | A/C: 72 h | L | Ko et al. (2018) |
| *Ciona intestinalis* | I | Ni^0^ | SW | A: 2&24 h | M | Gallo et al. (2016) |
| *Culex gelidus* | I | Ni^0^ | FW | A: 24 h | L | Rajakumar et al. (2013) |
| *Culex quinquefasciatus* | I | Ni^0^ | FW | A: 24 h | L | Rajakumar et al. (2013) |
| *Cyprinus carpio* | F | Ni-doped TiO_2_ | FW | A: 96 h C: 7 d | M | Pirsaheb et al. (2019) |

Table S4 (continued).

| Taxon | Taxon-omic group ^a^ | Form of nano-Ni ^b^ | Exposure medium ^c^ | Exposure duration ^d^ | Quality rating ^e^ | Reference |
| --- | --- | --- | --- | --- | --- | --- |
| *Danio rerio* | F | Ni^0^ | FW | C: 84 d | L | Bagirov et al. (2019) |
|  |  | Ni^0^ | FW | A: 96 h | L | Boran and Șaffak (2018) |
|  |  | Ni^0^ | FW | A: 48 h | M | Griffitt et al. (2008) |
|  |  | Ni^0^ | FW | A: 4 d | M | Ispas et al. (2009) |
|  |  | Ni^0^ | FW | A: 96 h | M | Kovrižnych et al. (2013) |
|  |  | Ni^0^ | FW | NR | L | Morgaleva et al. (2015) |
|  |  | Ni^0^ | FW | A: 96 h | L | Morgaleva et al. (2017) |
|  |  | Ni^0^ | FW | A: 72&96 h | M | Özel et al. (2014) |
|  |  | Ni_2_O_3_ | FW | A: 120 h | M | Lin et al. (2013) |
|  |  | NiSiO_2_ | FW | A: 4 d | M | Mahoney et al. (2016) |
|  |  | NiO | FW | C: 84 d | L | Bagirov et al. (2019) |
|  |  | NiO | FW | A: 96 h | L | Hou et al. (2018a) |
|  |  | NiO | FW | A: 96 h | M | Kovrižnych et al. (2013) |
|  |  | NiO | FW | C: 30 d | M | Kovrižnych et al. (2014) |
|  |  | NiO | FW | A: 120 h | M | Lin et al. (2011) |
|  |  | NiO | FW | A: 120 h | M | Lin et al. (2013) |
|  |  | NiO | FW | A: 72 h | M | Peng et al. (2018b) |
| *Daphnia magna* | I | Ni^0^ | FW | NR | L | Morgaleva et al. (2015) |
|  |  | Ni^0^ | FW | A: 48 h | M | Oleszczuk et al. (2015) |
|  |  | NiCoLiMn-oxide | FW | A: 48 h C: 21 d | M | Bozich et al. (2017) |
|  |  | NiO | FW | A: 48 h C: 21 d | M | Gong et al. (2016) |
|  |  | NiO | FW | A: 48 h C: 21 d | M | Nogueira et al. (2015) |
| *Daphnia pulex* | I | Ni^0^ | FW | A: 48 h | M | Griffitt et al. (2008) |
| *Enterobacter aerogenes* | B | NiO | FW A | NR | L | Rajan et al. (2017) |
| *Enterobacter cloacae* | B | NiO (3 morphologies) | FW MM | NR | L | Pang et al. (2009) |
| *Enterobacter* sp. | B | NiO | FW A | A/C: 24 h | L | Perachiselvi et al. (2018) |
| *Enterococcus faecalis* | B | NiO | FW MM | A/C: 24 h | L | Kganyago et al. (2018) [taxon changed from the “*Enterobacter faecalis*” reported by the authors] ^f^ |
|  |  | NiO (3 morphologies) | FW MM | NR | L | Pang et al. (2009) [taxon changed from “*S. faecalis*” reported by the authors] ^g^ |
|  |  | NiFe_2_O_4_ | FW A | A/C: 72 h | L | Udhaya et al. (2019) |

Table S4 (continued).

| Taxon | Taxon-omic group ^a^ | Form of nano-Ni ^b^ | Exposure medium ^c^ | Exposure duration ^d^ | Quality rating ^e^ | Reference |
| --- | --- | --- | --- | --- | --- | --- |
| *Escherichia coli* | B | Ni^0^ | FW MM | A/C: 24 h | M | Argueta-Figueroa et al. (2014) |
|  |  | Ni^0^ | FW A | A/C: 24 h | L | Chaudhary et al. (2015) |
|  |  | Ni^0^ | FW A | A/C: 24 h | L | Chaudhary et al. (2019) |
|  |  | Ni^0^ | FW A | NR | L | Din et al. (2018) |
|  |  | Ni^0^ | FW A | A/C: 24 h | L | Helen and Rani (2015) |
|  |  | Ni^0^ | FW A&MM | A/C: 24 h | L | Jeyaraj Pandian et al. (2016) |
|  |  | Ni^0^ | FW A&MM | A/C: 24 h | L | Kheiri Hafshejani et al. (2018) |
|  |  | Ni^0^ | FW A | A/C: 24 h | L | Kumar et al. (2010) |
|  |  | Ni^0^ | FW A&MM | A/C: 6 h | M | Peng et al. (2018a) |
|  |  | Ni^0^ | FW MM | A/C: 2 h | M | Zhang et al. (2013) |
|  |  | NiO | FW A | A/C: 24 h | L | Baek and An (2011) |
|  |  | NiO | FW A&MM | A/C: 24 h | L | Bhushan et al. (2019) |
|  |  | NiO | FW A | NR | L | Din et al. (2018) |
|  |  | NiO | FW A | A/C: 6-7 d | L | Ezhilarasi et al. (2016) |
|  |  | NiO | FW A | A/C: 24-48 h | L | Ezhilarasi et al. (2018) |
|  |  | NiO | FW MM | A/C: 24 h | L | Horst (2009) |
|  |  | NiO | FW A | A/C: 24 h | L | Iqbal et al. (2019) |
|  |  | NiO | FW MM | A/C: 24 h | L | Kganyago et al. (2018) |
|  |  | NiO | FW A | A/C: 24 h | L | Khalil et al. (2018) |
|  |  | NiO | FW MM | A/C: 1.5 h | L | Ko and Kong (2014) |
|  |  | NiO | FW MM | A/C: 1.5 h | L | Ko et al. (2017) |
|  |  | NiO | FW MM | A/C: 8, 24,  48-96 h | M | Paul and Neogi (2019) |
|  |  | NiO | FW A | A/C: 24 h | L | Perachiselvi et al. (2018) |
|  |  | NiO | FW A&MM | A/C: 24 h | L | Rajivgandhi et al. (2019) |
|  |  | NiO | FW A | NR | L | Sabouri et al. (2019) |
|  |  | NiO | FW A | A/C: 24 h | L | Santhoshkumar et al. (2016) |
|  |  | NiO | FW MM | A/C: 8 h | L | Wang et al. (2010) |
|  |  | Ni(OH)_2_ | FW A&MM | A/C: 24 h | L | Kheiri Hafshejani et al. (2018) |
|  |  | NiAl_0.5_Fe_1.5_O_4_ | FW A | A/C: 24 h | L | Naik et al. (2018) |
|  |  | Ni-Cu | FW MM | A/C: 24 h | M | Argueta-Figueroa et al. (2014) |
|  |  | Ni-Fe (1% Ni by wt) | FW MM | A/C: 1 h | L | Dong et al. (2019) |
|  |  | Ni-Fe (3% Ni by wt) | FW MM | A/C: 1 h | L | Dong et al. (2019) |
|  |  | Ni-Fe (5% Ni by wt) | FW MM | A/C: 1 h | L | Dong et al. (2019) |
|  |  | Ni-Fe, starch-stabilized (5% Ni & 0.4% starch by wt) | FW MM | A/C: 1 h | L | Dong et al. (2019) |
|  |  | Ni-Fe, starch-stabilized (5% Ni & 2% starch by wt) | FW MM | A/C: 1 h | L | Dong et al. (2019) |
|  |  | Ni-Fe, starch-stabilized (5% Ni & 4% starch by wt) | FW MM | A/C: 1 h | L | Dong et al. (2019) |

Table S4 (continued).

| Taxon | Taxon-omic group ^a^ | Form of nano-Ni ^b^ | Exposure medium ^c^ | Exposure duration ^d^ | Quality rating ^e^ | Reference |
| --- | --- | --- | --- | --- | --- | --- |
| *Escherichia coli* (cont.) | B | NiFe_2_O_4_ | FW A&MM | A/C: 24 h | L | Bhushan et al. (2019) |
|  |  | NiFe_2_O_4_ | FW A | A/C: 1 d | L | Lashkenari et al. (2019) |
|  |  | NiFe_2_O_4_ | FW A | NR | L | Mohebbi et al. (2019) |
|  |  | NiFe_2_O_4_ | FW A | A/C: 24 h | L | Naik et al. (2018) |
|  |  | NiFe_2_O_4_ | FW A | NR | L | Udhaya et al. (2019) |
|  |  | NiFe_2_O_4_ @ABS (acrylonitrile butadiene styrene) | FW A | NR | L | Mohebbi et al. (2019) |
|  |  | NiFe_2_O_4_ @ABS (acrylonitrile butadiene styrene)@Ag | FW A | NR | L | Mohebbi et al. (2019) |
|  |  | NiFe_2_O_4_ @Ag | FW A | NR | L | Mohebbi et al. (2019) |
|  |  | NiFe_2_O_4_ @PRh (polyrhodanine) | FW A&MM | A/C: 1 d | L | Lashkenari et al. (2019) |
|  |  | Ni-coated Pd | FW MM | A/C: 6 h | L | Hoop et al. (2016) |
|  |  | Ni/Ag-coated Pd | FW MM | A/C: 6 h | L | Hoop et al. (2016) |
|  |  | Ni-doped TiO_2_ | FW MM | A/C: 24 h | L | Ananpattarachai et al. (2016) |
|  |  | NiZnO/rGO/nylon-6 | FW MM | A/C: 4 h | L | Sedghi et al. (2017) |
|  |  | NiZnO/rGO/nylon-6/Ag | FW MM | A/C: 4 h | L | Sedghi et al. (2017) |
|  |  | NiO/clinoptile | FW | A/C: 24 h | L | Hrenovic et al. (2012) |
|  |  | NiO-CuO | FW MM | A/C: 8, 24,  48-96 h | M | Paul and Neogi (2019) |
|  |  | NiO/graphene | FW A&MM | A/C: 24 h | L | Rajivgandhi et al. (2019) |
|  |  | NiO & PHB [poly(3-hydroxybutyrate)] nanocomposite | FW A | A/C: 24 h | L | Abdelwahab et al. (2018) |
|  |  | NiO (3%) & PHB [poly(3-hydroxy-butyrate)]/PEG (polyethylene glycol) nanocomposite | FW A | A/C: 24 h | L | Abdelwahab et al. (2018) |
|  |  | NiO (5%) & PHB [poly(3-hydroxy-butyrate)]/PEG (polyethylene glycol) nanocomposite | FW A | A/C: 24 h | L | Abdelwahab et al. (2018) |
|  |  | NiO & PHB [poly(3-hydroxybutyrate)] nanocomposite with adsorbed norfloxacin (an antibiotic) | FW A | A/C: 24 h | L | Abdelwahab et al. (2018) |

Table S4 (continued).

| Taxon | Taxon-omic group ^a^ | Form of nano-Ni ^b^ | Exposure medium ^c^ | Exposure duration ^d^ | Quality rating ^e^ | Reference |
| --- | --- | --- | --- | --- | --- | --- |
| *Escherichia coli* (cont.) | B | NiO (3%) & PHB [poly(3-hydroxy-butyrate)]/PEG (polyethylene glycol) nanocomposite with adsorbed norfloxacin (an antibiotic) | FW A | A/C: 24 h | L | Abdelwahab et al. (2018) |
|  |  | NiO (5%) & PHB [poly(3-hydroxy-butyrate)]/PEG (polyethylene glycol) nanocomposite with adsorbed norfloxacin (an antibiotic) | FW A | A/C: 24 h | L | Abdelwahab et al. (2018) |
|  |  | NiO infused into electrospun poly-ε-caprolactone/gelatin hybrid nanofibrous mat | FW A&MM | A/C: 24 h | L | Ramalingam et al. (2019) |
|  |  | NiO + Co_3_O_4_ (mixture) | FW MM | A/C: 1.5 h | L | Ko et al. (2017) |
|  |  | NiO + CuO (mixture) | FW MM | A/C: 1.5 h | L | Ko et al. (2017) |
|  |  | NiO + ZnO (mixture) | FW MM | A/C: 1.5 h | L | Ko et al. (2017) |
| *Escherichia hermannii* | B | NiO | FW A | A/C: 6-7 d | L | Ezhilarasi et al. (2016) |
|  |  | NiO | FW A | A/C: 24-48 h | L | Ezhilarasi et al. (2018) |
| *Euplotes affinis* | Pr | NiO/clinoptile | FW | A: 24 h | L | Hrenovic et al. (2012) |
| *Fusarium oxysporum* | Fu | NiFe_2_O_4_@Ag | FW A | A/C: 6 d | L | Golkhatmi et al. (2017) |
| *Fusarium solani* | Fu | NiO | FW A | A/C: 48 h | L | Iqbal et al. (2019) |
| *Gracilaria lemaneiformis* | MacA | NiO | SW | A: 48 h | L | Han et al. (2012) |
| *Heterocypris incongruens* | I | Ni^0^ | S | C: 6 d | L | Jośko et al. (2016) |
| *Hydra attenuata* | I | NiFe_2_O_3_Zn | FW | C: 96 h | L | Blaise et al. (2008) |
| *Klebsiella pneumoniae* | B | Ni^0^ | FW A | A/C: 24 h | L | Chaudhary et al. (2019) |
|  |  | Ni^0^ | FW A | A/C: 24 h | L | Helen and Rani (2015) |
|  |  | Ni^0^ | FW A&MM | A/C: 24 h | L | Jeyaraj Pandian et al. (2016) |
|  |  | Ni^0^ | FW A&MM | A/C: 24 h | L | Kheiri Hafshejani et al. (2018) |
|  |  | Ni^0^ | FW A | A/C: 18 h | L | Sudhasree et al. (2014) |
|  |  | NiO | FW A | A/C: 24 h | L | Iqbal et al. (2019) |
|  |  | NiO | FW A | A/C: 24 h | L | Khalil et al. (2018) |
|  |  | NiO | FW A | NR | L | Rajan et al. (2017) |
|  |  | Ni(OH)_2_ | FW A&MM | A/C: 24 h | L | Kheiri Hafshejani et al. (2018) |

Table S4 (continued).

| Taxon | Taxon-omic group ^a^ | Form of nano-Ni ^b^ | Exposure medium ^c^ | Exposure duration ^d^ | Quality rating ^e^ | Reference |
| --- | --- | --- | --- | --- | --- | --- |
| *Klebsiella pneumoniae* (cont.) | B | NiO & PHB [poly(3-hydroxybutyrate)] nanocomposite | FW A | A/C: 24 h | L | Abdelwahab et al. (2018) |
|  |  | NiO (3%) & PHB [poly(3-hydroxy-butyrate)]/PEG (polyethylene glycol) nanocomposite | FW A | A/C: 24 h | L | Abdelwahab et al. (2018) |
|  |  | NiO (5%) & PHB [poly(3-hydroxy-butyrate)]/PEG (polyethylene glycol) nanocomposite | FW A | A/C: 24 h | L | Abdelwahab et al. (2018) |
|  |  | NiO & PHB [poly(3-hydroxybutyrate)] nanocomposite with adsorbed norfloxacin (an antibiotic) | FW A | A/C: 24 h | L | Abdelwahab et al. (2018) |
|  |  | NiO (3%) & PHB [poly(3-hydroxy-butyrate)]/PEG (polyethylene glycol) nanocomposite with adsorbed norfloxacin (an antibiotic) | FW A | A/C: 24 h | L | Abdelwahab et al. (2018) |
|  |  | NiO (5%) & PHB [poly(3-hydroxy-butyrate)]/PEG (polyethylene glycol) nanocomposite with adsorbed norfloxacin (an antibiotic) | FW A | A/C: 24 h | L | Abdelwahab et al. (2018) |
| *Klebsiella* sp. | B | Ni^0^ | FW A | A/C: 24 h | L | Chaudhary et al. (2015) |
| *Labeo rohita* | F | Ni^0^ | FW | C: 21 d | L | Kanwal et al. (2019) |
| *Lactobacillus* sp. | B | Ni^0^ | FW A | A/C: 24 h | L | Kumar et al. (2010) |
| *Lemna gibba* | P | NiO | FW | A: 24 h | L | Oukarroum et al. (2015) |
| *Lemna minor* | P | NiO | FW | C: 7 d | M | Nogueira et al. (2015) |
| *Lepomis macrochirus* | F | Ni^0^ | FW | A: 24 h | L | Poornavaishnavi et al. (2019) |
| *Leptocheirus plumulosus* | I | NiO | SW | C: 10 d | L | Hanna et al. (2013) |
| MARA ^h^ | B | NiFe_2_O_3_Zn | FW MM | A/C: 18 h | L | Blaise et al. (2008) |
| *Mucor racemosus* | Fu | NiO | FW A | A/C: 48 h | L | Iqbal et al. (2019) |
|  |  | NiO | FW A | A/C: 48 h | L | Khalil et al. (2018) |
| *Oncorhynchus* | F | NiO | FW | C: 60 d | L | Nazdar et al. (2018) |
| mykiss |  | NiFe_2_O_3_Zn | FW | A: 48 h | L | Blaise et al. (2008) ^i^ |

Table S4 (continued).

| Taxon | Taxon-omic group ^a^ | Form of nano-Ni ^b^ | Exposure medium ^c^ | Exposure duration ^d^ | Quality rating ^e^ | Reference |  |
| --- | --- | --- | --- | --- | --- | --- | --- |
| *Oreochromis mossambicus* | F | Ni^0^ | FW | C: 14 d | L | Jayaseelan et al. (2014) |  |
| *Oryzias latipes* | F | NiO | FW | NR | L | Lin et al. (2013) |  |
| *Paracentrotus lividus* | I | Ni^0^ | SW | A: 46 h | M | Kanold et al. (2016) |  |
| *Paramecium* | Pr | Ni^0^ | FW MM | NR | L | Morgaleva et al. (2015) |  |
| *caudatum* |  | NiO/clinoptile | FW | A: 24 h | L | Hrenovic et al. (2012) |  |
| *Photobacterium phosphoreum* | B | NiO | SW MM | A/C: 0.25 h | L | Wang et al. (2016) |  |
| *Pneumonia typhus* | B | Ni^0^ | FW A | A/C: 24 h | L | Chaudhary et al. (2019) |  |
| *Proteus vulgaris* | B | Ni^0^ | FW A | A/C: 18 h | L | Sudhasree et al. (2014) |  |
|  |  | NiO | FW A | A/C: 24 h | L | Santhoshkumar et al. (2016) |  |
| *Pseudokirchneriella* | MicA | Ni^0^ | FW | A/C: 96 h | M | Griffitt et al. (2008) |  |
| *subcapitata* |  | NiO | FW | A/C: 72 h | M | Nogueira et al. (2015) |  |
|  |  | NiO | FW | A/C: 72 h | M | Sousa et al. (2018b) |  |
|  |  | NiFe_2_O_3_Zn | FW | A/C: 72 h | L | Blaise et al. (2008) |  |
| *Pseudomonas* | B | Ni^0^ | FW A | A/C: 24 h | L | Chaudhary et al. (2015) |  |
| *aeruginosa* |  | Ni^0^ | NR | NR | L | Din et al. (2018) |  |
|  |  | Ni^0^ | FW A | A/C: 24 h | L | Kumar et al. (2010) |  |
|  |  | Ni^0^ | FW A | A/C: 18 h | L | Sudhasree et al. (2014) |  |
|  |  | NiO | NR | NR | L | Din et al. (2018) |  |
|  |  | NiO | FW A | A/C: 24 h | L | Iqbal et al. (2019) |  |
|  |  | NiO | FW MM | A/C: 24 h | L | Kganyago et al. (2018) |  |
|  |  | NiO | FW A | A/C: 24 h | L | Khalil et al. (2018) |  |
|  |  | NiO (3 morphologies) | FW MM | NR | L | Pang et al. (2009) |  |
|  |  | NiO | FW A&MM | A/C: 24 h | L | Rajivgandhi et al. (2019) |  |
|  |  | NiO | FW A | NR | L | Sabouri et al. (2019) |  |
|  |  | NiAl_0.5_Fe_1.5_O_4_ | FW A | A/C: 24 h | L | Naik et al. (2018) | |
|  |  | NiFe_2_O_4_ | FW A | A/C: 24 h | L | Naik et al. (2018) | |
|  |  | NiFe_2_O_4_ | FW A&MM | A/C: 72 h | L | Udhaya et al. (2019) | |
|  |  | NiFe_2_O_4_@Ag | FW A | A/C: 48 h | L | Golkhatmi et al. (2017) | |
|  |  | α-NiMoO_4_ | FW MM | A/C: 3 h | M | Ray et al. (2018) | |
|  |  | α-NiMoO_4_, Co-doped | FW MM | A/C: 3 h | M | Ray et al. (2018) | |
|  |  | α-NiMoO_4_, Cu-doped | FW MM | A/C: 3 h | M | Ray et al. (2018) | |
|  |  | Ni with exopolysaccharide coating | FW MM | A/C: 20 h | L | Garza-Cervantes et al. (2019) | |
|  |  | NiO/graphene | FW A&MM | A/C: 24 h | L | Rajivgandhi et al. (2019) | |
|  |  | NiO infused into electrospun poly-ε-caprolactone/gelatin hybrid nanofibrous mat | FW A&MM | A/C: 24 h | L | Ramalingam et al. (2019) | |

Table S4 (continued).

| Taxon | Taxon-omic group ^a^ | Form of nano-Ni ^b^ | Exposure medium ^c^ | Exposure duration ^d^ | Quality rating ^e^ | Reference |
| --- | --- | --- | --- | --- | --- | --- |
| *Rhinella arenarum* | Amph | Ni/γ-Al_2_O_3_ | FW | A: 96 h C: 168& 504 h | M | Svartz et al. (2017) |
|  |  | Ni/γ-Al_2_O_3_ | FW | A: 96 h C: 168& 336 h | M | Svartz et al. (2019) |
|  |  | NiO/γ-Al_2_O_3_ | FW | A: 96 h C: 168& 336 h | M | Svartz et al. (2019) |
| *Rhizoctonia solani* | Fu | NiO | FW A | A/C: 48 h | L | Khalil et al. (2018) |
| *Saccharomyces* | Y | NiO | FW MM | A/C: 6&24 h | M | Sousa et al. (2018a) |
| *cerevisiae* |  | NiO | FW MM | A/C: 6 h | M | Sousa et al. (2018c) |
|  |  | NiO | FW MM | A/C: 6&<48 h | M | Sousa et al. (2019) |
| *Salmonella typhi* | B | Ni^0^ | FW A&MM | A/C: 24 h | L | Jeyaraj Pandian et al. (2016) |
|  |  | NiO | FW A&MM | A/C: 24 h | L | Bhushan et al. (2019) |
|  |  | NiFe_2_O_4_ | FW A&MM | A/C: 24 h | L | Bhushan et al. (2019) |
| *Salmonella* | B | NiO | FW A | A/C: 48 h | L | Nogueira et al. (2015) |
| *typhimurium* |  | NiAl_0.5_Fe_1.5_O_4_ | FW A | A/C: 24 h | L | Naik et al. (2018) |
|  |  | NiFe_2_O_4_ | FW A | A/C: 24 h | L | Naik et al. (2018) |
|  |  | NiFe_2_O_4_ | FW A | NR | L | Mohebbi et al. (2019) |
|  |  | NiFe_2_O_4_ @ABS (acrylonitrile butadiene styrene) | FW A | NR | L | Mohebbi et al. (2019) |
|  |  | NiFe_2_O_4_ @ABS (acrylonitrile butadiene styrene)@Ag | FW A | NR | L | Mohebbi et al. (2019) |
|  |  | NiFe_2_O_4_ @Ag | FW A | NR | L | Mohebbi et al. (2019) |
| *Shewanella oneidensis* | B | NiCoLiMn-oxide (3) | FW MM | A/C: 72 h | L | Gunsolus et al. (2017) |
| MR-1 |  | NiCoLiMn-oxide | FW MM | A/C: 24& 60 h | L | Hang et al. (2016) |
|  |  | NiCoLiMn-oxide (3) | FW MM | NR | L | Hang et al. (2018) |

Table S4 (continued).

| Taxon | Taxon-omic group ^a^ | Form of nano-Ni ^b^ | Exposure medium ^c^ | Exposure duration ^d^ | Quality rating ^e^ | Reference |
| --- | --- | --- | --- | --- | --- | --- |
| *Staphylococcus aureus* | B | Ni^0^ | FW MM | A/C: 24 h | M | Argueta-Figueroa et al. (2014) |
|  |  | Ni^0^ | FW A | A/C: 24 h | L | Chaudhary et al. (2015) |
|  |  | Ni^0^ | FW A | A/C: 24 h | L | Helen and Rani (2015) |
|  |  | Ni^0^ | FW A&MM | A/C: 24 h | L | Kheiri Hafshejani et al. (2018) |
|  |  | Ni^0^ | FW A | A/C: 24 h | L | Kumar et al. (2010) |
|  |  | Ni^0^ | FW A | A/C: 18 h | L | Sudhasree et al. (2014) |
|  |  | NiO | FW A | A/C: 24 h | L | Baek and An (2011) [taxon changed from the “*Streptococcus aureus*” reported by the authors] ^j^ |
|  |  | NiO | FW A&MM | A/C: 24 h | L | Bhushan et al. (2019) |
|  |  | NiO | FW A | A/C: 6-7 d | L | Ezhilarasi et al. (2016) |
|  |  | NiO | FW A | A/C: 24-48 h | L | Ezhilarasi et al. (2018) |
|  |  | NiO | FW A | A/C: 3 h | L | Hou et al. (2018b) |
|  |  | NiO | FW A | A/C: 24 h | L | Iqbal et al. (2019) |
|  |  | NiO | FW MM | A/C: 24 h | L | Kganyago et al. (2018) |
|  |  | NiO | FW A | A/C: 24 h | L | Khalil et al. (2018) |
|  |  | NiO (3 morphologies) | FW MM | NR | L | Pang et al. (2009) |
|  |  | NiO | FW MM | A/C: 8, 24,  48-96 h | M | Paul and Neogi (2019) |
|  |  | NiO | FW A | NR | L | Sabouri et al. (2019) |
|  |  | NiO | FW A | A/C: 24 h | L | Santhoshkumar et al. (2016) |
|  |  | Ni(OH)_2_ | FW A&MM | A/C: 24 h | L | Kheiri Hafshejani et al. (2018) |
|  |  | Ni-Cu | FW MM | A/C: 24 h | M | Argueta-Figueroa et al. (2014) |
|  |  | NiFe_2_O_4_ | FW A&MM | A/C: 24 h | L | Bhushan et al. (2019) |
|  |  | NiFe_2_O_4_ | FW A | A/C: 1 d | L | Lashkenari et al. (2019) |
|  |  | NiFe_2_O_4_ | FW A | NR | L | Mohebbi et al. (2019) |
|  |  | NiFe_2_O_4_ @ABS (acrylonitrile butadiene styrene) | FW A | NR | L | Mohebbi et al. (2019) |
|  |  | NiFe_2_O_4_ @ABS (acrylonitrile butadiene styrene)@Ag | FW A | NR | L | Mohebbi et al. (2019) |
|  |  | NiFe_2_O_4_ @Ag | FW A | NR | L | Mohebbi et al. (2019) |
|  |  | NiFe_2_O_4_ @PRh (polyrhodanine) | FW A&MM | A/C: 1 d | L | Lashkenari et al. (2019) |
|  |  | Ni/Ag-coated Pd | FW MM | A/C: 6 h | L | Hoop et al. (2016) |
|  |  | Ni-doped TiO2 | FW MM | A/C: 24 h | L | Ananpattarachai et al. (2016) |
|  |  | Ni with exopolysaccharide coating | FW MM | A/C: 20 h | L | Garza-Cervantes et al. (2019) |
|  |  | NiO/clinoptile | FW | A/C: 24 h | L | Hrenovic et al. (2012) |

Table S4 (continued).

| Taxon | Taxon-omic group ^a^ | Form of nano-Ni ^b^ | Exposure medium ^c^ | Exposure duration ^d^ | Quality rating ^e^ | Reference |
| --- | --- | --- | --- | --- | --- | --- |
| *Staphylococcus aureus* (cont.) | B | NiO-CuO | FW MM | A/C: 8, 24,  48-96 h | M | Paul and Neogi (2019) |
|  |  | NiO-coated SiO_2_ | FW A | A/C: 3 h | L | Hou et al. (2018b) |
|  |  | NiO & PHB [poly(3-hydroxybutyrate)] nanocomposite | FW A | A/C: 24 h | L | Abdelwahab et al. (2018) |
|  |  | NiO (3%) & PHB [poly(3-hydroxy-butyrate)]/PEG (polyethylene glycol) nanocomposite | FW A | A/C: 24 h | L | Abdelwahab et al. (2018) |
|  |  | NiO (5%) & PHB [poly(3-hydroxy-butyrate)]/PEG (polyethylene glycol) nanocomposite | FW A | A/C: 24 h | L | Abdelwahab et al. (2018) |
|  |  | NiO & PHB [poly(3-hydroxybutyrate)] nanocomposite with adsorbed norfloxacin (an antibiotic) | FW A | A/C: 24 h | L | Abdelwahab et al. (2018) |
|  |  | NiO (3%) & PHB [poly(3-hydroxy-butyrate)]/PEG (polyethylene glycol) nanocomposite with adsorbed norfloxacin (an antibiotic) | FW A | A/C: 24 h | L | Abdelwahab et al. (2018) |
|  |  | NiO (5%) & PHB [poly(3-hydroxy-butyrate)]/PEG (polyethylene glycol) nanocomposite with adsorbed norfloxacin (an antibiotic) | FW A | A/C: 24 h | L | Abdelwahab et al. (2018) |
|  |  | NiO infused into electrospun poly-ε-caprolactone/gelatin hybrid nanofibrous mat | FW A&MM | A/C: 24 h | L | Ramalingam et al. (2019) |
| *Staphylococcus* | B | Ni^0^ | FW A&MM | A/C: 24 h | L | Jeyaraj Pandian et al. (2016) |
| *epidermidis* |  | NiO | FW A | A/C: 24 h | L | Khalil et al. (2018) |
| *Streptococcus mutans* | B | Ni^0^ | FW MM | A/C: 24 h | M | Argueta-Figueroa et al. (2014) |
|  |  | Ni-Cu | FW MM | A/C: 24 h | M | Argueta-Figueroa et al. (2014) |
| *Streptococcus pneumoniae* | B | NiO | FW A | A/C: 6-7 d | L | Ezhilarasi et al. (2016) [taxon changed from the “*Staphylococcus pneumoniae*” reported by the authors] ^k^ |
|  |  | NiO | FW A | A/C: 24-48 h | L | Ezhilarasi et al. (2018) |
|  |  | NiO | FW A | NR | L | Rajan et al. (2017) |

Table S4 (continued).

| Taxon | Taxon-omic group ^a^ | Form of nano-Ni ^b^ | Exposure medium ^c^ | Exposure duration ^d^ | Quality rating ^e^ | Reference |
| --- | --- | --- | --- | --- | --- | --- |
| *Thamnocephalus platyurus* | I | NiFe_2_O_3_Zn | FW | A: 24 h | L | Blaise et al. (2008) |
| *Vibrio cholerae* | B | Ni^0^ | FW A | A/C: 18 h | L | Sudhasree et al. (2014) |
| *Vibrio fischeri* | B | Ni^0^ | SW MM | A/C: 0.25 h | L | Jośko et al. (2016) |
|  |  | NiO | SW MM | A/C: 0.5 h | L | Nogueira et al. (2015) |
|  |  | Ni/γ-Al_2_O_3_ | SW MM | A/C: 80 min | L | Svartz et al. (2017) |
|  |  | NiFe_2_O_3_Zn | SW MM | A/C: 0.25 h | L | Blaise et al. (2008) |
| Anaerobic microbe | B | Ni^0^ | FW | A/C: 54 d | L | He et al. (2019a) |
| assemblage |  | Ni^0^ | FW | A/C: 28 d | L | He et al. (2019b) |
|  |  | NiO | FW | A/C: 140 d | L | Xu et al. (2019) |
|  |  | NiFe_2_O_4_ | FW | A/C: 7 d | L | Chen et al. (2018) |
|  |  | NiFe_4_O_4_Zn | FW | A/C: 7 d | L | Chen et al. (2018) |

^a^ Amph = amphibian; B = bacteria; F = fish; Fu = fungus; I = invertebrate; MacA = macroalga; MicA = microalga; P = plant; Pr = protozoa; V = virus; Y = yeast.

^b^ Ni^0^ = elemental nickel; Ni/γ-Al_2_O_3_ = a nano-ceramic material; NiO = nickel oxide; NiO/clinoptile = a nano-zeolite; NiFe_2_O_4_@Ag = silver attached to iron-nickel-oxide; NiZnO/rGO/nylon-6 = webs of Nylon 6 nano-fibers containing nickel-zinc oxide/reduced graphene oxide composite; NiZnO/rGO/nylon-6/Ag = webs of Nylon 6 nano-fibers containing nickel-zinc oxide/reduced graphene oxide composite attached to silver nanoparticles.

^c^ A = agar; BW = brackish-water-based; F = food; FW = freshwater; G = gavage; IP = intraperitoneal; MM = microbial medium; NR = not reported; S = sediment/soil; SW = saltwater; UNK = unknown because article is in Czechoslovakian.

^d^ A = acute (short-term); C = chronic (long-term); NR = not reported; UNK = unknown because article is written in Czechoslovakian.

^e^ H = high; L = low; M = medium.

^f^ Kganyago et al. (2018) reported that they conducted a toxicity test with *Enterobacter faecalis*. However, no bacterial species of that name exists. Thus, we have assumed they conducted the test with *Enterococcus faecalis*.

^g^ Pang et al. (2009) reported that they conducted a toxicity test with *S. faecalis*. However, the bacteria formerly called *Streptococcus faecalis* has been taxonomically reassigned to the genus *Enterococcus*.

^h^ MARA = microbial assay for risk assessment.

^i^ Only hepatocytes tested (not entire organism).

^j^ Baek and An (2011) reported that they conducted a toxicity test with *Streptococcus aureus*. However, no bacterial species of that name exists. Thus, based on references in their article, we have assumed they conducted the test with *Staphylococcus aureus*.

^k^ Ezhilarasi et al. (2016) reported a result for *Staphylococcus pneumoniae* in their Figure 8. However, no bacterial species of that name exists. Thus, as indicated in the text of that article, we have assumed they conducted the toxicity test with *Streptococcus pneumoniae*.

Table S5. Median lethal (or effect) concentrations [L(E)C50s] for freshwater and saltwater organisms exposed to nanoparticulate elemental nickel (nano-Ni^0^) or nanoparticulate nickel oxide (nano-NiO) in aqueous-based medium. NR = not reported. Green-highlighted studies received a low quality rating and were not used in the meta-analysis.

| Taxon and endpoint | Nano-metal | Water ^a^ | Individual L(E)C50s (mg Ni/L) |
| --- | --- | --- | --- |
| *Acartia tonsa* (calanoid copepod) [48-h naupliar mortality] | Ni^0^ | SW | 20.2 (Zhou et al. 2016) 22.1 (Zhou et al. 2016) |
| *Agrobacterium* sp. PH-08 (bacteria) [6-h population growth] | Ni^0^ | FW | 163 (Le et al. 2014) |
| Anaerobic microbial assemblage (bacteria) [15- to 30-d nitrogen removal] | NiO | FW | >7.88 (Xu et al. 2019) |
| *Anopheles subpictus* (mosquito) [24-h survival of 4^th^ instar larvae] | Ni^0^ | FW | 4.32 (Rajakumar et al. 2013) |
| *Artemia salina* (brine shrimp) [24-h survival of larvae] | NiO | SW | >15.8 (Nogueira et al. 2015)  32.2 (Iqbal et al. 2019) 33.6 (Khalil et al. 2018) |
| *Aspergillus clavatus* (fungus) [48-h population growth] | Ni^0^ | FW | 32.6 ^b^ (Jeyaraj Pandian et al. 2016) |
| *Aspergillus fumigatus* (fungus) [48-h population growth] | Ni^0^ | FW | 34.3 ^b^ (Jeyaraj Pandian et al. 2016) |
| *Aspergillus niger* (fungus) [48-h population growth] | Ni^0^ | FW | 32.2 ^b^ (Jeyaraj Pandian et al. 2016) |
| *Bacillus anthracis* (bacteria) [20- to 24-h population growth] | NiO | FW | 2.90 (Mishra et al. 2018) |
| *Bacillus subtilis* (bacteria) [6- or 24-h population growth] | Ni^0^ & NiO | FW | <20 (Peng et al. 2018a) [Ni^0^, 6-h] 33.7 ^b^ (Jeyaraj Pandian et al. 2016) [Ni^0^, 24-h] 802 (Bhushan et al. 2019) [NiO, 24-h] |
| Bacteriophage f2 (virus) [1-h population growth] | Ni^0^ | FW | <40 (Cheng et al. 2019) |
| *Brachionus plicatilis* (saltwater rotifer) [48-h survival of neonates] | NiO | SW | >15.8 (Nogueira et al. 2015) |
| *Candida albicans* (fungus) [48-h population growth] | Ni^0^ | FW | 34.5 ^b^ (Jeyaraj Pandian et al. 2016) |
| *Candida tropicalis* (fungus) [48-h population growth] | Ni^0^ | FW | 29.8 ^b^ (Jeyaraj Pandian et al. 2016) |
| *Ceriodaphnia dubia* (water flea) [48-h survival of neonates] | Ni^0^ | FW | 0.674 (Griffitt et al. 2008) |
| *Chlorella vulgaris* (green alga) [72- or 96-h population growth,  or exposure time not reported] | Ni^0^ & NiO | FW & BW | 0.125 (Oukarroum et al. 2017) [NiO, 96-h] 0.529 (Morgaleva et al. 2015) [Ni^0^, NR] 19.9 (Ko et al. 2018) [NiO, 72-h] 20.4 (Li et al. 2017) [NiO, 72-h] 30.4 (Gong et al. 2011) [NiO, 72-h] |
| *Ciona intestinalis* (sea squirt; ascidian) [24-h morphological development of larvae] ^c^ | Ni^0^ | SW | 16.8 (Gallo et al. 2016) |

Table S5 (continued).

| Taxon and endpoint | Nano-metal | Water ^a^ | Individual L(E)C50s (mg Ni/L) |
| --- | --- | --- | --- |
| *Culex gelidus* (mosquito) [24-h survival of 4^th^ instar larvae] | Ni^0^ | FW | 4.64 (Rajakumar et al. 2013) |
| *Culex quinquefasciatus* (mosquito) [24-h survival of 4^th^ instar larvae] | Ni^0^ | FW | 4.48 (Rajakumar et al. 2013) |
| *Danio rerio* (zebrafish) [67- to 120-h hatching of embryos (H);  or 48- or 96-h survival (S),  or exposure time not reported] ^d^ | Ni^0^ & NiO | FW | >10 (Griffitt et al. 2008) [Ni^0^, 48-h, S] 30.8 (Özel et al. 2014) [Ni^0^, H] <39.4 (Lin et al. 2011) [NiO, H] <39.4 (Lin et al. 2013) [NiO, H] <39.4 (Peng et al. 2018b) [NiO, H] >100 (Morgaleva et al. 2015) [Ni^0^, NR, S] >100 (Morgaleva et al. 2017) [Ni^0^, 96-h, S] 115 (Ispas et al. 2009) [Ni^0^, 96-h, S] 122 (Boran and Șaffak 2018) [Ni^0^, 96-h, S] 124 (Hou et al. 2018) [NiO, 96-h, S] 221 (Ispas et al. 2009) [Ni^0^, 96-h, S] 328 (Ispas et al. 2009) [Ni^0^, 96-h, S] 361 (Ispas et al. 2009) [Ni^0^, 96-h, S] 331 (Kovrižnych et al. 2013) [ Ni^0^, 96-h, S] >400 (Kovrižnych et al. 2013) [Ni^0^, 96-h, S] |
| *Daphnia magna* (water flea) [48-h survival of neonates,  or exposure time not reported] | Ni^0^ & NiO | FW | 5.68 (Oleszczuk et al. 2015) [Ni^0^, 48-h] 7.67 (Nogueira et al. 2015) [NiO, 48-h] 7.69 (Nogueira et al. 2015) [NiO, 48-h] 29.0 (Gong et al. 2016) [NiO, 48-h] >100 (Morgaleva et al. 2015) [Ni^0^, NR] |
| *Daphnia pulex* (water flea) [48-h survival of adults] | Ni^0^ | FW | 3.89 (Griffitt et al. 2008) |
| *Enterococcus faecalis* (bacteria) [24-h population growth] | NiO | FW | >39.4 (Kganyago et al. 2018) |
| *Escherichia coli* (bacteria) [6-, 8-, or 24-h population growth (G), or 1.5-h bioluminescence (B)] ^e^ | Ni^0^ & NiO | FW | 1.34 (Paul & Neogi 2019) [NiO, 24-h, G] 3.46 (Paul & Neogi 2019) [NiO, 8-h, G] <3.94 (Kganyago et al. 2018) [NiO, 24-h, G] 6.03 (Argueta-Figueroa et al. 2014) [Ni^0^, 24-h, G] <10 (Zhang et al. 2013) [Ni^0^, 24-h, G] 15.6 (Rajivgandhi et al. 2019) [NiO, 24-h, G] <20 (Peng et al. 2018a) [Ni^0^, 6-h, G] 31.5 ^b^ (Jeyaraj Pandian et al. 2016) [Ni^0^, 24-h, G] 121 (Wang et al. 2010) [NiO, 8-h, G] 198 (Ko & Kong 2014) [NiO, 1.5-h, B] 810 (Bhushan et al. 2019) [NiO, 24-h, G] |
| *Gracilaria lemaneiformis* (red alga) [48-h survival] | NiO | SW | 1.63 (Han et al. 2012) |
| *Klebsiella pneumoniae* (bacteria) [24-h population growth] | Ni^0^ & NiO | FW | 18.6 (Rajivgandhi et al. 2019) [NiO] 34.3 ^b^ (Jeyaraj Pandian et al. 2016) [Ni^0^] |
| *Lemna gibba* (gibbous duckweed) [24-h photosynthesis] ^f^ | NiO | FW | 249 ^b^ (Oukarroum et al. 2015) |

Table S5 (continued).

| Taxon and endpoint | Nano-metal | Water ^a^ | Individual L(E)C50s (mg Ni/L) |
| --- | --- | --- | --- |
| *Lepomis macrochirus* (bluegill sunfish) [24-h viability of BF-2 cells] | Ni^0^ | FW | 31.4 (Poornavaishnavi et al. 2019) |
| *Paracentrotus lividus* (purple sea urchin) [48-h growth of nauplii] ^i^ | Ni^0^ | SW | >3 (Kanold et al. 2016) |
| *Paramecium caudatum* (ciliophoran) [chemo-attraction, exposure time not reported] | Ni^0^ | FW | 0.0049 (Morgaleva et al. 2015) |
| *Photobacterium phosphoreum* (bacteria) [96-h bioluminescence] | NiO | SW | 335 (Wang et al. 2016) |
| *Pseudokirchneriella subcapitata* (green alga) [72- or 96-h population growth] | Ni^0^ & NiO | FW | 0.35 (Griffitt et al. 2008) [Ni^0^, 96-h] 1.26 (Sousa et al. 2018b) [NiO, 72-h] 6.68 (Nogueira et al. 2015) [NiO, 72-h] 12.5 (Nogueira et al. 2015) [NiO, 72-h] |
| *Pseudomonas aeruginosa* (bacteria) [24-h population growth] | NiO | FW | 28.2 (Kganyago et al. 2018) |
| *Saccharomyces cerevisiae* (yeast) [6- or 24-h cell viability or population growth] | NiO | FW | ~78.8 (Sousa et al. (2019) [24-h] >78.8 (Sousa et al. 2018a) [24-h] 87.4 (Sousa et al. 2018c) [6-h] 92.7 (Sousa et al. 2018a) [6-h] |
| *Salmonella typhi* (bacteria) [24-h population growth] | Ni^0^ & NiO | FW | 33.6 ^b^ (Jeyaraj Pandian et al. 2016) [Ni^0^] 694 (Bhushan et al. 2019) [NiO] |
| *Staphylococcus aureus* (bacteria) [8- or 24-h population growth] | Ni^0^ & NiO | FW | 2.68 (Paul & Neogi 2019) [NiO, 24-h] 5.18 (Paul & Neogi 2019) [NiO, 8-h] 11.6 (Argueta-Figueroa et al. 2014) [Ni^0^, 24-h] >39.4 (Kganyago et al. 2018) [NiO, 24-h] 832 (Bhushan et al. 2019) [NiO, 24-h] |
| *Staphylococcus epidermidis* (bacteria) [24-h population growth] | Ni^0^ | FW | 33.7 ^b^ (Jeyaraj Pandian et al. 2016) |
| *Streptococcus mutans* (bacteria) [24-h population growth] | Ni^0^ | FW | 10.4 (Argueta-Figueroa et al. 2014) |
| *Vibrio fischeri* (bacteria) [30-min bioluminescence] | NiO | SW | <15.8 (Nogueira et al. 2015) |

^a^ FW = fresh-water-based; SW = salt-water-based.

^b^ Log-linear interpolation between the two concentrations that bracketed 50% population growth.

^c^ In the absence of survival data for *C. intestinalis*, morphological development of an early life stage (which is expected to be a more sensitive endpoint than survival of the same organisms) is used as the toxicity endpoint.

^d^ Survival is not used in the meta-analysis as the exposure endpoint for *D. rerio* because the EC50s for embryo hatching generally were approximately 10-fold lower than the LC50s for survival.

^e^ Bioluminescence endpoint is not included here because population growth endpoint was generally more sensitive.

^f^ Photosynthesis is included as the toxicity endpoint for *L. gibba* because (1) no survival data are available for this species and (2) photosynthesis is expected to be a more sensitive endpoint than survival.

^g^ 7-d frond growth of *L. minor* is included here as the toxicity endpoint in the absence of shorter-term growth data.

^h^ In the absence of short-term toxicity data for *O. latipes*, the 7-d early lifestage (ELS) survival LC50 (a “greater-than” value) is used as a conservative underestimate of the shorter-term LC50.

^i^ In the absence of survival data for *P. lividus*, growth of an early life stage (which is expected to be a more sensitive endpoint than survival of the same organisms) is used as the toxicity endpoint.

Table S6. Threshold concentrations (TECs) [represented as 10% effect concentrations (EC10s), geometrics means of the no observed effect concentration (NOEC) and lowest observed effect concentration (LOEC), or geometric means of the minimum inhibitory concentration (MIC) and the next lower exposure concentration (only for bacteria and fungi)] for freshwater and saltwater organisms exposed to nanoparticulate elemental nickel (nano-Ni^0^) or nanoparticulate nickel oxide (nano-NiO) in aqueous-based medium. NR = not reported. Green-highlighted studies received a low quality rating and were not used in the meta-analysis.

| Taxon and endpoint | Nano-metal | Water ^a^ | Individual TECs [either EC10s (E), geometric means of NOEC and LOEC (GMNL), or geometric means of MIC and next lower concentration (GMM)] (mg Ni/L) ^b^ |
| --- | --- | --- | --- |
| *Acartia tonsa* (calanoid copepod) [7-d survival of ELS] | Ni^0^ | SW | 3.77 E (Zhou et al. 2016) 4.48 E (Zhou et al. 2016) |
| *Agrobacterium* sp. PH-08 (bacteria) [6-h population growth] | Ni^0^ | FW | 22.5 E (Le et al. 2014) |
| Anaerobic microbial assemblage (bacteria) [15- to 30-d nitrogen removal] | NiO | FW | >7.88 E (Xu et al. 2019) |
| *Aspergillus clavatus* (fungus) [48-h population growth] | Ni^0^ | FW | <25 E (Jeyaraj Pandian et al. 2016) |
| *Aspergillus fumigatus* (fungus) [48-h population growth] | Ni^0^ | FW | 25.4 ^c^ E (Jeyaraj Pandian et al. 2016) |
| *Aspergillus niger* (fungus) [48-h population growth] | Ni^0^ | FW | <25 E (Jeyaraj Pandian et al. 2016) |
| *Bacillus anthracis* (bacteria) [20- to 24-h population growth] | NiO | FW | 2.28 E (Mishra et al. 2018) |
| *Bacillus subtilis* (bacteria) [6- or 24-h population growth (G) or metabolism of MTT ^d^ (MTT)] | Ni^0^ & NiO | FW | <4.92 GMM (Pang et al. 2009) [NiO, MTT, 24-h] <6.14 GMM (Khalil et al. 2018) [NiO, G, 24-h] 8.69 GMM (Khalil et al. 2018) [NiO, G, 24-h] <20 E (Peng et al. 2018a) [Ni^0^, 6-h, G] <25 E (Jeyaraj Pandian et al. 2016) [Ni^0^, G, 24-h] >39.4 GMM (Pang et al. 2009) [NiO, MTT, 24-h] >39.4 GMM (Pang et al. 2009) [NiO, MTT, 24-h] 406 E (Bhushan et al. 2019) [NiO, G, 24-h] |
| Bacteriophage f2 (virus) [1-h population growth] | Ni^0^ | FW | <40 E (Cheng et al. 2019) |
| *Candida albicans* (fungus) [48-h population growth] | Ni^0^ | FW | 25.4 ^c^ E (Jeyaraj Pandian et al. 2016) |
| *Candida tropicalis* (fungus) [48-h population growth] | Ni^0^ | FW | <25 E (Jeyaraj Pandian et al. 2016) |
| *Chlorella vulgaris* (green alga) [72- or 96-h population growth] | Ni^0^ & NiO | FW & BW | 0.0033 E (Morgaleva et al. 2015) [Ni^0^, NR] 0.00557 E (Oukarroum et al. 2017) [NiO, 96-h] 5.04 E (Li et al. 2017) [NiO, 72-h] 13.2 E (Gong et al. 2011) [NiO, 72-h] |
| *Danio rerio* (zebrafish) [30-d survival of adults] | NiO | FW | 11.9 E (Kovrižnych et al. 2014) |

Table S6 (continued).

| Taxon and endpoint | Nano-metal | Water ^a^ | Individual TECs [either EC10s (E), geometric means of NOEC and LOEC (GMNL), or geometric means of MIC and next lower concentration (GMM)] (mg Ni/L) ^b^ |
| --- | --- | --- | --- |
| *Daphnia magna* (water flea) [21-d reproduction] | NiO | FW | 0.0268 E (Nogueira et al. 2015) 0.106 E (Nogueira et al. 2015) 0.176 E (Gong et al. 2016) |
| *Enterobacter cloacae* (bacteria) [metabolism of MTT ^d^] | NiO | FW | 13.9 GMM (Pang et al. 2009) 27.9 GMM (Pang et al. 2009) >39.4 GMM (Pang et al. 2009) |
| *Enterococcus faecalis* (bacteria) [24-h population growth or metabolism of MTT ^d^] | NiO | FW | 13.9 GMM (Pang et al. 2009) >39.4 E (Kganyago et al. 2018) >39.4 GMM (Pang et al. 2009) >39.4 GMM (Pang et al. 2009) |
| *Escherichia coli* (bacteria) [6-, 8-, or 24-h population growth] ^e^ | Ni^0^ & NiO | FW | 0.222 E (Argueta-Figueroa et al. 2014) [Ni^0^, 24-h] 0.254 E (Paul & Neogi 2019) [NiO, 24-h] 0.512 E (Paul & Neogi 2019) [NiO, 8-h] 0.730 E (Wang et al. 2010) [NiO, 8-h] 1.15 GMM (Kheiri Hafshejani et al. 2018) [Ni^0^, 24-h] 3.60 E (Rajivgandhi et al. 2019) [NiO, 24-h] <3.94 E (Kganyago et al. 2018) [NiO, 24-h] 7.07 GMM (Chaudhary et al. 2015) [Ni^0^, 24-h] 8.69 GMM (Khalil et al. 2018) [NiO, 24-h] <10 E (Zhang et al. 2013) [Ni^0^, 24-h] <20 E (Peng et al. 2018a) [Ni^0^, 6-h] >24.6 GMM (Khalil et al. 2018) [NiO, 24-h] <25 E (Jeyaraj Pandian et al. 2016) [Ni^0^, 24-h] 383 E (Bhushan et al. 2019) [NiO, 24-h] |
| *Klebsiella pneumoniae* (bacteria) [24-h population growth] | Ni^0^ & NiO | FW | 3.94 E (Rajivgandhi et al. 2019) [NiO] >24.6 GMM (Khalil et al. 2018) [NiO] >24.6 GMM (Khalil et al. 2018) [NiO] 25.4 ^d^ E (Jeyaraj Pandian et al. 2016) [Ni^0^] |
| *Klebsiella* sp. (bacteria) [24-h population growth] | Ni^0^ | FW | 7.07 GMM (Chaudhary et al. 2015) |
| *Labeo rohita* (carp) [21-d growth] | Ni^0^ | FW | <25 E (Kanwal et al. 2019) |
| *Lemna minor* (common duckweed) [7-d frond growth] | NiO | FW | <4.10 E (Nogueira et al. 2015) <4.10 E (Nogueira et al. 2015) |
| *Oreochromis mossambicus* (Mozambique tilapia) [14-d survival] | Ni^0^ | FW | >10 E (Jayaseelan et al. 2014) |
| *Oryzias latipes* (medaka) [7-d survival of ELS] | NiO | FW | 39.4 GMNL (Lin et al. 2013) |
| *Photobacterium phosphoreum* (bacteria) [96-h population growth] | NiO | SW | 48.0 E (Wang et al. 2016) |

Table S6 (continued).

| Taxon and endpoint | Nano-metal | Water ^a^ | Individual TECs [either EC10s (E), geometric means of NOEC and LOEC (GMNL), or geometric means of MIC and next lower concentration (GMM)] (mg Ni/L) ^b^ |
| --- | --- | --- | --- |
| *Pseudokirchneriella subcapitata* (green alga) [72-h population growth] | NiO | FW | 0.867 E (Sousa et al. 2018b) <6.46 E (Nogueira et al. 2015) 9.46 E (Nogueira et al. 2015) |
| *Pseudomonas aeruginosa* (bacteria) [24-h population growth (G) or metabolism of MTT ^d^ (MTT)] | Ni^0^ & NiO | FW | <4.92 GMM (Pang et al. 2009) [NiO, MTT] 8.69 GMM (Khalil et al. 2018) [NiO, G] 12.2 GMM (Chaudhary et al. 2015) [Ni^0^, G] 12.4 E (Kganyago et al. 2018) [NiO, G] >24.6 GMM (Khalil et al. 2018) [NiO, G] 27.9 GMM (Pang et al. 2009) [NiO, MTT] >39.4 GMM (Pang et al. 2009) [NiO, MTT] |
| *Saccharomyces cerevisiae* (yeast) [6- or 24-h cell viability or population growth] | NiO | FW | 37.2 E (Sousa et al. 2018c) [6-h] 38.8 E (Sousa et al. 2018a) [6-h] <78.8 E (Sousa et al. 2019) [24-h] >78.8 E (Sousa et al. 2018a) [24-h] |
| *Salmonella typhi* (bacteria) [24-h population growth] | Ni^0^ & NiO | FW | <25 E (Jeyaraj Pandian et al. 2016) [Ni^0^) 271 E (Bhushan et al. 2019) [NiO] |
| *Staphylococcus aureus* (bacteria) [8- or 24-h population growth (G) or metabolism of MTT ^d^ (MTT)] | Ni^0^ & NiO | FW | 0.430 E (Argueta-Figueroa et al. 2014) [Ni^0^, G, 24-h] <0.81 GMM (Kheiri Hafshejani et al. 2018) [Ni^0^, G, 24-h] 0.874 E (Paul & Neogi 2019) [NiO, G, 8-h] 1.21 E (Paul & Neogi 2019) [NiO, G, 24-h] <4.92 GMM (Pang et al. 2009) [NiO, MTT, 24-h] 8.69 GMM (Khalil et al. 2018) [NiO, G, 24-h] 12.2 GMM (Chaudhary et al. 2015) [Ni^0^, G, 24-h] >24.6 GMM (Khalil et al. 2018) [NiO, G, 24-h] 27.9 GMM (Pang et al. 2009) [NiO, MTT, 24-h] ~39.4 E (Kganyago et al. 2018) [NiO, G, 24-h] >39.4 GMM (Pang et al. 2009) [NiO, MTT, 24-h] 404 E (Bhushan et al. 2019) [NiO, G, 24-h] |
| *Staphylococcus epidermidis* (bacteria) [24-h population growth] | Ni^0^ & NiO | FW | <6.14 GMM (Khalil et al. 2018) [NiO] >24.6 GMM (Khalil et al. 2018) [NiO] <25 E (Jeyaraj Pandian et al. 2016) [Ni^0^] |
| *Streptococcus mutans* (bacteria) [24-h population growth] | Ni^0^ | FW | 0.476 E (Argueta-Figueroa et al. 2014) |
| *Vibrio fischeri* (bacteria) [30-min bioluminescence] | NiO | SW | <15.8 E (Nogueira et al. 2015) |

^a^ BW = brackish-water-based; FW = fresh-water-based; SW = salt-water-based.

^b^ For each toxicity test or experiment, the lowest value of the three endpoints [L(E)C10, geometric mean of NOEC and LOEC, or geometric mean of MIC and the next lower exposure concentration] is reported.

^c^ Log-linear interpolation between the two concentrations that bracketed 10% population growth.

^d^ MTT = 3-(4,5-dimethylthiazol-2-yl)-2,5-diphenyltetrazolium bromide.

^e^ Bioluminescence endpoint for genetically-modified *E. coli* is not included here because population growth endpoint was generally more sensitive.

Table S7. Toxicity of Ni-containing complex engineered nanomaterials (CENs) to aquatic organisms. A = agar; L(E)C10 = 10% lethal (or effect) concentration; L(E)C50 = median lethal (or effect) concentration; FW = freshwater-based; L = low; M= medium; MIC = minimum inhibitory concentration; MM = microbial growth medium; NA = not applicable because the test was not conducted in aqueous medium; nano-Ni = Ni-containing nanomaterial; NC = could not be calculated; NP = nanoparticle; NR = not reported; SW = salt water; SWW = synthetic wastewater.

| Form of nano-Ni CEN | Taxon | Medium | Exposure time (d) | Toxicity endpoint | MIC (mg Ni/L) ^a^ | L(E)C10 (mg Ni/L) ^a^ | L(E)C50 (mg Ni/L) ^a^ | Quality rating | Reference |
| --- | --- | --- | --- | --- | --- | --- | --- | --- | --- |
| Ni^0^-Cu^0^ | *Escherichia coli* (bacteria) | FW MM | 1.0 | Population growth |  | 0.149 ^b^ | 7.03 ^b^ | M | Argueta-Figueroa et al. (2014) |
|  | *Staphylococus aureus* (bacteria) | FW MM | 1.0 | Population growth |  | 0.408 ^b^ | 12.2 ^b^ | M |  |
|  | *Streptococcus mutans* (bacteria) | FW MM | 1.0 | Population growth |  | 0.600 ^b^ | 14.0 ^b^ | M |  |
| Ni^0^, capped with exopolysaccharide | *Pseudomonas aeruginosa* (bacteria) | FW MM | 0.83 | Population growth |  | 370 | 695 | L | Garza-Cervantes et al. (2019) |
| Ni^0^, capped with exopolysaccharide | *Staphylococcus aureus* (bacteria) | FW MM | 0.83 | Population growth |  | 650 | 1,120 | L | Garza-Cervantes et al. (2019) |
| NiO-CuO | *Escherichia coli* (bacteria) | FW MM | 0.33 | Population growth |  | <0.309 ^c^ | 0.735 ^c^ | M | Paul & Neogi (2019) |
|  |  | FW MM | 1.0 | Population growth | 0.927 ^d^ | <0.309 ^e^ | <0.309 ^e^ | M |  |
|  | *Staphylococus aureus* (bacteria) | FW MM | 0.33 | Population growth |  | <0.309 ^c^ | 0.603 ^c^ | M |  |
|  |  | FW MM | 1.0 | Population growth | 1.51 ^d^ | <0.309 ^e^ | <0.309 ^e^ | M |  |
| NiAl_0.5_Fe_1.5_O_4_ | *Escherichia coli*  (bacteria) | FW A | 1.0 | Zone of inhibition | NA | NA | NA | L | Naik et al. (2018) |
|  | *Pseudomonas aeruginosa* (bacteria) | FW A | 1.0 | Zone of inhibition | NA | NA | NA | L |  |
|  | *Salmonella typhi* (bacteria) | FW A | 1.0 | Zone of inhibition | NA | NA | NA | L |  |

Table S7 (continued).

| Form of nano-Ni CEN | Taxon | Medium | Exposure time (d) | Toxicity endpoint | MIC (mg Ni/L) ^a^ | L(E)C10 (mg Ni/L) ^a^ | L(E)C50 (mg Ni/L) ^a^ | Quality rating | Reference |
| --- | --- | --- | --- | --- | --- | --- | --- | --- | --- |
| Ni-Fe (1% Ni by wt) | *Escherichia coli*  (bacteria) | FW MM | 0.042 | Population growth |  | <0.300 | <0.300 | L | Dong et al. (2019) |
| Ni-Fe (3% Ni by wt) | *Escherichia coli*  (bacteria) | FW MM | 0.042 | Population growth |  | <0.900 | <0.900 | L | Dong et al. (2019) |
| Ni-Fe (5% Ni by wt) | *Escherichia coli*  (bacteria) | FW MM | 0.042 | Population growth |  | <1.50 | <1.50 | L | Dong et al. (2019) |
| Ni-Fe, starch-stabilized (5% Ni & 0.4% starch by wt) | *Escherichia coli*  (bacteria) | FW MM | 0.042 | Population growth |  | <1.49 | <1.49 | L | Dong et al. (2019) |
| Ni-Fe, starch-stabilized (5% Ni & 2% starch by wt) | *Escherichia coli*  (bacteria) | FW MM | 0.042 | Population growth |  | <1.47 | <1.47 | L | Dong et al. (2019) |
| Ni-Fe, starch-stabilized (5% Ni & 4% starch by wt) | *Escherichia coli*  (bacteria) | FW MM | 0.042 | Population growth |  | <1.44 | <1.44 | L | Dong et al. (2019) |
| Ni-Fe_3_ | Bacteriophage f2 | FW MM | 0.042 | Population growth |  | <10.4 | <10.4 | L | Cheng et al. (2019) |
| Ni-Fe_5_ | Bacteriophage f2 | FW MM | 0.042 | Population growth |  | <6.95 | <6.95 | L | Cheng et al. (2019) |
| Ni-Fe_10_ | Bacteriophage f2 | FW MM | 0.042 | Population growth |  | <3.80 | <3.80 | L | Cheng et al. (2019) |
| (NiO)_0.025_(Fe_2_O_3_)_0.1_ | *Bacillus subtilis* (bacteria) | FW MM | 1.0 | Population growth |  | 36.2 | 81.7 | L | Bhushan et al. (2019) |
|  | *Escherichia coli* (bacteria) | FW MM | 1.0 | Population growth |  | 42.2 | 83.6 | L |  |
|  | *Salmonella typhi* (bacteria) | FW MM | 1.0 | Population growth |  | 39.0 | 72.4 | L |  |
|  | *Staphylococus aureus* (bacteria) | FW MM | 1.0 | Population growth |  | 42.5 | 86.3 | L |  |

Table S7 (continued).

| Form of nano-Ni CEN | Taxon | Medium | Exposure time (d) | Toxicity endpoint | MIC (mg Ni/L) ^a^ | L(E)C10 (mg Ni/L) ^a^ | L(E)C50 (mg Ni/L) ^a^ | Quality rating | Reference |
| --- | --- | --- | --- | --- | --- | --- | --- | --- | --- |
| NiFe_2_O_4_ | *Bacillus cereus* (bacteria) | FW A | NR | Zone of inhibition | NA | NA | NA | L | Mohebbi et al. (2019) |
|  | *Bacillus subtilis* (bacteria) | FW MM | 1.0 | Population growth |  | 78.1 | 146 | L | Bhushan et al. (2019) |
|  |  | FW MM | 3.0 | Population growth |  |  | NC ^f^ | L | Udhaya et al. (2019) |
|  |  | FW A | NR | Cell viability | NA | NA | NA | L |  |
|  | *Enterococcus faecalis* (bacteria) | FW A | NR | Cell viability | NA | NA | NA | L |  |
|  | *Escherichia coli* (bacteria) | FW MM | 1.0 | Population growth |  | 92.4 | 164 | L | Bhushan et al. (2019) |
|  |  | FW A | NR | Zone of inhibition | NA | NA | NA | L | Mohebbi et al. (2019) |
|  |  | FW A | 1.0 | Zone of inhibition | NA | NA | NA | L | Naik et al. (2018) |
|  |  | FW A | NR | Cell viability | NA | NA | NA | L | Udhaya et al. (2019) |
|  | *Pseudomonas aeruginosa* (bacteria) | FW A | 1.0 | Zone of inhibition | NA | NA | NA | L | Naik et al. (2018) |
|  |  | FW MM | 3.0 | Population growth |  |  | NC ^f^ | L | Udhaya et al. (2019) |
|  |  | FW A | NR | Cell viability | NA | NA | NA | L |  |
|  | *Salmonella typhi* (bacteria) | FW MM | 1.0 | Population growth |  | 92.0 | 138 | L | Bhushan et al. (2019) |
|  |  | FW A | 1.0 | Zone of inhibition | NA | NA | NA | L | Naik et al. (2018) |
|  | *Salmonella typhimurium* (bacteria) | FW A | NR | Zone of inhibition | NA | NA | NA | L | Mohebbi et al. (2019) |
|  | *Staphylococus aureus* (bacteria) | FW MM | 1.0 | Population growth |  | 100 | 172 | L | Bhushan et al. (2019) |
|  |  | FW A | NR | Zone of inhibition | NA | NA | NA | L | Mohebbi et al. (2019) |
|  | Anaerobic microbial assemblage | FW SWW | 7 | Methane production |  | >100 ^g^ | >100 ^g^ | L | Chen et al. (2018) |

Table S7 (continued).

| Form of nano-Ni CEN | Taxon | Medium | Exposure time (d) | Toxicity endpoint | MIC (mg Ni/L) ^a^ | L(E)C10 (mg Ni/L) ^a^ | L(E)C50 (mg Ni/L) ^a^ | Quality rating | Reference |
| --- | --- | --- | --- | --- | --- | --- | --- | --- | --- |
| NiFe_2_O_4_ @ABS (acrylonitrile butadiene styrene) | *Bacillus cereus* (bacteria) | FW A | NR | Zone of inhibition | NA | NA | NA | L | Mohebbi et al. (2019) |
|  | *Escherichia coli* (bacteria) | FW A | NR | Zone of inhibition | NA | NA | NA | L |  |
|  | *Salmonella typhimurium* (bacteria) | FW A | NR | Zone of inhibition | NA | NA | NA | L |  |
|  | *Staphylococus aureus* (bacteria) | FW A | NR | Zone of inhibition | NA | NA | NA | L |  |
| NiFe_2_O_4_ @ABS (acrylonitrile butadiene styrene)@Ag | *Bacillus cereus* (bacteria) | FW A | NR | Zone of inhibition | NA | NA | NA | L | Mohebbi et al. (2019) |
|  | *Escherichia coli* (bacteria) | FW A | NR | Zone of inhibition | NA | NA | NA | L |  |
|  | *Salmonella typhimurium* (bacteria) | FW A | NR | Zone of inhibition | NA | NA | NA | L |  |
|  | *Staphylococus aureus* (bacteria) | FW A | NR | Zone of inhibition | NA | NA | NA | L |  |
| NiFe_2_O_4_ @Ag | *Bacillus cereus* (bacteria) | FW A | NR | Zone of inhibition | NA | NA | NA | L | Mohebbi et al. (2019) |
|  | *Escherichia coli* (bacteria) | FW A | NR | Zone of inhibition | NA | NA | NA | L |  |
|  | *Salmonella typhimurium* (bacteria) | FW A | NR | Zone of inhibition | NA | NA | NA | L |  |
|  | *Staphylococus aureus* (bacteria) | FW A | NR | Zone of inhibition | NA | NA | NA | L |  |
| NiFe_2_O_4_, Ag-coated | *Alternaria solani* (fungus) | FW A | 6 | Population growth | NA | NA | NA | L | Golkhatmi et al. (2017) |
|  | *Bacillus subtilis* (bacteria) | FW A | 2.0 | Cell viability | NA | NA | NA | L |  |

Table S7 (continued).

| Form of nano-Ni CEN | Taxon | Medium | Exposure time (d) | Toxicity endpoint | MIC (mg Ni/L) ^a^ | L(E)C10 (mg Ni/L) ^a^ | L(E)C50 (mg Ni/L) ^a^ | Quality rating | Reference |
| --- | --- | --- | --- | --- | --- | --- | --- | --- | --- |
| NiFe_2_O_4_, Ag-coated (cont.) | *Fusarium oxysporum* (fungus) | FW A | 6 | Population growth | NA | NA | NA | L | Golkhatmi et al. (2017) |
|  | *Pseudomonas syringae* (bacteria) | FW A | 2.0 | Cell viability | NA | NA | NA | L |  |
| NiFe2O4 @PRh (polyrhodanine) | *Enterococcus faecalis* (bacteria) | FW MM | 1 | Cell viability | 4,880 |  |  | L | Lashkenari et al. (2019) |
|  | *Staphylococus aureus* (bacteria) | FW MM | 1 | Cell viability | 2,440 |  |  | L |  |
| NiFe_2_O_3_Zn | *Hydra attenuata* (invertebrate) | FW | 4.0 | Morphology |  |  | 0.14 – 1.4 ^h^ | L | Blaise et al. (2008) |
|  | *Pseudokirchneriella subcapitata* (green alga) | FW | 3.0 | Population growth |  | 0.014 – 0.14 ^h^ |  | L |  |
|  | *Thamnocephalus platyurus* (invertebrate) | FW | 1.0 | Survival |  |  | 1.4-14 ^h^ | L |  |
|  | *Vibrio fischeri* (bacteria) | SW | 0.01 | Bioluminescence |  | 1.4 – 14 ^h^ |  | L |  |
|  | MARA ^i^ | FW MM | 1.0 | Population growth |  |  | 1.4 – 14 ^h^ | L |  |
| NiFe_4_O_4_Zn | Anaerobic microbial assemblage | FW SWW | 7 | Methane production |  | 1.19 ^g^ | 5.46 ^g^ | L; | Chen et al. (2018) |
| α-NiMoO_4_ | *Pseudomonas aeruginosa* (bacteria) | FW MM | 0.12 | Cell viability |  | <5,370 ^j^ | <5,370 ^j^ | M | Ray et al. (2018) |
| α-NiMoO_4_, Co-doped | *Pseudomonas aeruginosa* (bacteria) | FW MM | 0.12 | Cell viability |  |  | <20,000 ^k^ | M | Ray et al. (2018) |
| α-NiMoO_4_, Cu-doped | *Pseudomonas aeruginosa* (bacteria) | FW MM | 0.12 | Cell viability |  |  | <20,000 ^k^ | M | Ray et al. (2018) |

Table S7 (continued).

| Form of nano-Ni CEN | Taxon | Medium | Exposure time (d) | Toxicity endpoint | MIC (mg Ni/L) ^a^ | L(E)C10 (mg Ni/L) ^a^ | L(E)C50 (mg Ni/L) ^a^ | Quality rating | Reference |
| --- | --- | --- | --- | --- | --- | --- | --- | --- | --- |
| Ni_0.14_Co_0.14_Li_0.52_Mn_0.72_O_2_ | *Shewanella oneidensis* (bacteria) | FW MM | 3.0 | Respiration rate |  |  | >0.448 ^l^ | L | Gunsolus et al. (2017) |
| Ni_0.23_Co_0.22_Li_0.61_Mn_0.55_O_2_ | *Shewanella oneidensis* (bacteria) | FW MM | 3.0 | Respiration rate |  |  | >0.726 ^m^ | L | Gunsolus et al. (2017) |
| Ni_0.31_Co_0.30_Li_0.68_Mn0_.39_O_2_ | *Shewanella oneidensis* (bacteria) | FW MM | 3.0 | Respiration rate |  |  | <0.968 ^n^ | L | Gunsolus et al. (2017) |
| Ni_0.32_Co_0.35_Li_0.75_Mn_0.33_O_2_ nanosheets | *Shewanella oneidensis* (bacteria) | FW MM | 3.0 | Respiration rate |  |  | <0.991 ^o^ | L | Hang et al. (2018) |
|  |  | FW MM | NR | Population growth |  | 0.124 ^p, q^ | 3.6 ^p, q^ | L |  |
| Ni_0.33_Co_0.33_Li_0.27_Mn_0.33_O_2_ | *Daphnia magna* (water flea) neonate | FW | 2.0 | Survival |  | >5.33 ^r^ | >5.33 ^r^ | M | Bozich et al. (2017) |
|  | *Daphnia magna* (water flea) adult | FW | 21 | Survival Reproduction |  | 0.122 ^s^ 0.0749 ^t^ | 0.194 ^s^ 0.118 ^t^ | M |  |
| Ni_0.33_Co_0.33_Li_0.33_Mn_0.33_O_2_ | *Shewanella oneidensis* (bacteria) | FW MM | 2.5 | Respiration rate |  |  | >1.06 ^u^ | L | Hang et al. (2016) |
|  |  | FW MM | 2.5 | Population growth |  | < 1.06 ^v, w^ | < 1.06 ^v, w^ | L |  |
| Ni_0.33_Co_0.33_Li_0.45_Mn_0.34_O_2_ | *Chironomus riparius* (insect) larva | FW + sand | 7 | Somatic growth |  |  | NA ^x^ | L | Niemuth et al. (2019) |
|  | *Chironomus riparius* (insect) adult | FW + sand | 7 | Time to emergence |  |  | NA ^x^ | L |  |
| Ni_0.33_Co_0.33_Li_0.92_Mn_0.34_O_2_ nanoblocks | *Shewanella oneidensis* (bacteria) | FW MM | 3.0 | Respiration rate |  |  | >10.1 ^y^ | L | Hang et al. (2018) |
|  |  | FW MM | NR | Population growth |  | 0.467 ^p, z^ | 18.8 ^p, z^ | L |  |
| Ni_0.33_Co_0.33_LiMn0_.33_O_2_ battery-grade powder | *Shewanella oneidensis* (bacteria) | FW MM | 3.0 | Respiration rate |  |  | >10.9 ^aa^ | L | Hang et al. (2018) |
|  |  | FW MM | NR | Population growth |  | 33.1 ^p, ab^ | 358 ^p, ab^ | L |  |

Table S7 (continued).

| Form of nano-Ni CEN | Taxon | Medium | Exposure time (d) | Toxicity endpoint | MIC (mg Ni/L) ^a^ | L(E)C10 (mg Ni/L) ^a^ | L(E)C50 (mg Ni/L) ^a^ | Quality rating | Reference |
| --- | --- | --- | --- | --- | --- | --- | --- | --- | --- |
| Ni-doped γ-Al_2_O_3_ | *Rhinella arenarum* (Argentine common toad) larva | FW | 4.0 7.0 14.0 21 | Survival Survival Survival Survival |  |  | <1.55 ^ac^ <1.55 ^ac^ <1.30 ^ac^ <0.83 ^ac^ | M | Svartz et al. (2017) |
|  |  | FW | 4.0 7.0 14.0 | Survival Survival Survival |  |  | <110 <80.0 <5.11 | M | Svartz et al. (2019) |
|  | *Vibrio fischeri* (bacteria) | SW | 0.01 | Bioluminescence |  |  | NC ^ad^ | L | Svartz et al. (2017) |
| NiO-doped γ-Al_2_O_3_ | *Rhinella arenarum* (Argentine common toad) larva | FW | 4.0 7.0 14.0 | Survival Survival Survival |  |  | <60.7 <30.0 <4.03 | M | Svartz et al. (2019) |
| Ni embedded in SiO_2_ | *Danio rerio* (zebrafish) embryo | FW | 4 | Survival |  |  | >400 ^ae^ | M | Mahoney et al. (2016) |
|  |  | FW | 4 | Malformations |  |  | >200 ^af^ | M |  |
| NiO annealed onto SiO_2_ | *Staphylococus aureus* (bacteria) | FW A | 0.12 | Cell viability | NA | NA | NA | L | Hou et al. (2018b) |
| Ni-doped TiO_2_ | *Carassius auratus* (goldfish) | FW | 4.0 | Survival |  |  | 77.5 ^ag^ | M | Pirsaheb et al. (2019) |
|  | *Cyprinus carpio* (common carp) | FW | 4.0 | Survival |  |  | 95.6 ^ag^ | M |  |
|  | *Escherichia coli* (bacteria) | FW MM | 0.21 | Cell viability |  |  | NC ^ah^ | L | Ananpattarachai et al. (2016) |
|  | *Staphylococus aureus* (bacteria) | FW MM | 0.21 | Cell viability |  |  | NC ^ai^ | L |  |
| NiO supported on natural clinoptilite zeolite | *Escherichia coli* (bacteria) | FW | 1.0 | Population growth |  |  | >5.20 ^aj^ | L | Hrenovic et al. (2012) |
|  | *Staphylococus aureus* (bacteria) | FW | 1.0 | Population growth |  |  | >5.20 ^ak^ | L |  |

Table S7 (continued).

| Form of nano-Ni CEN | Taxon | Medium | Exposure time (d) | Toxicity endpoint | MIC (mg Ni/L) ^a^ | L(E)C10 (mg Ni/L) ^a^ | L(E)C50 (mg Ni/L) ^a^ | Quality rating | Reference |
| --- | --- | --- | --- | --- | --- | --- | --- | --- | --- |
| NiO/graphene | *Escherichia coli* (bacteria) | FW MM | 1.0 | Population growth |  | 1.30 | 5.99 | L | Rajivgandhi et al. (2019) |
|  | *Klebsiella pneumoniae* (bacteria) | FW MM | 1.0 | Population growth |  | 1.66 | 7.72 | L |  |
| Ni-coated Pd nanocoils | *Escherichia coli* (bacteria) | FW MM | 0.04 | Population growth |  | >5.46 ^al, am^ | >5.46 ^al, am^ | L | Hoop et al. (2016) |
| Ni- & Ag-coated Pd nanowires | *Escherichia coli* (bacteria) | FW MM | NR | Population growth |  | <0.410 ^an, ao^ | 0.631 ^an, ao^ | L | Hoop et al. (2016) |
| Ni- & Ag-coated  Pd nanocoils | *Escherichia coli* (bacteria) | FW MM | 0.04 | Population growth |  | <4.10 ^al, ap^ | <4.10 ^al, ap^ | L | Hoop et al. (2016) |
|  |  | FW MM | 0.25 | Population growth |  | <0.410 ^ap, aq^ | <0.410 ^ap, aq^ | L |  |
|  |  | FW MM | NR | Cell viability |  | <0.410 ^an, ap^ | 0.535 ^an, ap^ | L |  |
|  |  | FW MM | NR | Cell viability (anaerobic) |  | <0.410 ^ap, ar^ | 5.83 ^ap, ar^ | L |  |
|  | *Staphylococus aureus* (bacteria) | FW MM | 0.08 | Cell viability |  | <8.21 ^ap, as^ | <8.21 ^ap, as^ | L |  |
| Ni-ZnO/rGO/Nylon6 ^at^ | *Bacillus subtilis* (bacteria) | FW MM | 0.17 | Population growth |  |  | NC ^au^ | L | Sedghi et al. (2017) |
|  | *Escherichia coli* (bacteria) | FW MM | 0.17 | Population growth |  |  | NC ^au^ | L |  |
| Ni-ZnO/rGO/Nylon6/Ag ^av^ | *Bacillus subtilis* (bacteria) | FW MM | 0.17 | Population growth |  |  | NC ^au^ | L | Sedghi et al. (2017) |
|  | *Escherichia coli* (bacteria) | FW MM | 0.17 | Population growth |  |  | NC ^au^ | L |  |

Table S7 (continued).

| Form of nano-Ni CEN | Taxon | Medium | Exposure time (d) | Toxicity endpoint | MIC (mg Ni/L) ^a^ | L(E)C10 (mg Ni/L) ^a^ | L(E)C50 (mg Ni/L) ^a^ | Quality rating | Reference |
| --- | --- | --- | --- | --- | --- | --- | --- | --- | --- |
| NiO infused into electrospun poly-ε-caprolactone/gelatin hybrid nanofibrous mat | *Escherichia coli* (bacteria) | FW MM | 1.0 | Cell viability |  | NC | NC | L | Ramalingam et al. (2019) |
|  | *Pseudomonas aeruginosa* (bacteria) | FW MM | 1.0 | Cell viability |  | NC | NC | L |  |
|  | *Staphylococus aureus* (bacteria) | FW MM | 1.0 | Cell viability |  | NC | NC | L |  |
| NiO & PHB [poly(3-hydroxybutyrate)] nanocomposite | *Escherichia coli* (bacteria) | FW A | 1.0 | Zone of inhibition | NA | NA | NA | L | Abdelwahab et al. (2018) |
|  | *Klebsiella pneumoniae* (bacteria) | FW A | 1.0 | Zone of inhibition | NA | NA | NA | L |  |
|  | *Staphylococus aureus* (bacteria) | FW A | 1.0 | Zone of inhibition | NA | NA | NA | L |  |
| NiO (3%) & PHB [poly(3-hydroxy-butyrate)]/PEG (polyethylene glycol) nanocomposite | *Escherichia coli* (bacteria) | FW A | 1.0 | Zone of inhibition | NA | NA | NA | L |  |
|  | *Klebsiella pneumoniae* (bacteria) | FW A | 1.0 | Zone of inhibition | NA | NA | NA | L |  |
|  | *Staphylococus aureus* (bacteria) | FW A | 1.0 | Zone of inhibition | NA | NA | NA | L |  |
| NiO (5%) & PHB [poly(3-hydroxy-butyrate)]/PEG (polyethylene glycol) nanocomposite | *Escherichia coli* (bacteria) | FW A | 1.0 | Zone of inhibition | NA | NA | NA | L |  |
|  | *Klebsiella pneumoniae* (bacteria) | FW A | 1.0 | Zone of inhibition | NA | NA | NA | L |  |
|  | *Staphylococus aureus* (bacteria) | FW A | 1.0 | Zone of inhibition | NA | NA | NA | L |  |

Table S7 (continued).

| Form of nano-Ni CEN | Taxon | Medium | Exposure time (d) | Toxicity endpoint | MIC (mg Ni/L) ^a^ | L(E)C10 (mg Ni/L) ^a^ | L(E)C50 (mg Ni/L) ^a^ | Quality rating | Reference |
| --- | --- | --- | --- | --- | --- | --- | --- | --- | --- |
| NiO & PHB [poly(3-hydroxybutyrate)] nanocomposite with adsorbed norfloxacin (an antibiotic) | *Escherichia coli* (bacteria) | FW A | 1.0 | Zone of inhibition | NA | NA | NA | L | Abdelwahab et al. (2018) |
|  | *Klebsiella pneumoniae* (bacteria) | FW A | 1.0 | Zone of inhibition | NA | NA | NA | L |  |
|  | *Staphylococus aureus* (bacteria) | FW A | 1.0 | Zone of inhibition | NA | NA | NA | L |  |
| NiO (3%) & PHB [poly(3-hydroxy-butyrate)]/PEG (polyethylene glycol) nanocomposite with adsorbed norfloxacin (an antibiotic) | *Escherichia coli* (bacteria) | FW A | 1.0 | Zone of inhibition | NA | NA | NA | L |  |
|  | *Klebsiella pneumoniae* (bacteria) | FW A | 1.0 | Zone of inhibition | NA | NA | NA | L |  |
|  | *Staphylococus aureus* (bacteria) | FW A | 1.0 | Zone of inhibition | NA | NA | NA | L |  |
| NiO (5%) & PHB [poly(3-hydroxy-butyrate)]/PEG (polyethylene glycol) nanocomposite with adsorbed norfloxacin (an antibiotic) | *Escherichia coli* (bacteria) | FW A | 1.0 | Zone of inhibition | NA | NA | NA | L |  |
|  | *Klebsiella pneumoniae* (bacteria) | FW A | 1.0 | Zone of inhibition | NA | NA | NA | L |  |
|  | *Staphylococus aureus* (bacteria) | FW A | 1.0 | Zone of inhibition | NA | NA | NA | L |  |

Table S7 (continued).

^a^ Blank entries indicate no EC50, MIC, or MBC was reported and a value could not be calculated from the reported results.

^b^ Calculated from results in Figure 4 in Argueta-Figueroa et al. (2014), using their reported 46.76 wt % Ni in the Cu-Ni nanoparticles.

^c^ Calculated from results in Table 4 in Paul and Neogi (2019), using their reported 30.90 wt % Ni in the CuO-NiO nanoparticles.

^d^ Calculated from results in Table 3 in Paul and Neogi (2019), using their reported 30.90 wt % Ni in the CuO-NiO nanoparticles.

^e^ Calculated from results in Figure 10c in Paul and Neogi (2019), using their reported 30.90 wt % Ni in the CuO-NiO nanoparticles.

^f^ Could not be calculated because Udhaya et al. (2019) did not report the concentrations of NPs to which the bacteria were exposed.

^g^ Inferred from results in Figure 1in Chen et al. (2018).

^h^ Calculated from results in Table II in Blaise et al. (2008), assuming 14.26 wt % Ni in NiFe_2_O_3_Zn.

^i^ MARA = Microbial Array for Risk Assessment (11 species listed in Table 1 in Gabrielson et al. 2003).

^j^ Inferred from results in Figure 9a in Ray et al. (2018), assuming 26.84 wt % Ni in α-NiMoO_4_.

^k^ Could not be calculated because Ray et al. (2018) did not report the percentage of Ni in the Co-doped α-NiMoO_4_ to which the bacteria were exposed. However, an EC50 of <20,000 mg NP/L can be inferred from results in their Figure 9a.

^l^ Inferred from results in Figures 7c and 8c in Gunsolus et al. (2017), assuming 8.97 wt % Ni in Ni_0.14_Co_0.14_Li_0.52_Mn_0.72_O_2_.

^m^ Inferred from results in Figures 7b and 8b in Gunsolus et al. (2017), assuming 14.53 wt % Ni in Ni_0.23_Co_0.22_Li_0.61_Mn_0.55_O_2_.

^n^ Inferred from results in Figures 7a and 8a in Gunsolus et al. (2017), assuming 19.35 wt % Ni in Ni_0.31_Co_0.30_Li_0.68_Mn_0.39_O_2_.

^o^ Inferred from results in Figure 4 in Hang et al. (2018), assuming 19.82 wt % Ni in Ni_0.32_Co_0.35_Li_0.75_Mn_0.33_O_2_.

^p^ Calculated from results in Figure 5a in Hang et al. (2018).

^q^ Ni concentration based on calculated mass percentage of Ni in the nanomaterial (19.82%), using atomic percentages reported by Hang et al. (2018).

^r^ Inferred from result reported in text in Bozich et al. (2017), assuming 21.33 wt % Ni in Ni_0.33_Co_0.33_Li_0.27_Mn_0.33_O_2_.

^s^ Calculated from results in Figure 2B in Bozich et al. (2017), assuming 21.33 wt % Ni in Ni_0.33_Co_0.33_Li_0.27_Mn_0.33_O_2_.

^t^ Calculated from results in Figure 3A in Bozich et al. (2017), assuming 21.33 wt % Ni in Ni_0.33_Co_0.33_Li_0.27_Mn_0.33_O_2_.

^u^ Calculated from results in Figure 2a in Hang et al. (2016), assuming 21.23 wt % Ni in Ni_0.33_Co_0.33_Li_0.33_Mn_0.33_O_2_.

^v^ Inferred from results in Figure 2b in Hang et al. (2016).

^w^ Ni concentration based on calculated mass percentage of Ni in the nanomaterial (21.23%), using atomic percentages reported by Hang et al. (2016).

^x^ Midge larvae were in the sand, and nano-Ni particles settled onto the sand; thus, the aqueous exposure concentration changed as the particles settled during the test, and it does not represent the concentration of particles ingested by the larvae.

^y^ Inferred from results in Figure 4 in Hang et al. (2018), assuming 20.20 wt % Ni in Ni_0.33_Co_0.33_Li_0.92_Mn_0.34_O_2_.

^z^ Ni concentration based on calculated mass percentage of Ni in the nanomaterial (20.20%), using atomic percentages reported by Hang et al. (2018).

^aa^ Inferred from results in Figure 4 in Hang et al. (2018), assuming 21.78 wt % Ni in Ni_0.33_Co_0.33_Mn_0.33_O_2_.

^ab^ Ni concentration based on calculated mass percentage of Ni in the nanomaterial (21.78%), using atomic percentages reported by Hang et al. (2018).

^ac^ 96- and 504-h LC50s were 1.55 and 0.83 mg NP/L, respectively, and 504-h NOEC was 0.05 mg NP/L [from text and Figure 3 in Svartz et al. (2017)]; but Ni content of Ni-doped alumina NPs was not analyzed, thus not allowing calculation of the LC50s in units of mg Ni/L.

^ad^ EC50 reported in Table 3 in Svartz et al. (2017) was 16.1 %v/v; but Ni content of Ni-doped alumina NPs was not analyzed, thus not allowing calculation of the EC50 in units of mg Ni/L.

^ae^ Calculated from results in Figure 5 in Mahoney et al. (2016).

^af^ Calculated from results in Figure 6 in Mahoney et al. (2016).

Table S7 (continued).

^ag^ L(E)C50 expressed as mg of total nanoparticle/L instead of as mg Ni/L, because Pirsaheb et al. (2019) did not report the mass percent of Ni in the nanoparticles.

^ah^ Could not be calculated because Ananpattarachai et al. (2016) did not report the percentage of Ni in the Ni-doped TiO_2_ to which the bacteria were exposed. However, an EC50 of >10^6^ mg NP/L can be inferred from results in their Figure 9.

^ai^ Could not be calculated because Ananpattarachai et al. (2016) did not report the percentage of Ni in the Ni-doped TiO_2_ to which the bacteria were exposed. However, an EC50 of >10^6^ mg NP/L can be inferred from results in their Figure 8.

^aj^ Inferred from results in Figure 1a and Table 1 in Hrenovic et al. (2012), using the 0.52% Ni in the nanomaterial reported by the authors.

^ak^ Inferred from results in Figure 1b in Hrenovic et al. (2012), using the 0.52% Ni in the nanomaterial reported by the authors.

^al^ Inferred from results in text and Figure 2a in Hoop et al. (2016).

^am^ Ni concentration based on calculated mass percentage of Ni in the nanocoils (10.92%), assuming the ratio of the atomic percentages for Ni and Pd reported by Hoop et al. (2016) for Ag- & Ni-coated Pd nanocoils was the same as the ratio for Ni-coated Pd nanocoils.

^an^ Inferred from results in Figure S3 in Hoop et al. (2016).

^ao^ Ni concentration calculated assuming nanowires had same mass percentage of Ni as the nanocoils (8.21%).

^ap^ Ni concentration based on calculated mass percentage of Ni in the nanocoils (8.21%), using atomic percentages reported by Hoop et al. (2016).

^aq^ Inferred from results in text and Figure 2b in Hoop et al. (2016).

^ar^ Inferred from results in Figure S5 in Hoop et al. (2016).

^as^ Inferred from results in text in Hoop et al. (2016).

^at^ Ni-ZnO-containing graphene oxide electrospun into nylon 6 nanofibers.

^au^ Could not be calculated because Sedghi et al. (2017) did not report the concentrations of NPs to which the bacteria were exposed.

^av^ Ni-ZnO-containing graphene oxide electrospun into nylon 6 nanofibers and decorated with Ag NPs.

Table S8. Studies not used in the meta-analysis of the toxicity of nanoparticulate nickel to aquatic organisms, because they did not pass the quality screening.

| **Authors (year)** | **Publication** | **Form of nano-Ni** | **Taxa** | **Reason(s) for not including study in meta-analysis** |
| --- | --- | --- | --- | --- |
| Abdelwahab et al. (2018) | Int. J. Biol. Macromol. 114:717-727. | NiO & PHB [poly(3-hydroxybutyrate)] nanocomposite  NiO (3%) & PHB [poly(3-hydroxy-butyrate)]/PEG (polyethylene glycol) nanocomposite  NiO (5%) & PHB [poly(3-hydroxy-butyrate)]/PEG (polyethylene glycol) nanocomposite  NiO & PHB [poly(3-hydroxybutyrate)] nanocomposite with adsorbed norfloxacin (an antibiotic)  NiO (3%) & PHB [poly(3-hydroxy-butyrate)]/PEG (polyethylene glycol) nanocomposite with adsorbed norfloxacin (an antibiotic)  NiO (5%) & PHB [poly(3-hydroxy-butyrate)]/PEG (polyethylene glycol) nanocomposite with adsorbed norfloxacin (an antibiotic) | *Escherichia coli*  *Klebsiella pneumoniae*  *Staphylococcus aureus* | Exposure to nano-Ni only on/in solidified agar; did not report measured nanoparticle or dissolved-metal concentration(s); did not report sufficient chemistry of exposure water/media; did not report control acceptability; did not report standard survival, growth, or reproduction results. |
| Ananpattarachai et al. (2016) | Environ. Sci. Pollut. Res. 23:4111-4119 | Ni-doped TiO_2_ | *Escherichia coli*  *Staphylococcus aureus* | Exposure to nano-Ni only on/in solidified agar; did not report chemical purity of the nanoparticles; did not report measured nanoparticle or dissolved-metal concentration(s); did not report sufficient chemistry of exposure water/media; did not report control acceptability. |
| Ates et al. (2016) | Water Air Soil Pollut. 227: article 70 | NiO | *Artemia salina* | Did not report standard survival, growth, or reproduction results; did not report chemical purity of the nanoparticles; did not report sufficient physical-chemical characteristics of the dry nanoparticles; did not report control acceptability. |

Table S8 (continued).

| **Authors (year)** | **Publication** | **Form of nano-Ni** | **Taxa** | **Reason(s) for not including study in meta-analysis** |
| --- | --- | --- | --- | --- |
| Baek and An (2011) | Sci. Total Environ. 409:1603-1608 | NiO | *Bacillus subtilis Escherichia coli Staphylococcus aureus*  (not *Streptococcus aureus*) | Exposure to nano-Ni only on/in solidified agar; did not report chemical purity of the nanoparticles; did not report sufficient physical-chemical characteristics of the dry or wetted nanoparticles. |
| Bagirov et al. (2019) | Inland Water Biol. 12:115-123 | Ni^0^ NiO | *Danio rerio* | Exposure via a dietborne (not waterborne) pathway; did not report standard survival, growth, or reproduction results; did not report sufficient chemistry of exposure water/media; did not report measured nanoparticle or dissolved-metal concentration(s); did not report control acceptability. |
| Bhushan et al. (2019) | Nanotechnology 30: article 185101 | NiO  NiFe2O4 | *Bacillus subtilis*  *Escherichia coli*  *Salmonella typhi*  *Staphylococcus aureus* | Did not report sufficient chemistry of exposure water/media; did not report measured nanoparticle or dissolved-metal concentration(s); did not report control acceptability; did not report sufficient physical-chemical characteristics of the wetted nanoparticles. |
| Blaise et al. (2008) | Environ. Toxicol. 23:591-598 | NiFeZn-oxide | *Hydra attenuata* MARA (Microbial array for  risk assessment) *Oncorhynchus mykiss Pseudokirchneriella subcapitata Thamnocephalus platyurus Vibrio fischeri* | Did not report an exposure concentration series and/or control; did not report sufficient chemistry of exposure water/media; did not report measured nanoparticle or dissolved-metal concentration(s); did not report control acceptability; did not report sufficient physical-chemical characteristics of the wetted nanoparticles. |
| Boran and Șaffak (2018) | Arch. Environ. Contam. Toxicol. 74:193-202 | Ni^0^ | *Danio rerio* | Did not adequately report the toxicity-test method; did not report sufficient chemistry of exposure water/media. |
| Chaudhary et al. (2015) | Adv. Mater. Lett. 6:990-998 | Ni^0^ | *Escherichia coli Klebsiella* sp. *Pseudomonas aeruginosa Staphylococcus aureus* | Exposure to nano-Ni only on/in solidified agar; did not report chemical purity of the nanoparticles; did not adequately report the toxicity-test method; did not report sufficient chemistry of exposure water/media; did not report measured nanoparticle or dissolved-metal concentration(s); did not report control acceptability; did not report sufficient physical-chemical characteristics of the wetted nanoparticles. |
| Chaudhary et al. (2019) | Heliyon 5: article e01878 | Ni^0^ | *Escherichia coli Klebsiella pneumoniae*  *Pneumonia typhus* | Exposure to nano-Ni only on/in solidified agar; did not report standard survival, growth, or reproduction results; did not report chemical purity of the nanoparticles; did not report measured nanoparticle or dissolved-metal concentration(s); did not report control acceptability; did not report sufficient physical-chemical characteristics of the wetted nanoparticles. |
| Chen et al. (2018) | Sci. Total Environ. 642:276-284 | NiFe_2_O_4_ NiFe_4_O_4_Zn | Anaerobic microbial community | Did not use a relevant test organism; did not report standard survival, growth, or reproduction results; did not report chemical purity of the nanoparticles; did not report sufficient chemistry of exposure water/media; did not report measured nanoparticle concentration(s); did not report control acceptability; did not report sufficient physical-chemical characteristics of the dry nanoparticles. |
| Cheng et al. (2019) | Sci. Total Environ. 649:995-1003 | Ni^0^  Ni-Fe_3_  Ni-Fe_5_  Ni-Fe_10_ | Bacteriophage f2 | Did not use a relevant test organism; did not report an exposure concentration series and/or control; did not report chemical purity of the nanoparticles; did not report sufficient chemistry of exposure water/media; did not report control acceptability; did not report sufficient physical-chemical characteristics of the wetted nanoparticles. |

Table S8 (continued).

| **Authors (year)** | **Publication** | **Form of nano-Ni** | **Taxa** | **Reason(s) for not including study in meta-analysis** |
| --- | --- | --- | --- | --- |
| Din et al. (2018) | Environ Nanotechnol, Monit. Manage. 9:29-36 | Ni^0^ NiO | *Bacillus subtilis Escherichia coli Pseudomonas aeruginosa* | Did not report test methods; did not report toxicity endpoint; did not report an exposure concentration series and/or control; did not report sufficient chemistry of exposure water/media; did not report measured nanoparticle or dissolved-metal concentration(s); did not report control acceptability; did not report sufficient physical-chemical characteristics of the dry or wetted nanoparticles. |
| Ezhilarasi et al. (2016) | J. Photochem. Photobiol. B: Biol. 164:352-360 | NiO | *Escherichia coli Escherichia hermannii Staphylococcus aureus Streptococcus pneumoniae* | Exposure to nano-Ni only on/in solidified agar; did not report standard survival, growth, or reproduction results; did not report an exposure concentration series and/or control; did not report chemical purity of the nanoparticles; did not report sufficient chemistry of exposure water/media; did not report measured nanoparticle or dissolved-metal concentration(s); did not report control acceptability; did not report dry size of the nanoparticles; did not report sufficient physical-chemical characteristics of the wetted nanoparticles. |
| Ezhilarasi et al. (2018) | J. Photochem. Photobiol. B: Biol. 180:39-50 | NiO | *Escherichia coli Escherichia hermannii Staphylococcus aureus Streptococcus pneumoniae* | Exposure to nano-Ni only on/in solidified agar; did not report standard survival, growth, or reproduction results; did not report an exposure concentration series and/or control; did not report chemical purity of the nanoparticles; did not report sufficient chemistry of exposure water/media; did not report measured nanoparticle or dissolved-metal concentration(s); did not report control acceptability; did not report dry size of the nanoparticles; did not report sufficient physical-chemical characteristics of the wetted nanoparticles. |
| Garza-Cervantes et al. (2019) | Int. J. Nanomed. 2019:2557-2571 | Ni with exopolysaccharide coating | *Pseudomonas aeruginosa*  *Staphylococcus aureus* | Did not report measured nanoparticle or dissolved-metal concentration(s); did not report control acceptability. |
| Golkhatmi et al. (2017) | Mater. Sci. Eng. C 78:1-11 | NiFe_2_O_4_@Ag | *Alternaria solani Bacillus subtilis Fusarium oxysporum Pseudomonas aeruginosa* | Exposure to nano-Ni only on/in solidified agar; did not report standard survival, growth, or reproduction results; did not report sufficient chemistry of exposure water/media; did not report measured nanoparticle or dissolved-metal concentration(s); did not report control acceptability; did not report sufficient physical-chemical characteristics of the wetted nanoparticles. |
| Gunsolus et al. (2017) | Environ. Sci.: Nano 4:636-346 | Ni_0.14_Co_0.14_Li_0.52_Mn_0.72_O_2_  Ni_0.23_Co_0.22_Li_0.61_Mn_0.55_O_2_  Ni_0.31_Co_0.30_Li_0.68_Mn_0.39_O_2_ | *Shewanella oneidensis* MR-1 | Did not report standard survival, growth, or reproduction results; did not report chemical purity of the nanoparticles; did not report measured nanoparticle concentration(s); did not report control acceptability. |
| Han et al. (2012) | Adv. Mater. Res. 518-523:942-945 | NiO | *Gracilaria lemaneiformis* | Did not report the source of the nanoparticles; did not report chemical purity of the nanoparticles; did not adequately report the toxicity-test method; did not report sufficient chemistry of exposure water/media; did not report measured nanoparticle or dissolved-metal concentration(s); did not report control acceptability; did not report dry size of the nanoparticles; did not report sufficient physical-chemical characteristics of the dry or wetted nanoparticles. |
| Hang et al. (2016) | Chem. Mater. 28:1092-1100 | Ni_0.33_Co_0.33_Li_0.33_Mn_0.33_O_2_ | *Shewanella oneidensis* MR-1 | Did not report sufficient chemistry of exposure water/media; did not report measured nanoparticle concentration(s). |
| Hang et al. (2018) | ACS Appl. Nano Mater. 1:1721-1730 | Ni_0.32_Co_0.35_Li_0.75_Mn_0.33_O_2_  nanosheets  Ni_0.33_Co_0.33_Li_0.92_Mn_0.34_O_2_  nanoblocks  Ni_0.33_Co_0.33_LiMn_0.33_O_2_  battery-grade powder | *Shewanella oneidensis* MR-1 | Did not adequately report the toxicity-test method; did not report measured nanoparticle concentration(s). |
| Hanna et al. (2013) | Aquat. Toxicol. 142-143:441-446 | NiO | *Leptocheirus plumulosus* | Did not use a waterborne-exposure pathway; did not report sufficient chemistry of exposure water/media; did not report measured nanoparticle concentration(s). |

Table S8 (continued).

| **Authors (year)** | **Publication** | **Form of nano-Ni** | **Taxa** | **Reason(s) for not including study in meta-analysis** |
| --- | --- | --- | --- | --- |
| He et al. (2019a) | Environ. Sci.: Nano 6:2820-2831 | Ni^0^ | Anaerobic microbial community | Did not use a relevant test organism; did not report standard survival, growth, or reproduction results; did not report chemical purity of the nanoparticles; did not report measured nanoparticle concentration(s); did not report control acceptability. |
| He et al. (2019b) | Environ. Sci.: Nano 6:1536-1548 | Ni^0^ | Anaerobic microbial community | Did not use a relevant test organism; did not report standard survival, growth, or reproduction results; did not report chemical purity of the nanoparticles; did not report sufficient chemistry of exposure water/media; did not report measured nanoparticle concentration(s). |
| Helen & Rani (2015) | Int. J. Sci. Res. 4:216-219 | Ni^0^ | *Bacillus cereus Escherichia coli Klebsiella pneumoniae Staphylococcus aureus* | Exposure to nano-Ni only on/in solidified agar; did not report the nanoparticle concentration(s); did not report measured nanoparticle or dissolved-metal concentration(s); did not report control acceptability; did not report sufficient physical-chemical characteristics of the wetted nanoparticles. |
| Hoop et al. (2016) | Adv. Func. Mater. 26:1063-1069 | Ni-coated Pd  Ni/Ag-coated Pd | *Escherichia coli Staphylococcus aureus* | Did not report sufficient chemistry of exposure water/media; did not report measured nanoparticle or dissolved-metal concentration(s); did not report sufficient physical-chemical characteristics of the dry or wetted nanoparticles. |
| Horst (2009) | 2009 NNIN REU Res. Accompl. :12-13 | NiO | *Escherichia coli* | Did not report an exposure concentration series and/or control; did not report the source of the nanoparticles; did not report chemical purity of the nanoparticles; did not adequately report the toxicity-test method; did not report measured nanoparticle or dissolved-metal concentration(s); did not report control acceptability; did not report dry size of the nanoparticles; did not report sufficient physical-chemical characteristics of the dry or wetted nanoparticles. |
| Hou et al. (2018a) | Environ. Sci. Technol. 52:7996-8004 | NiO | *Danio rerio* | Did not report chemical purity of the nanoparticles; did not adequately report the toxicity-test method; did not report sufficient chemistry of exposure water/media; did not report measured nanoparticle or dissolved-metal concentration(s); did not report sufficient physical-chemical characteristics of the dry nanoparticles. |
| Hou et al. (2018b) | Composites Part B 133:166-176 | NiO NiO-coated SiO_2_ | *Staphylococcus aureus* | Did not report an exposure concentration series and/or control; did not report the source of the nanoparticles; did not report chemical purity of the nanoparticles; did not adequately report the toxicity-test method; did not report sufficient chemistry of exposure water/media; did not report measured nanoparticle or dissolved-metal concentration(s); did not report control acceptability; did not report dry size of the nanoparticles; did not report sufficient physical-chemical characteristics of the dry or wetted nanoparticles. |
| Hrenovic et al. (2012) | Chemosphere 88:1103-1107 | NiO/clinoptile  (zeolite) | *Escherichia coli Euplotes affinis Paramecium caudatum Staphylococcus aureus* | Did not report chemical purity of the nanoparticles; did not report sufficient chemistry of exposure water/media; did not report measured nanoparticle concentration(s); did not report control acceptability. |
| Iqbal et al. (2019) | Appl. Organomet. Chem. 33: article e4950 | NiO | *Artemia salina* | Did not report chemical purity of the nanoparticles; did not adequately report the toxicity-test method; did not report sufficient chemistry of exposure water/media; did not report measured nanoparticle or dissolved-metal concentration(s); did not report control acceptability. |

Table S8 (continued).

| **Authors (year)** | **Publication** | **Form of nano-Ni** | **Taxa** | **Reason(s) for not including study in meta-analysis** |
| --- | --- | --- | --- | --- |
| Iqbal et al. (2019) | Appl. Organomet. Chem. 33: article e4950 | NiO | *Aspergillus flavus*  *Aspergillus niger*  *Bacillus subtilis*  *Candida albicans*  *Escherichia coli*  *Fusarium solani*  *Klebsiella pneumoniae*  *Mucor racemosus*  *Pseudomonas aeruginosa*  *Staphylococcus aureus* | Exposure to nano-Ni only on/in solidified agar; did not report an exposure concentration series and/or control; did not report chemical purity of the nanoparticles; did not report sufficient chemistry of exposure water/media; did not report measured nanoparticle or dissolved-metal concentration(s); did not report quantitative toxicity results; did not report control acceptability. |
| Jayaseelan et al. (2014) | Ecotoxicol. Environ. Safe. 107:220-228 | Ni^0^ | *Oreochromis mossambicus* | Did not report standard survival, growth, or reproduction results; did not report chemical purity of the nanoparticles; did not report measured nanoparticle concentration(s); did not report quantitative toxicity results. |
| Jeyaraj Pandian et al. (2016) | J. Nanopart. 2016: article 4694367 | Ni^0^ | *Aspergillus clavatus*  *Aspergillus fumigatus*  *Aspergillus niger*  *Bacillus subtilis*  *Candida albicans*  *Candida tropicalis*  *Escherichia coli*  *Klebsiella pneumoniae*  *Salmonella typhi*  *Staphylococcus epidermidis* | Did not report age, life stage, or growth stage of the organisms; did not report measured nanoparticle or dissolved-metal concentration(s). |
| Jośko et al. (2016) | J. Soils Sed. 16:1798-1808 | Ni^0^ | *Heterocypris incongruens Vibrio fischeri* | Did not use a waterborne-exposure pathway; did not report chemical purity of the nanoparticles; did not report sufficient chemistry of exposure water/media; did not report measured nanoparticle or dissolved-metal concentration(s); did not report sufficient physical-chemical characteristics of the wetted nanoparticles. |
| Kanwal et al. (2019) | Nanomaterials 9: article 309 | Ni^0^ | *Labeo rohita* | Did not report chemical purity of the nanoparticles; did not adequately report the toxicity-test method; did not report sufficient chemistry of exposure water/media; did not report measured nanoparticle or dissolved-metal concentration(s); did not report sufficient physical-chemical characteristics of the wetted nanoparticles. |
| Kganyago et al. (2018) | J. Photochem. Photobiol. B: Biol. 182:18-26 | NiO | *Enterococcus faecalis*  (not *Enterobacter*) *Escherichia coli Pseudomonas aeruginosa Staphylococcus aureus* | Did not report chemical purity of the nanoparticles; did not report sufficient chemistry of exposure water/media; did not report measured nanoparticle or dissolved-metal concentration(s); did not report sufficient physical-chemical characteristics of the wetted nanoparticles. |
| Khalil et al. (2018) | Artif. Cells, Nanomed., Biotechnol. 46:838-852 | NiO | *Aspergillus flavus Aspergillus fumigatus Aspergillus niger Mucor racemosus Rhizoctonia solani* | Exposure to nano-Ni only on/in solidified agar; did not report chemical purity of the nanoparticles; did not report sufficient chemistry of exposure water/media; did not report measured nanoparticle or dissolved-metal concentration(s); did not report control acceptability; did not report sufficient physical-chemical characteristics of the wetted nanoparticles. |

Table S8 (continued).

| **Authors (year)** | **Publication** | **Form of nano-Ni** | **Taxa** | **Reason(s) for not including study in meta-analysis** |
| --- | --- | --- | --- | --- |
| Khalil et al. (2018) | Artif. Cells, Nanomed., Biotechnol. 46:838-852 | NiO | *Bacillus subtilis Escherichia coli Klebsiella pneumoniae Pseudomonas aeruginosa Staphylococcus aureus Staphylococcus epidermidis* | Exposure to nano-Ni only on/in solidified agar; did not report standard survival, growth, or reproduction results; did not report chemical purity of the nanoparticles; did not report sufficient chemistry of exposure water/media; did not report measured nanoparticle or dissolved-metal concentration(s); did not report control acceptability; did not report sufficient physical-chemical characteristics of the wetted nanoparticles. |
| Kheiri Hafshejani et al. (2018) | Nanomed. J. 5:19-26 | Ni^0^ Ni(OH)_2_ | *Escherichia coli Klebsiella pneumoniae Staphylococcus aureus* | Did not report chemical purity of the nanoparticles; did not report sufficient chemistry of exposure water/media; did not report measured nanoparticle or dissolved-metal concentration(s); did not report control acceptability; did not report dry size of the nanoparticles; did not report sufficient physical-chemical characteristics of the dry nanoparticles. |
| Ko and Kong (2014) | Appl. Microbiol. Biotechnol. 98:3295-3303 | NiO | *Escherichia coli* | Did not report standard survival, growth, or reproduction results; did not report chemical purity of the nanoparticles; did not report sufficient chemistry of exposure water/media; did not report measured nanoparticle or dissolved-metal concentration(s); did not report dry size of the nanoparticles; did not report sufficient physical-chemical characteristics of the dry or wetted nanoparticles. |
| Ko et al. (2017) | Nanomaterials 7: article 344 | NiO NiO + Co_3_O_4_ mix NiO + CuO mix NiO + ZnO mix | *Escherichia coli* | Did not report standard survival, growth, or reproduction results; did not report an exposure concentration series and/or control; did not report chemical purity of the nanoparticles; did not report measured nanoparticle or dissolved-metal concentration(s); did not report control acceptability; did not report dry size of the nanoparticles; did not report sufficient physical-chemical characteristics of the dry or wetted nanoparticles. |
| Ko et al. (2018) | Materials 11: article 121 | NiO  NiO + CuO (mixture)  NiO + Fe_2_O_3_ (mixture)  NiO + TiO_2_ (mixture)  NiO + ZnO (mixture) | *Chlorella vulgaris* | Did not report chemical purity of the nanoparticles; did not adequately report the toxicity-test method; did not report measured nanoparticle or dissolved-metal concentration(s); did not report control acceptability; did not report sufficient physical-chemical characteristics of the wetted nanoparticles. |
| Kumar et al. (2010) | Adv. Control, Chem. Eng., Civil Eng. Mech. Eng. 2010:88-94 | Ni^0^ | *Bacillus subtilis Escherichia coli Lactobacillus* sp. *Pseudomonas aeruginosa Staphylococcus aureus* | Exposure to nano-Ni only on/in solidified agar; did not report standard survival, growth, or reproduction results; did not report chemical purity of the nanoparticles; did not report sufficient chemistry of exposure water/media; did not report measured nanoparticle or dissolved-metal concentration(s); did not report control acceptability; did not report sufficient physical-chemical characteristics of the wetted nanoparticles. |
| Lashkenari et al. (2019) | Polym.-Plast. Technol. Mater. 58:1461-1470 | NiFe_2_O_4_  NiFe_2_O_4_ @PRh (polyrhodanine) | *Escherichia coli Staphylococcus aureus* | Exposure to nano-Ni only on/in solidified agar; did not report standard survival, growth, or reproduction results; did not report an exposure concentration series and/or control; did not report chemical purity of the nanoparticles; did not adequately report the toxicity-test method; did not report sufficient chemistry of exposure water/media; did not report measured nanoparticle or dissolved-metal concentration(s); did not report control acceptability; did not report sufficient physical-chemical characteristics of the wetted nanoparticles. |
| Le et al. (2014) | Biodegradation 25:655-668 | Ni^0^ | *Agrobacterium sp.*  *PH-08* | Did not report measured nanoparticle or dissolved-metal concentration(s); did not report control acceptability. |
| Li et al. (2017) | Chem. Res. Chinese Univ. 33:107-111 | NiO | *Chlorella vulgaris* | Did not report chemical purity of the nanoparticles; did not report measured nanoparticle or dissolved-metal concentration(s); did not report control acceptability; did not report sufficient physical-chemical characteristics of the wetted nanoparticles. |

Table S8 (continued).

| **Authors (year)** | **Publication** | **Form of nano-Ni** | **Taxa** | **Reason(s) for not including study in meta-analysis** |
| --- | --- | --- | --- | --- |
| Lin et al. (2013) | Small 9:1776-1785 | NiO | *Oryzias latipes* | Did not report chemical purity of the nanoparticles; did not adequately report the toxicity-test method; did not report measured nanoparticle or dissolved-metal concentration(s); did not report sufficient physical-chemical characteristics of the dry nanoparticles. |
| Mohebbi et al. (2019) | Micro Nano Lett. 14:445-449 | NiFe_2_O_4_  NiFe_2_O_4_ @ABS (acrylonitrile butadiene styrene)  NiFe_2_O_4_ @ABS (acrylonitrile butadiene styrene)@Ag  NiFe_2_O_4_ @Ag | *Bacillus cereus*  *Escherichia coli Salmonella typhimurium*  *Staphylococcus aureus* | Exposure to nano-Ni only on/in solidified agar; did not report standard survival, growth, or reproduction results; did not report chemical purity of the nanoparticles; did not report measured nanoparticle or dissolved-metal concentration(s); did not report control acceptability; did not report sufficient physical-chemical characteristics of the wetted nanoparticles. |
| Morgaleva et al. (2015) | IOP Conf. Ser.: Mat. Sci. Eng. 98: article 012012 | Ni^0^ | *Chlorella vulgaris Danio rerio Daphnia magna Paramecium caudatum* | Did not report an exposure concentration series and/or control; did not report chemical purity of the nanoparticles; did not adequately report the toxicity-test method; did not report sufficient chemistry of exposure water/media; did not report measured nanoparticle or dissolved-metal concentration(s); did not report control acceptability; did not report sufficient physical-chemical characteristics of the dry or wetted nanoparticles. |
| Morgaleva et al. (2017) | AIP Conf. Proc. 1899: article 050004 | Ni^0^ | *Danio rerio* | Did not report chemical purity of the nanoparticles; did not report sufficient chemistry of exposure water/media; did not report measured nanoparticle or dissolved-metal concentration(s); did not report sufficient physical-chemical characteristics of the wetted nanoparticles. |
| Naik et al. (2018) | J. Mater. Sci.: Mater. Electron. 29:20395-20414 | NiAl_0.5_Fe_1.5_O_4_  NiFe_2_O_4_ | *Escherichia coli*  *Pseudomonas aeruginosa Salmonella typhimurium* | Exposure to nano-Ni only on/in solidified agar; did not report standard survival, growth, or reproduction results; did not report chemical purity of the nanoparticles; did not adequately report the toxicity-test method; did not report sufficient chemistry of exposure water/media; did not report measured nanoparticle or dissolved-metal concentration(s); did not report sufficient physical-chemical characteristics of the wetted nanoparticles. |
| Nazdar et al. (2018) | Iran. J. Sci. Technol., Trans. A: Science 42:353-361 | NiO | *Oncorhynchus mykiss* | Did not use a waterborne-exposure pathway; did not report standard survival, growth, or reproduction results; did not report sufficient chemistry of exposure water/media; did not report measured nanoparticle or dissolved-metal concentration(s); did not report control acceptability; did not report sufficient physical-chemical characteristics of the wetted nanoparticles. |
| Niemuth et al. (2019) | Environ. Sci. Technol. 53:3860-3870 | Ni_y_Li_x_Mn_z_Co_1-y-z_O_2_ | *Chironomus riparius* | Did not report measured nanoparticle concentration(s); did not report control acceptability. |
| Oukarroum et al. (2015) | BioMed Res. Int. 2015: article 501326 | NiO | *Lemna gibba* | Did not report standard survival, growth, or reproduction results; did not report the source of the nanoparticles; did not report sufficient chemistry of exposure water/media; did not report measured nanoparticle concentration(s); did not report control acceptability. |
| Oukarroum et al. (2017) | BioMed Res. Int. 2017: article 9528180 | NiO | *Chlorella vulgaris* | Did not report measured nanoparticle concentration(s); did not report control acceptability. |

Table S8 (continued).

| **Authors (year)** | **Publication** | **Form of nano-Ni** | **Taxa** | **Reason(s) for not including study in meta-analysis** |
| --- | --- | --- | --- | --- |
| Pang et al. (2009) | Chem. Commun. 2009:7542-7544 | NiO | *Bacillus subtilis Enterobacter cloacae Enterococcus faecalis Pseudomonas aeruginosa Staphylococcus aureus* | Did not report an exposure concentration series and/or control; did not report chemical purity of the nanoparticles; did not adequately report the toxicity-test method; did not report sufficient chemistry of exposure water/media; did not report measured nanoparticle or dissolved-metal concentration(s); did not report control acceptability; did not report sufficient physical-chemical characteristics of the wetted nanoparticles. |
| Perachiselvi et al. (2018) | Life Sci. Inform. Publ. 4: article 749 | NiO | *Bacillus subtilis Enterobacter* sp. *Escherichia coli* | Exposure to nano-Ni only on/in solidified agar; did not report standard survival, growth, or reproduction results; did not report an exposure concentration series and/or control; did not report chemical purity of the nanoparticles; did not report sufficient chemistry of exposure water/media; did not report measured nanoparticle or dissolved-metal concentration(s); did not report control acceptability. |
| Poornavaishnavi et al. (2019) | Appl. Surf. Sci. 483:1174-1181 | Ni^0^ | *Lepomis macrochirus* | Did not report standard survival, growth, or reproduction results; did not report age, life stage, or size of organisms; did not report sufficient chemistry of exposure water/media; did not report measured nanoparticle or dissolved-metal concentration(s); did not report control acceptability. |
| Rajakumar et al. (2013) | Vet. Parasitol. 191:332-339 | Ni^0^ | *Anopheles subpictus Culex gelidus Culex quinquefasciatus* | Did not adequately report the toxicity-test method; did not report sufficient chemistry of exposure water/media; did not report measured nanoparticle or dissolved-metal concentration(s); did not report control acceptability. |
| Rajan et al. (2017) | Mater. Res. Expr. 4: article 085030 | NiO | *Bacillus anthracis Enterobacter aerogenes Klebsiella pneumoniae Streptococcus pneumoniae* | Exposure to nano-Ni only on/in solidified agar; did not report standard survival, growth, or reproduction results; did not report an exposure concentration series and/or control; did not report chemical purity of the nanoparticles; did not report sufficient chemistry of exposure water/media; did not report measured nanoparticle or dissolved-metal concentration(s); did not report control acceptability; did not report dry size of the nanoparticles; did not report sufficient physical-chemical characteristics of the wetted nanoparticles. |
| Rajivgandhi et al. (2019) | Mater. Sci. Eng. C 102:829-843 | NiO  NiO/graphene | *Escherichia coli Pseudomonas aeruginosa* | Did not report sufficient chemistry of exposure water/media; did not report measured nanoparticle or dissolved-metal concentration(s); did not report control acceptability; did not report sufficient physical-chemical characteristics of the wetted nanoparticles. |
| Ramalingam et al. (2019) | Mater. Lett. 256: article 126616 | NiO infused into electrospun poly-ε-caprolactone/gelatin hybrid nanofibrous mat | *Escherichia coli Pseudomonas aeruginosa Staphylococcus aureus* | Did not report even nominal nanoparticle concentration(s); did not report chemical purity of the nanoparticles; did not report measured nanoparticle or dissolved-metal concentration(s); did not report control acceptability; did not report sufficient physical-chemical characteristics of the wetted nanoparticles. |
| Sabouri et al. (2019) | J. Clust. Sci. 30:1425-1434 | NiO | *Escherichia coli Pseudomonas aeruginosa Staphylococcus aureus* | Exposure to nano-Ni only on/in solidified agar; did not report standard survival, growth, or reproduction results; did not adequately report the toxicity-test method; did not report sufficient chemistry of exposure water/media; did not report measured nanoparticle or dissolved-metal concentration(s); did not report sufficient physical-chemical characteristics of the wetted nanoparticles. |
| Santhoshkumar et al. (2016) | J. Adv. Chem. Sci. 2:230-232 | NiO | *Bacillus subtilis Escherichia coli Proteus vulgaris Staphylococcus aureus* | Exposure to nano-Ni only on/in solidified agar; did not report standard survival, growth, or reproduction results; did not report even nominal nanoparticle concentration(s); did not report chemical purity of the nanoparticles; did not adequately report the toxicity-test method; did not report sufficient chemistry of exposure water/media; did not report measured nanoparticle or dissolved-metal concentration(s); did not report control acceptability; did not report sufficient physical-chemical characteristics of the wetted nanoparticles. |

Table S8 (continued).

| **Authors (year)** | **Publication** | **Form of nano-Ni** | **Taxa** | **Reason(s) for not including study in meta-analysis** |
| --- | --- | --- | --- | --- |
| Sedghi et al. (2017) | J. Alloys Comp. 729:921-928 | NiZnO/rGO/nylon-6 NiZnO/rGO/  nylon-6/Ag | *Bacillus subtilis Escherichia coli* | Did not report even nominal nanoparticle concentration(s); did not report chemical purity of the nanoparticles; did not report sufficient chemistry of exposure water/media; did not report measured nanoparticle or dissolved-metal concentration(s); did not report sufficient physical-chemical characteristics of the wetted nanoparticles. |
| Sudhasree et al. (2014) | Toxicol. Environ. Chem. 96:743-754 | Ni^0^ | *Klebsiella pneumoniae Proteus vulgaris Pseudomonas aeruginosa Staphylococcus aureus Vibrio cholerae* | Exposure to nano-Ni only on/in solidified agar; did not report standard survival, growth, or reproduction results; did not report chemical purity of the nanoparticles; did not report measured nanoparticle or dissolved-metal concentration(s); did not report control acceptability. |
| Svartz et al. (2017) | Ecotoxicol. Environ. Safe. 144:200-207 | Ni/g-Al_2_O_3_   (nano-ceramics) | *Vibrio fischeri* | Did not report standard survival, growth, or reproduction results; did not report even nominal nanoparticle concentration(s); did not report chemical purity of the nanoparticles; did not report sufficient chemistry of exposure water/media; did not report measured nanoparticle or dissolved-metal concentration(s); did not report control acceptability. |
| Udhaya et al. (2019) | Mater. Today: Proc. 8:169-175 | NiFe_2_O_4_ | *Bacillus subtilis Enterococcus faecalis Escherichia coli Pseudomonas aeruginosa* | Did not report an exposure concentration series and/or control; did not report chemical purity of the nanoparticles; did not adequately report the toxicity-test method; did not report sufficient chemistry of exposure water/media; did not report measured nanoparticle or dissolved-metal concentration(s); did not report sufficient physical-chemical characteristics of the wetted nanoparticles. |
| Wang et al. (2010) | Chemosphere 80:525-529 | NiO | *Escherichia coli* | Did not report chemical purity of the nanoparticles; did not report measured nanoparticle or dissolved-metal concentration(s); did not report control acceptability. |
| Wang et al. (2016) | J. Haz. Mat. 308:328-334 | NiO | *Photobacterium phosphoreum* | Did not report standard survival, growth, or reproduction results; did not report measured nanoparticle concentration(s); did not report control acceptability; did not report sufficient physical-chemical characteristics of the dry nanoparticles. |
| Xu et al. (2019) | Sci. Total Environ. 649:440-447 | NiO | Anaerobic microbial assemblage | Did not use a relevant test organism; did not report standard survival, growth, or reproduction results; did not report measured nanoparticle concentration(s); did not report control acceptability; did not report sufficient physical-chemical characteristics of the dry or wetted nanoparticles. |
| Zhou et al. (2018) | Chemosphere 209:163-172 | Ni^0^ | *Acartia tonsa* | Did not adequately report the toxicity-test method; did not report measured nanoparticle or dissolved-metal concentration(s); did not report control acceptability; did not report sufficient physical-chemical characteristics of the dry or wetted nanoparticles. |

Table S9. Molecular and physiological responses of organisms quantified during aqueous exposures to nanoparticulate nickel (nano-Ni). FW = freshwater; Ni^0^ = elemental nickel; NiO = nickel oxide; SW = saltwater.

| Effect | Form of nano-Ni | Taxon | References |
| --- | --- | --- | --- |
| Lipid peroxidation | Ni^0^  NiO | *Lepomis macrochirus* (FW bluegill sunfish BF-2 cells)  *Ciona intestinalis* (SW invertebrate)  *Gracilaria lemaneiformis* (SW red alga) | Poornavaishnavi et al. (2019)  Gallo et al. (2016)  Han et al. (2012) |
| Malondialdehyde production | NiO | *Artemia salina* (SW invertebrate) | Ates et al. (2016) |
| Altered enzyme activity | Ni^0^  NiO | *Lepomis macrochirus* (FW bluegill sunfish BF-2 cells)  *Danio rerio* (FW zebrafish)  None | Poornavaishnavi et al. (2019)  Lin et al. (2013) Peng et al. (2018b)  Iqbal et al. (2019) |
| Altered metabolic activity | NiO | *Pseudokirchneriella subcapitata* (FW alga)  *Saccharomyces cerevisiae* (yeast) | Sousa et al. (2018b)  Sousa et al. (2018a) |
| Glutathione depletion | Ni^0^  NiO | *Lepomis macrochirus* (FW bluegill sunfish BF-2 cells)  *Gracilaria lemaneiformis* (SW red alga) | Poornavaishnavi et al. (2019)  Han et al. (2012) |
| Increased antioxidant activity | NiO | None | Iqbal et al. (2019) |
| DNA strand breakage | Ni^0^ | *Danio rerio* (FW zebrafish) | Boran and Şaffak (2018) |
| Mitochondrial depolarization | NiO | *Gracilaria lemaneiformis* (SW red alga) | Han et al. (2012) |
| Inhibition of cell division | NiO | *Chlorella vulgaris* (FW alga)  *Pseudokirchneriella subcapitata* (FW alga) | Oukarroum et al. (2017)  Sousa et al. (2018b) |
| Decreased cell viability | NiO | *Saccharomyces cerevisiae* (yeast) | Sousa et al. (2018a) |
| Interference with embryo hatching | NiO | *Danio rerio* (FW zebrafish) | Peng et al. (2018b) |
| Alteration of electron transport system performance and photosynthesis in primary producers | NiO | *Lemna gibba* (FW plant)  *Chlorella vulgaris* (FW alga)  *Pseudokirchneriella subcapitata* (FW alga) | Oukarroum et al. (2015)  Oukarroum et al. (2017)  Sousa et al. (2018b) |

**Explanation to Figures S1 to S77**

Figures S1 to S77 show concentration-response data (and regression curves, where applicable) for exposure of aquatic organisms to nano-particulate elemental nickel (nano-Ni^0^) or nickel oxide (nano-NiO), for studies in which two or more data points are available. Results are presented in alphabetical sequence of the scientific names of the organisms.

In the captions to the figures, the median lethal concentration (EC50) and standard deviation (s.d.) refer to a sigmoid regression curve calculated using a log-probit regression option (two-parameter Gaussian tolerance distribution with logarithmic transformation of the exposure concentrations) in the United States Environmental Protection Agency’s Toxicity Relationship Analysis Program (TRAP), Version 1.30a (<https://archive.epa.gov/med/med_archive_03/web/html/trap.html>). The equation for the regression curve (in Excel programming language) is:

Response = 100*(1-0.999*NORM.S.DIST(LOG(C/EC50)/s.d.))

where C = exposure concentration (mg/L), and the units for EC50 are mg/L.

If the EC50 and s.d. are not specified (and thus a regression curve is not shown on the graph), TRAP could not converge on a solution.

Figure S1. Concentration-response relationship for 48-h hatching percentage of *Acartia tonsa* (copepod) embryos exposed to (A) sonicated or (B) non-sonicated nano-Ni^0^ (measured dry size <100 nm) [data read from Figure 7a and 7b in Zhou et al. (2016)]. Log-probit EC50 and s.d. could not be determined for either dataset.

Figure S2. Concentration-response relationship for 48-h survival of *Acartia tonsa* (copepod) embryos/nauplii exposed to (A) sonicated or (B) non-sonicated nano-Ni^0^ (measured dry size <100 nm) [data read from Figures 7c and 7d in Zhou et al. (2016)]. (A) Log-probit EC50 = 22.24 mg/L and s.d. = 0.3201; (B) log-probit EC50 = 55.11 mg/L and s.d. = 1.2584.

Figure S3. Concentration-response relationship for 168-h survival of *Acartia tonsa* (copepod) embryos/nauplii exposed to (A) sonicated or (B) non-sonicated nano-Ni^0^ (measured dry size <100 nm) [data read from Figure 8 in Zhou et al. (2016)]. (A) Log-probit EC50 = 7.813 mg/L and s.d. = 0.1714; (B) log-probit EC50 = 6.862 mg/L and s.d. = 0.1442.

Figure S4. Concentration-response relationship for (A) 96-h reproduction of *Acartia tonsa* (copepod) adults and (B) 48-h hatching percentage of the eggs, when adults and eggs were exposed to non-sonicated nano-Ni^0^ (measured dry size <100 nm) [data read from Figure 9 in Zhou et al. (2016)]. (A) TRAP could not fit a reliable regression to the data because only one partial-survival treatment was available; (B) log-probit EC50 = 15.728 mg/L and s.d. = 0.2484.

Figure S5. Concentration-response relationship for 6-h population growth of *Agrobacterium* sp. (bacteria) exposed to nano-Ni^0^ (measured dry size 44.1 nm) [data read from Figure 3c in Le et al. (2014)]. Log-probit EC50 = 162.6 mg/L and s.d. = 0.6703.

Figure S6. Concentration-response relationship for 24-h survival of *Anopheles subpictus* (mosquito) larvae exposed to nano-Ni^0^ (measured dry size = 34 nm) [data read from Figure 2 in Rajakumar et al. (2013)]. Log-probit EC50 = 4.316 mg/L and s.d. = 0.3202.

Figure S7. Concentration-response relationship for 24-h survival of *Artemia salina* (brine shrimp) larvae exposed to nano-NiO (measured dry size = 24 nm) [data read from Figure 6a in Iqbal et al. (2019)]. Log-probit EC50 = 40.87 mg/L and s.d. = 1.1574.

Figure S8. Concentration-response relationship for 48-h population growth of *Aspergillus clavatus* (fungus) exposed to nano-Ni^0^ at measured dry size of 12-36 nm [data read from Figure 3 in Jeyaraj Pandian et al. (2016)]. TRAP could not fit a reliable regression to the data because only one partial-growth treatment was available.

Figure S9. Concentration-response relationship for 48-h population growth of *Aspergillus flavus* (fungus) exposed to nano-NiO at nominal dry size of 18 nm [data read from Figure 8 in Khalil et al. (2018)]. Log-probit EC50 = 1,560.8 mg/L and s.d. = 0.6689.

Figure S10. Concentration-response relationship for 48-h population growth of *Aspergillus fumigatus* (fungus) exposed to nano-Ni^0^ at measured dry size of 12-36 nm [data read from Figure 3 in Jeyaraj Pandian et al. (2016)]. TRAP could not fit a reliable regression to the data because only one partial-growth treatment was available.

Figure S11. Concentration-response relationship for 48-h population growth of *Aspergillus fumigatus* (fungus) exposed to nano-NiO at nominal dry size of 18 nm [data read from Figure 8 in Khalil et al. (2018)]. Log-probit EC50 = 1,053 mg/L and s.d. = 0.8435.

Figure S12. Concentration-response relationship for 48-h population growth of *Aspergillus niger* (fungus) exposed to nano-Ni^0^ at measured dry size of 12-36 nm [data read from Figure 3 in Jeyaraj Pandian et al. (2016)]. TRAP could not fit a reliable regression to the data because only one partial-growth treatment was available.

Figure S13. Concentration-response relationship for 48-h population growth of *Aspergillus niger* (fungus) exposed to nano-NiO at nominal dry size of 18 nm [data read from Figure 8 in Khalil et al. (2018)]. Log-probit EC50 = 1,549.8 mg/L and s.d. = 0.3846.

Figure S14. Concentration-response relationship for 20- to 24-h population growth of *Bacillus anthracis* (bacteria) exposed to nano-NiO at nominal dry size of 14 nm [data read from Table 4 in Mishra et al. (2018)]. TRAP could not fit a reliable regression to the data because only one partial-survival treatment was available.

Figure S15. Concentration-response relationship for 6-h population growth of *Bacillus subtilis* (bacteria) exposed to nano-Ni^0^ at measured dry size of 100-300 nm [data read from Figure 4a in Peng et al. (2018a)]. A regression curve could not be fit to the data because no partial-growth treatments were available.

Figure S16. Concentration-response relationship for 24-h population growth of *Bacillus subtilis* (bacteria) exposed to nano-Ni^0^ at measured dry size of 12-36 nm [data read from Figure 2 in Jeyaraj Pandian et al. (2016)]. TRAP could not fit a reliable regression to the data because only one partial-growth treatment was available.

Figure S17. Concentration-response relationship for 24-h population growth of *Bacillus subtilis* (bacteria) exposed to nano-NiO (dry size not reported) [data read from Supplemental Figure 8D-b in Bhushan et al. (2019)]. Log-probit EC50 = 1,018 mg/L and s.d. = 0.2305.

Figure S18. Concentration-response relationship for 48-h population growth of *Candida albicans* (fungus) exposed to nano-Ni^0^ at measured dry size of 12-36 nm [data read from Figure 3 in Jeyaraj Pandian et al. (2016)]. TRAP could not fit a reliable regression to the data because only one partial-growth treatment was available.

Figure S19. Concentration-response relationship for 48-h population growth of *Candida tropicalis* (fungus) exposed to nano-Ni^0^ at measured dry size of 12-36 nm [data read from Figure 3 in Jeyaraj Pandian et al. (2016)]. TRAP could not fit a reliable regression to the data because only one partial-growth treatment was available.

Figure S20. Concentration-response relationship for 72-h population growth of *Chlorella vulgaris* (freshwater green alga) exposed to nano-NiO (nominal dry size = 20 nm) [data read from Figure 1 in Gong et al. (2011)]. Log-probit EC50 = 38.56 mg/L and s.d. = 0.2821.

Figure S21. Concentration-response relationship for 72-h population growth of *Chlorella vulgaris* (freshwater green alga) exposed to nano-NiO (nominal dry size = 20 nm) [data read from Table 1 in Li et al. (2017)]. Log-probit EC50 = 25.94 mg/L and s.d. = 0.4742.

Figure S22. Concentration-response relationship for 72-h population growth of *Chlorella vulgaris* (freshwater green alga) exposed to nano-NiO (nominal dry size = 20 nm) under identical conditions in two separate studies: data read from Figure 1 in Gong et al. (2011) and from Table 1 in Li et al. (2017). Log-probit EC50 = 32.27 mg/L and s.d. = 0.4538.

Figure S23. Concentration-response relationship for 72-h population growth of *Chlorella vulgaris* (freshwater green alga) exposed to nano-NiO (nominal dry size not reported) [data read from Figure 1a in Ko et al. (2018)]. Log-probit EC50 = 28.06 mg/L and s.d. = 0.7637.

Figure S24. Concentration-response relationship for 96-h population growth of *Chlorella vulgaris* (freshwater green alga) exposed to nano-NiO at nominal dry size of 30 nm [data read from text on page 4 in Oukarroum et al. (2017)]. Log-probit EC50 = 0.1591 mg/L and s.d. = 1.055.

Figure S25. Concentration-response relationship for 48-h survival of *Ciona intestinalis* (sea squirt) (A) sperm or (B) larvae exposed to sonicated nano-Ni^0^ (measured dry size <100 nm) [data read from Figure 1 in Gallo et al. (2016)]. (A) Log-probit EC50 = 36.12 mg/L and s.d. = 0.7009; (B) log-probit EC50 = 16.77 mg/L and s.d. = 0.4472.

Figure S26. Concentration-response relationship for 24-h survival of *Culex gelidus* (mosquito) larvae exposed to nano-Ni^0^ (measured dry size = 34 nm) [data read from Figure 2 in Rajakumar et al. (2013)]. Log-probit EC50 = 4.64 mg/L and s.d. = 0.2795.

Figure S27. Concentration-response relationship for 24-h survival of *Culex quinquefasciatus* (mosquito) larvae exposed to nano-Ni^0^ (measured dry size = 34 nm) [data read from Figure 2 in Rajakumar et al. (2013)]. Log-probit EC50 = 4.484 mg/L and s.d. = 0.3116.

Figure S28. Concentration-response relationship for 96-h survival of <1.5-hpf *Danio rerio* (zebrafish) embryos exposed to nano-Ni^0^ (nominal dry size = 5 nm) [data from Table 3 in Morgaleva et al. (2017)]. Log-probit EC50 = 211.5 mg/L and s.d. = 2.657.

Figure S29. Concentration-response relationship for 96-h survival of 24-hpf *Danio rerio* (zebrafish) embryos exposed to nano-Ni^0^ (nominal dry size = 30 nm) [data read from Figure 3 in Ispas et al. (2009), normalized to survival at lowest plotted concentration]. Log-probit EC50 = 341.9 mg/L and s.d. = 0.226.

Figure S30. Concentration-response relationship for 96-h survival of 24-hpf *Danio rerio* (zebrafish) embryos exposed to nano-Ni^0^ (nominal dry size = 60 nm) [data read from Figure 3 in Ispas et al. (2009), normalized to survival at lowest plotted concentration]. Log-probit EC50 = 359.1 mg/L and s.d. = 0.2392.

Figure S31. Concentration-response relationship for 96-h survival of 24-hpf *Danio rerio* (zebrafish) embryos exposed to nano-Ni^0^ (nominal dry size = 100 nm) [data read from Figure 3 in Ispas et al. (2009), normalized to survival at lowest plotted concentration]. Log-probit EC50 = 199.17 mg/L and s.d. = 0.2644.

Figure S32. Concentration-response relationship for 96-h survival of 24-hpf *Danio rerio* (zebrafish) embryos exposed to nano-Ni^0^ (aggregates of nominal dry size = 60 nm) [data read from Figure 3 in Ispas et al. (2009), normalized to survival at lowest plotted concentration]. Log-probit EC50 = 145.15 mg/L and s.d. = 0.5323.

Figure S33. Concentration-response relationship for 96-h survival of 72-hpf *Danio rerio* (zebrafish) embryos exposed to nano-Ni^0^ (nominal dry size = <100 nm) [data read from Figure 5 in Boran and Șaffak (2018)]. Log-probit EC50 = 91.04 mg/L and s.d. = 0.4075.

Figure S34. Concentration-response relationship for hatching success of *Danio rerio* (zebrafish) embryos exposed to nano-Ni^0^ (nominal dry size = 20 nm) [data read from Figure S3A in Özel et al. (2014)]. Log-probit EC50 = 30.77 mg/L and s.d. = 0.8348.

Figure S35. Concentration-response relationship for 96-h survival of *Danio rerio* (zebrafish) embryos exposed to nano-Ni^0^ (nominal dry size = 20 nm) [data read from Figure S5A in Özel et al. (2014)]. Survival percentages are not control-normalized because control survival was not reported. Log-probit EC50 and s.d. could not be determined.

Figure S36. Concentration-response relationship for 96-h survival of 2.5-3 hpf *Danio rerio* (zebrafish) embryos exposed to nano-NiO (nominal dry size not reported) [data read from Figure 2 in Kovrižnych et al. (2013)]. TRAP could not fit a reliable regression to the data because only one partial-survival treatment was available.

Figure S37. Concentration-response relationship for 96-h survival of 5-mo-old *Danio rerio* (zebrafish) adults exposed to nano-NiO (nominal dry size <50 nm) [data read from Figure 1 in Hou et al. (2018a)] Log-probit EC50 = 158.08 mg/L and s.d. = 0.2270.

Figure S38. Concentration-response relationship for 96-h survival of adult *Danio rerio* (zebrafish) exposed to nano-NiO (nominal dry size not reported) [data read from Figure 2 in Kovrižnych et al. (2013)]. TRAP could not fit a reliable regression to the data because only one partial-survival treatment was available.

Figure S39. Concentration-response relationship for (A) 96-h survival and (B) 30-d survival of adult *Danio rerio* (zebrafish) exposed to nano-NiO (nominal dry size <50 nm) [data read from Figure 1 in Kovrižnych et al. (2014)]. (A) Log-probit EC50 and s.d. could not be determined; (B) log-probit EC50 = 39.02 mg/L and s.d. = 0.3209.

Figure S40. Concentration-response relationship for 120-h hatching of *Danio rerio* (zebrafish) embryos exposed to nano-NiO (nominal dry size <50 nm) [data read from Figures 4 and S3 in Lin et al. (2013)]. Log-probit EC50 = 66.05 mg/L and s.d. = 1.2097.

Figure S41. Concentration-response relationship for 48-h survival of *Daphnia magna* (cladoceran) neonates exposed to nano-Ni^0^ (nominal dry size <100 nm) [data read from Figure 6 in Oleszczuk et al. (2015)]. Log-probit EC50 = 5.678 mg/L and s.d. = 1.8239.

Figure S42. Concentration-response relationship for 48-h survival of *Daphnia magna* neonates (cladoceran; <24-h old) exposed to nano-NiO at nominal dry size of <50 nm [data read from Figure 2 in Gong et al. (2016)]. Log-probit EC50 = 25.17 mg/L and s.d. = 0.5414.

Figure S43. Concentration-response relationship for 21-d reproduction of *Daphnia magna* (cladoceran; test started as embryos) exposed to nano-NiO at nominal dry sizes of (A) 10-20 nm and (B) 100 nm [data read from Figure 3 in Nogueira et al. (2015)]. (A) Log-probit EC50 = 0.3635 mg/L and s.d. = 0.3365, but the regression curve is tenuous because the magnitudes of all reproduction impairments were <25%; (B) TRAP could not converge on a solution.

Figure S44. Concentration-response relationships for 21-d reproduction of *Daphnia magna* (cladoceran; test started as embryos) exposed to nano-NiO at nominal dry size of <50 nm [data from Table 2 in Gong et al. (2016)]. (A) Average number of offspring/female; (B) average number of broods. (A) Log-probit EC50 = 0.7156 mg/L and s.d. = 0.3951; (B) log-probit EC50 = 0.5666 mg/L and s.d. = 0.4921.

Figure S45. Concentration-response relationship for 24-h population growth of *Enterococcus faecalis* (bacteria) exposed to nano-Ni^0^ at measured dry size of 25 nm [data read from Figure 7 in Kganyago et al. (2018)]. Log-probit EC50 and s.d. could not be determined.

Figure S46. Concentration-response relationship for 6-h population growth of *Escherichia coli* (bacteria) exposed to nano-Ni^0^ at measured dry size of 100-300 nm [data read from Figure 4b in Peng et al. (2018a)]. A regression curve could not be fit to the data because no partial-growth treatments were available.

Figure S47. Concentration-response relationship for 8-h population growth of *Escherichia coli* (bacteria) exposed to nano-NiO at nominal dry size of 20-30 nm [data read from Table 1 in Wang et al. (2010)]. Log-probit EC50 = 153.34 mg/L and s.d. = 1.7312.

Figure S48. Concentration-response relationship for (A) 8-h and (B) 24-h population growth of *Escherichia coli* (bacteria) exposed to nano-NiO at nominal dry size of 24 nm [data read from Table 4 and Figure 10b in Paul and Neogi (2019)]. (A) Log-probit EC50 = 4.275 mg/L and s.d. = 0.6477; (B) log-probit EC50 = 1.6486 mg/L and s.d. = 0.5631.

Figure S49. Concentration-response relationship for 24-h population growth of *Escherichia coli* (bacteria) exposed to nano-Ni^0^ at measured dry size of 12-36 nm [data read from Figure 2 in Jeyaraj Pandian et al. (2016)]. TRAP could not fit a reliable regression to the data because only one partial-growth treatment was available.

Figure S50. Concentration-response relationship for 24-h population growth of *Escherichia coli* (bacteria) exposed to nano-Ni^0^ at measured dry size of 25 nm [data read from Figure 7 in Kganyago et al. (2018)]. TRAP could not converge on a solution.

Figure S51. Concentration-response relationship for 24-h population growth of *Escherichia coli* (bacteria) exposed to nano-Ni^0^ at measured dry size of 12.5 nm [data read from Figure 4b in Argueta-Figueroa et al. (2014)]. Log-probit EC50 = 6.462 mg/L and s.d. = 1.1191.

Figure S52. Concentration-response relationship for 24-h population growth of *Escherichia coli* (bacteria) exposed to nano-NiO (dry size not reported) [data read from Supplemental Figure 8D-a in Bhushan et al. (2019)]. Log-probit EC50 = 1,028 mg/L and s.d. = 0.2536.

Figure S53. Concentration-response relationship for 24-h population growth of *Escherichia coli* (bacteria) exposed to nano-NiO at measured dry size of 4-15 nm [data read from Figure 4c in Rajivgandhi et al. (2019)]. Log-probit EC50 = 20.2 mg/L and s.d. = 0.4962.

Figure S54. Concentration-response relationship for 48-h survival of *Gracilaria lemaniformis* (marine red algae) exposed to nano-NiO (size not reported) [data read from Figure 2 in Han et al. (2012)]. Log-probit EC50 = 2.069 mg/L and s.d. = 0.5018.

Figure S55. Concentration-response relationship for 24-h population growth of *Klebsiella pneumoniae* (bacteria) exposed to nano-Ni^0^ at measured dry size of 12-36 nm [data read from Figure 2 in Jeyaraj Pandian et al. (2016)]. TRAP could not fit a reliable regression to the data because only one partial-growth treatment was available.

Figure S56. Concentration-response relationship for 24-h population growth of *Klebsiella pneumoniae* (bacteria) exposed to nano-NiO at measured dry size of 4-15 nm [data read from Figure 4d in Rajivgandhi et al. (2019)]. Log-probit EC50 = 24.16 mg/L and s.d. = 0.5266.

Figure S57. Concentration-response relationship for 24-h photosynthetic activity by *Lemna gibba* (gibbous duckweed) exposed to nano-NiO (nominal dry size = 30 nm) [data read from Figure 4c in Oukarroum et al. (2015)]. Log-probit EC50 = 315.1 mg/L and s.d. = 0.3132.

Figure S58. Concentration-response relationship for 168-h frond growth of *Lemna minor* (common duckweed) exposed to nano-NiO at nominal dry sizes of (A) 10-20 nm and (B) 100 nm [data read from Figure 2 in Nogueira et al. (2015)]. (A) Log-probit EC50 = 128.58 mg/L and s.d. = 1.2464; (B) log-probit EC50 = 4.948 mg/L and s.d. = 0.6315.

Figure S59. Concentration-response relationship for 24-h viability of *Lepomis macrochirus* BF-2 cells (fish) exposed to nano-Ni^0^ at measured dry size of 74 nm [data read from Figure 3a (MTT assay) in Poornavaishnavi et al. (2019)]. Log-probit EC50 = 31.40 mg/L and s.d. = 0.4805.

Figure S60. Concentration-response relationship for 48-h population growth of *Mucor racemosus* (fungus) exposed to nano-NiO at nominal dry size of 18 nm [data read from Figure 8 in Khalil et al. (2018)]. Log-probit EC50 = 1,388.3 mg/L and s.d. = 0.5133.

Figure S61. Concentration-response relationship for 336-h survival of *Oreochromis mossambicus* exposed to nano-Ni^0^ at measured dry size of 56 nm [data read from text in Jayaseelan et al. (2014)]. A regression curve could not be fit to the data because no partial-growth treatments were available.

Figure S62. Concentration-response relationship for 48-h (A) morphological development and (B) growth of *Paracentrotus lividus* purple sea urchin embryos/nauplii exposed to nano-Ni^0^ (measured dry size 48 nm) [data read from Figures 3 and 4 in Kanold et al. (2016)]. (A) Log-probit EC50 and s.d. could not be determined; (B) log-probit EC50 = 117.59 mg/L and s.d. = 1.4744, but the regression curve is tenuous because the magnitudes of all growth impairments were <15%.

Figure S63. Concentration-response relationship for 72-h population growth of *Pseudokirchneriella subcapitata* (freshwater green alga) exposed to nano-NiO at nominal dry sizes of (A) 10-20 nm and (B) 100 nm [data read from Figure 1 in Nogueira et al. (2015)]. (A) Log-probit EC50 = 15.853 mg/L and s.d. = 0.0941; (B) TRAP could not fit a reliable regression to the data because only one partial-survival treatment was available.

Figure S64. Concentration-response relationship for 72-h population growth of *Pseudokirchneriella subcapitata* (freshwater green alga) exposed to nano-NiO at nominal dry size of <50 nm [data read from Figure 2A in Sousa et al. (2018b)]. Log-probit EC50 = 2.108 mg/L and s.d. = 0.1961.

Figure S65. Concentration-response relationship for 24-h population growth of *Pseudomonas aeruginosa* (bacteria) exposed to nano-Ni^0^ at measured dry size of 25 nm [data read from Figure 7 in Kganyago et al. (2018)]. Log-probit EC50 = 35.841 mg/L and s.d. = 0.2786.

Figure S66. Concentration-response relationship for 48-h population growth of *Rhizoctonia solani* (fungus) exposed to nano-NiO at nominal dry size of 18 nm [data read from Figure 8 in Khalil et al. (2018)]. Log-probit EC50 = 1,073.9 mg/L and s.d. = 0.6107.

Figure S67. Concentration-response relationship for 6-h cell viability of *Saccharomyces cerevisiae* (yeast) exposed to nano-NiO at nominal dry size of <50 nm [data read from Figure 1b in Sousa et al. (2018a)]. Log-probit EC50 = 117.6 mg/L and s.d. = 0.2953.

Figure S68. Concentration-response relationship for 6-h cell viability of *Saccharomyces cerevisiae* (yeast) exposed to nano-NiO at nominal dry size of <50 nm [data read from Figure 1B in Sousa et al. (2018c)]. Log-probit EC50 = 100.96 mg/L and s.d. = 0.2897.

Figure S69. Concentration-response relationship for 24-h population growth of *Salmonella typhi* (bacteria) exposed to nano-Ni^0^ at measured dry size of 12-36 nm [data read from Figure 2 in Jeyaraj Pandian et al. (2016)]. TRAP could not fit a reliable regression to the data because only one partial-growth treatment was available.

Figure S70. Concentration-response relationship for 24-h population growth of *Salmonella typhi* (bacteria) exposed to nano-NiO (dry size not reported) [data read from Supplemental Figure 8D-d in Bhushan et al. (2019)]. Log-probit EC50 = 881.3 mg/L and s.d. = 0.2124.

Figure S71. Concentration-response relationship for (A) 8-h and (B) 24-h population growth of *Staphylococcus aureus* (bacteria) exposed to nano-NiO at nominal dry size of 24 nm [data read from Table 4 and Figure 10b in Paul and Neogi (2019)]. (A) Log-probit EC50 = 6.399 mg/L and s.d. = 0.6030; (B) log-probit EC50 = 3.308 mg/L and s.d. = 0.2693.

Figure S72. Concentration-response relationship for 24-h population growth of *Staphylococcus aureus* (bacteria) exposed to nano-Ni^0^ at measured dry size of 25 nm [data read from Figure 7 in Kganyago et al. (2018)]. Log-probit EC50 and s.d. could not be determined.

Figure S73. Concentration-response relationship for 24-h population growth of *Staphylococcus aureus* (bacteria) exposed to nano-Ni^0^ at measured dry size of 12.5 nm [data read from Figure 4a in Argueta-Figueroa et al. (2014)]. Log-probit EC50 = 12.413 mg/L and s.d. = 1.1156.

Figure S74 Concentration-response relationship for 24-h population growth of *Staphylococcus aureus* (bacteria) exposed to nano-NiO (dry size not reported) [data read from Supplemental Figure 8D-c in Bhushan et al. (2019)]. Log-probit EC50 = 1,056 mg/L and s.d. = 0.2448.

Figure S75. Concentration-response relationship for 24-h population growth of *Staphylococcus epidermidis* (bacteria) exposed to nano-Ni^0^ at measured dry size of 12-36 nm [data read from Figure 2 in Jeyaraj Pandian et al. (2016)]. TRAP could not fit a reliable regression to the data because only one partial-growth treatment was available.

Figure S76. Concentration-response relationship for 24-h population growth of *Streptococcus mutans* (bacteria) exposed to nano-Ni^0^ at measured dry size of 12.5 nm [data read from Figure 4c in Argueta-Figueroa et al. (2014)]. Log-probit EC50 = 11.13 mg/L and s.d. = 1.0442.

Figure S77. Concentration-response relationship for 48-h population growth of five fungal species (*Aspergillus flavus*, *A. fumigatus*, *A. niger*, *Mucor racemosus*, and *Rhizoctonia solani*) exposed to nano-NiO at nominal dry size of 18 nm [data read from Figure 8 in Khalil et al. (2018)]. Log-probit EC50 = 1,316.3 mg/L and s.d. = 0.5819.
